# A modular transcriptome map of mature B cell lymphomas

**DOI:** 10.1186/s13073-019-0637-7

**Published:** 2019-04-30

**Authors:** Henry Loeffler-Wirth, Markus Kreuz, Lydia Hopp, Arsen Arakelyan, Andrea Haake, Sergio B. Cogliatti, Alfred C. Feller, Martin-Leo Hansmann, Dido Lenze, Peter Möller, Hans Konrad Müller-Hermelink, Erik Fortenbacher, Edith Willscher, German Ott, Andreas Rosenwald, Christiane Pott, Carsten Schwaenen, Heiko Trautmann, Swen Wessendorf, Harald Stein, Monika Szczepanowski, Lorenz Trümper, Michael Hummel, Wolfram Klapper, Reiner Siebert, Markus Loeffler, Hans Binder, for the German Cancer Aid consortium Molecular Mechanisms for Malignant Lymphoma

**Affiliations:** 1 Interdisciplinary Centre for Bioinformatics, Universität Leipzig, Härtelstr. 16–18, 04107 Leipzig, Germany; 2 Institute for Medical Informatics, Statistics and Epidemiology, Universität Leipzig, Härtelstr. 16–18, 04107 Leipzig, Germany; 3 Group of Bioinformatics, Institute of Molecular Biology, National Academy of Sciences, 7 Hasratyan str, 0014 Yerevan, Armenia; 4 Institute of Human Genetics, University Hospital Schleswig-Holstein, Arnold-Heller Str. 3, 24105 Kiel, Germany; 5 Institute of Pathology, Kantonal Hospital St. Gallen, Rorschacher Str. 95, 9007 St. Gallen, Switzerland; 6 Hematopathology Lübeck, Maria-Goeppert-Str. 9a, 23562 Lübeck, Germany; 7 Institute of Pathology, University Hospital Frankfurt, Theodor-Stern-Kai 7, 60590 Frankfurt, Germany; 8 AstraZeneca, Tinsdaler Weg 183, 22880 Wedel, Germany; 9 Institute of Pathology, University Hospital of Ulm, Albert-Einstein-Allee 23, 89081 Ulm, Germany; 10 Institute of Pathology, University Hospital Würzburg, Josef-Schneider-Str. 2, 97080 Würzburg, Germany; 11 Department of Pathology, Robert-Bosch-Hospital, Auerbachstr. 110, 70376 Stuttgart, Germany; 12 Second Medical Department, University Hospital Schleswig-Holstein, Arnold-Heller Str. 3, 24105 Kiel, Germany; 13 Ortenau Hospital Offenburg-Gengenbach, Ebertpl. 12, 77654 Offenburg, Germany; 14 Internal Medicine III, University Hospital of Ulm, Albert-Einstein-Allee 23, 89081 Ulm, Germany; 15 Hospital Esslingen, Hirschlandstr. 97, 73730 Esslingen a. N, Germany; 16 Pathodiagnostik, Komturstr. 58-62, 12099 Berlin, Germany; 17 Department of Hematology and Oncology, Georg-August University, Robert-Koch-Str. 42, 37077 Göttingen, Germany; 18 Institute of Pathology, Charité Universitätsmedizin, Charitéplatz 1, 10117 Berlin, Germany; 19 Hematopathology Section, University Hospital Schleswig-Holstein, Arnold-Heller Str. 3, 24105 Kiel, Germany; 20 Institute of Human Genetics, University Hospital of Ulm, Albert-Einstein-Allee 23, 89081 Ulm, Germany

**Keywords:** Tumor heterogeneity, B cell malignancies, Gene regulation, Molecular subtypes, Machine learning

## Abstract

**Background:**

Germinal center-derived B cell lymphomas are tumors of the lymphoid tissues representing one of the most heterogeneous malignancies. Here we characterize the variety of transcriptomic phenotypes of this disease based on 873 biopsy specimens collected in the German Cancer Aid MMML (Molecular Mechanisms in Malignant Lymphoma) consortium. They include diffuse large B cell lymphoma (DLBCL), follicular lymphoma (FL), Burkitt’s lymphoma, mixed FL/DLBCL lymphomas, primary mediastinal large B cell lymphoma, multiple myeloma, IRF4-rearranged large cell lymphoma, MYC-negative Burkitt-like lymphoma with chr. 11q aberration and mantle cell lymphoma.

**Methods:**

We apply self-organizing map (SOM) machine learning to microarray-derived expression data to generate a holistic view on the transcriptome landscape of lymphomas, to describe the multidimensional nature of gene regulation and to pursue a modular view on co-expression. Expression data were complemented by pathological, genetic and clinical characteristics.

**Results:**

We present a transcriptome map of B cell lymphomas that allows visual comparison between the SOM portraits of different lymphoma strata and individual cases. It decomposes into one dozen modules of co-expressed genes related to different functional categories, to genetic defects and to the pathogenesis of lymphomas. On a molecular level, this disease rather forms a continuum of expression states than clearly separated phenotypes. We introduced the concept of combinatorial pattern types (PATs) that stratifies the lymphomas into nine PAT groups and, on a coarser level, into five prominent cancer hallmark types with proliferation, inflammation and stroma signatures. Inflammation signatures in combination with healthy B cell and tonsil characteristics associate with better overall survival rates, while proliferation in combination with inflammation and plasma cell characteristics worsens it. A phenotypic similarity tree is presented that reveals possible progression paths along the transcriptional dimensions. Our analysis provided a novel look on the transition range between FL and DLBCL, on DLBCL with poor prognosis showing expression patterns resembling that of Burkitt’s lymphoma and particularly on ‘double-hit’ MYC and BCL2 transformed lymphomas.

**Conclusions:**

The transcriptome map provides a tool that aggregates, refines and visualizes the data collected in the MMML study and interprets them in the light of previous knowledge to provide orientation and support in current and future studies on lymphomas and on other cancer entities.

**Electronic supplementary material:**

The online version of this article (10.1186/s13073-019-0637-7) contains supplementary material, which is available to authorized users.

## Background

Germinal center-derived B cell lymphomas are tumors of the lymphoid tissues representing one of the most heterogeneous malignancies in terms of their molecular and cellular phenotypes [1]. Frequent B cell lymphomas in adulthood are follicular lymphomas (FL) and diffuse large B cell lymphomas (DLBCL), and, in children, Burkitt’s lymphomas (BL). Especially DLBCL show a very heterogeneous spectrum of phenotypes as revealed by morphological [2], immunohistochemical [3] and metabolic [4] characteristics. Particularly, molecular high-throughput analytics created many ways to disentangle the diversity of this disease into a series of stratification schemes [5–14].

The German Cancer Aid MMML (Molecular Mechanisms in Malignant Lymphoma) consortium collected altogether more than 800 biopsy specimens of mature B cell lymphomas and about 100 samples of tumor cell lines, normal B cell populations and non-neoplastic tonsil tissue serving as different kinds of reference, and recorded their genome-wide transcriptomes by means of microarrays. The B cell lymphomas studied comprise virtually the whole spectrum of this disease. Previous studies published subgroups of samples selected from this cohort to extract a molecular classifier that distinguishes BL from ‘other than BL’ cases [7], to disentangle DLBCL into subclasses [10], to associate DLBCL cases with selected signaling pathway activities [8] and to study other partial aspects of this disease [7, 8, 10, 15–18]. An integrated and comprehensive analysis of all samples including about 200 hitherto unpublished cases is presented here.

We hereby aim at establishing a map of the expression landscape of B cell lymphomas covering the heterogeneity of their molecular expression states. Heterogeneity of lymphomas can be understood as a series of mutually similar molecular states forming a continuum without clear-cut borderlines not only between different DLBCL entities but also with respect to the distinction between DLBCL, FL and, partly, also BL [7, 19]. These in many respects indistinct characteristics of the tumors can reflect overlapping genetic events such as the chromosomal translocation of the *MYC* gene which represents the genetic hallmark of BL but which also appears in about 5–10% of DLBCL leading to expression phenotypes resembling BL [20] and considered as a separate subtype according to the WHO classification [21]. The continuum of molecular states can also reflect the underlying stages of B cell development affected by cancer initiation and progression, e.g. in the course of histological transformations from FL to DLBCL after the consecutive accumulation of a series of genetic hits [22].

Previously, we have developed an omics ‘portraying’ method using self-organizing map (SOM) machine learning [23, 24] which was applied to a series of data types and diseases [24–29]. SOM portraying takes into account the multidimensional nature of gene regulation and pursues a modular view on co-expression, reduces dimensionality and supports visual perception in terms of individual, case-specific ‘omics’ portraits. By applying SOM portraying on B cell lymphoma transcriptomes, we demonstrate that multidimensional profiling will permit a description of the molecular heterogeneity of this disease in terms of a continuous spectrum of transcriptional states and to visualize them by means of different maps distinguishing lymphoma subtypes and their functional context and to link them to prognosis. The transcriptome map will provide a tool that aggregates, refines and visualizes the data collected in the MMML study and interprets them in the light of previous knowledge to provide orientation and support in current and future studies.

## Methods

### Lymphoma samples, genetic analyses and expression data

The gene expression data set consists of 913 samples studied by means of Affymetrix HG-U133A GeneChip microarrays. They divide into reference samples (tumor cell lines, sorted B cells, tonsils), mature B cell lymphomas and other tumors collected in the study (see Additional file 1: Table S1 and Additional file 2 for details). One of the lymphoma specimens was measured twice on two arrays. Tumors were diagnosed in panel meetings of the MMML pathology group. Genetic analyses by means of interphase fluorescence in situ hybridization were performed on frozen or paraffin-embedded tissues with the use of probes for IGH, IGK, IGL, MYC, BCL6 and BCL2. Loci in which MYC was fused to IGH, IGK or IGL were referred to as ‘IG-MYC’. Lymphomas with MYC breakpoints without fusion of MYC to an IG locus were called ‘non-IG-MYC’ (see [7] for details). Reference data included different lymphoma cell lines [30, 31], several B cell types isolated either from peripheral blood (pre- and post-germinal center (GC) B cells) or from suspended tonsillar tissue (GC B cells), and tonsillar tissue specimen for comparison of their expression patterns with those of lymphoma as specified in Additional file 1: Table S1.

### SOM expression portraying

Gene expression data were preprocessed using hook calibration, quantile normalization and centralization as described in [23, 32]. The preprocessing detects and corrects for possible outlier samples, batch effects and a sample- and transcript-specific background in cancer data [29, 33] (Additional file 1: Figure S1). Preprocessed expression data were then clustered using self-organizing map (SOM) machine learning which translates the expression data matrix consisting of *N* = 22,283 probe set values covering 13,182 ensemble genes, and *M* = 913 samples, into a data matrix of reduced dimensionality where the *N* gene expression profiles are represented by *K* = 2500 metagene profiles. Hereby, ‘profile’ denotes the vector of *M* expression values per gene/metagene. The SOM training algorithm distributes the *N* genes over the *K* metagenes using the Euclidian distance between the expression profiles as a similarity measure. It ensures that genes with similar profiles cluster together in the same or in closely located metagenes. Each metagene profile can be interpreted as the mean profile averaged over all gene profiles referring to the respective metagene cluster. The metagene expression values of each sample are visualized by arranging them into a two-dimensional 50 × 50 grid and by using maroon to blue colors for maximum to minimum expression values in each of the portraits. The number of genes typically varies from metagene to metagene and ranges from only a few associated single genes to metagenes containing more than a hundred genes (see the population map in Additional file 1: Figure S2a). This way, our approach portrays the transcriptome landscape of each sample in terms of a colored image visualizing its metagene expression values. Group- and subtype-specific mean portraits were generated by averaging the portraits of all cases belonging to one group/subtype. We used the implementation of the method in the Bioconductor R-package ‘oposSOM’ [34].

### Sample diversity analyses, spot module detection, gene maps and enrichment analysis

Metagenes of similar profiles cluster together forming ‘spot-like’ red and blue areas of over- and under-expression in the portraits due to the self-organizing properties of the SOM. The spot patterns are characteristic fingerprints of each particular sample enabling us to compare their transcriptomic landscapes by means of diversity analysis using a graph representation called ‘correlation network’ and phylogenetic tree visualization as implemented in ‘oposSOM’ [34]. The spot patterns of the expression portraits reveal clusters of correlated metagenes (Additional file 1: Figure S2d) which collect the associated single genes into modules of co-expressed genes. These modules were defined by segmentation of the map according to an over-expression criterion, collecting adjacent metagenes which exceed 90% of maximum metagene expression in the respective sample class (see also [23, 32] and Additional file 1). The number of spot modules detected represents an intrinsic characteristic of the co-expression network present in the samples. The size of the SOM, *K*, was chosen to ensure the robust identification of spots by exceeding their number by more than two orders of magnitude as was demonstrated previously [28]. The spots are characterized by their number distributions and by spot co-occurrence networks based on association rules [35]. We additionally performed zoom-in SOM analyses for selected subsets of samples (lymphoma cell lines, B cells and Burkitt’s lymphomas) to validate resolution of the transcriptomic landscape [23].

We applied gene set enrichment analysis to the lists of genes located in each of the spot modules to discover their functional context using right-tailed Fisher’s exact test [36, 37]. The gene set enrichment *Z*-score (GSZ) was used to evaluate the expression profiles of the gene sets across the samples of the study [32, 38]. Gene maps visualize the position of selected genes within the SOM grid. According to their location in or near a specific spot, one can deduce over- and under-expression characteristics and the potential functional context of the respective gene. Its position is invariant in all expression portraits, which allows for direct comparison.

### Pattern types

The sample portraits were stratified into pattern types (PATs), where a PAT is defined by the combination of spot modules over-expressed in the respective samples. Rare PATs found in less than five cases per subtype were rejected from further analysis to focus on recurrent pattern types solely. A sample that shows no expression module activated is still assigned to a PAT if their module expression values correlate with those of a certain PAT with Pearson correlation coefficient *r* > 0.8. Otherwise, it is assigned to ‘no PAT’ and labeled as ‘∅’. In total, 679 samples (74%) were classified into PATs according to detected spots, 102 (11%) were additionally classified by the correlation step, and 133 (15%) remain unclassified. PAT-specific mean expression portraits are generated as averages over the individual sample portraits of the respective PAT.

### Metagene sets of hallmarks of cancer

The hallmarks of cancer constitute a series of biological capabilities commonly acquired by tumors [39]. We assembled eight metagene sets referring to the hallmarks angiogenesis, controlling genomic instability, glucose energetics, inflammation, invasion and metastasis, proliferation and replicative immortality and resisting death according to the hallmark definitions proposed in ref. [40]. Each of these hallmark sets collects from 2 to 12 suited gene sets taken from our repository of gene sets. The lists of gene sets included in each hallmark set are provided in Additional file 1: Table S3.

### Cell type and pathway signal flow analyses, and survival analyses

Immune cell composition of the tumor biopsies was estimated from the expression data using the program CIBERSORT based on support vector regression and previous knowledge on purified leukocyte expression profiles [41]. Pathway activity was analyzed using the pathway signal flow method as implemented in oposSOM [42].

Hazard ratios and *p* values for pairwise comparisons of survival curves were derived utilizing Cox models. The models were additionally adjusted by inclusion of co-factors ‘chemotherapy’ (yes/no) and ‘Rituximab’ (yes/no). Cases without information about therapy were removed from the multivariate model. The prognostic map was generated as follows: For each metagene, lymphoma cases with available survival information were divided into cases showing expression of this metagene above or below the 50% percentile, respectively, and then compared using a Cox model. This way, hazard ratios (HRs) were obtained for all metagenes and visualized in terms of a map using blue to red colors for low to high HRs.

## Results

### SOM portraits of lymphoma subtypes

The gene expression data set studied here was generated by the German MMML consortium. It consists of biopsy specimens of mature B cell lymphomas, of other tumor cases such as multiple myeloma (MM), of lymphoma cell line specimen (32 samples of 28 different lymphoma cell lines), of sorted B cell populations (30) and of non-neoplastic tonsil tissue samples (10) which were used as reference for comparison of their expression landscapes with that of the lymphomas (see Additional file 1: Table S1). Expression data were complemented by pathological evaluation of tissue samples, genetic and immuno-histochemical analyses and clinical data. The tumor samples were divided into ten major strata based on pathological evaluation, genetic and/or previous gene expression classification criteria (see Additional file 1: Table S1 for details), namely, (i) diffuse large B cell lymphoma (DLBCL, 430 cases), (ii) follicular lymphoma (FL, 145), (iii) intermediate lymphoma according to [7] (81), (iv) prototypic Burkitt’s lymphoma (BL, 74), (v) mixed FL/DLBCL and WHO grade 3b FL (48), (vi) mediastinal large B cell lymphoma (PMBL, 23), (vii) multiple myeloma (MM, 20), (viii) IRF4-rearranged large cell lymphoma (IRF4-LCL, 10), (ix) MYC-negative Burkitt-like lymphomas with a chr. 11q aberration pattern (mnBLL-11q, 6) and (x) mantle cell lymphoma (MCL, 4). DLBCL were further stratified into the germinal center (GCB, 142), activated B cell (ABC, 133), unclassified (97) DLBCL and double-hit (DH, 58) lymphoma and, alternatively, into plasmablastic, centroblastic, anaplastic and immunoblastic DLBCL based on pathological panel diagnosis [43, 44]. FLs were divided according to BCL2-break (positive, negative and NA) and according to tumor grading (1, 2 and 3a). Intermediate lymphomas were split into BL-like (11) and others (70).

**Fig. 1:**
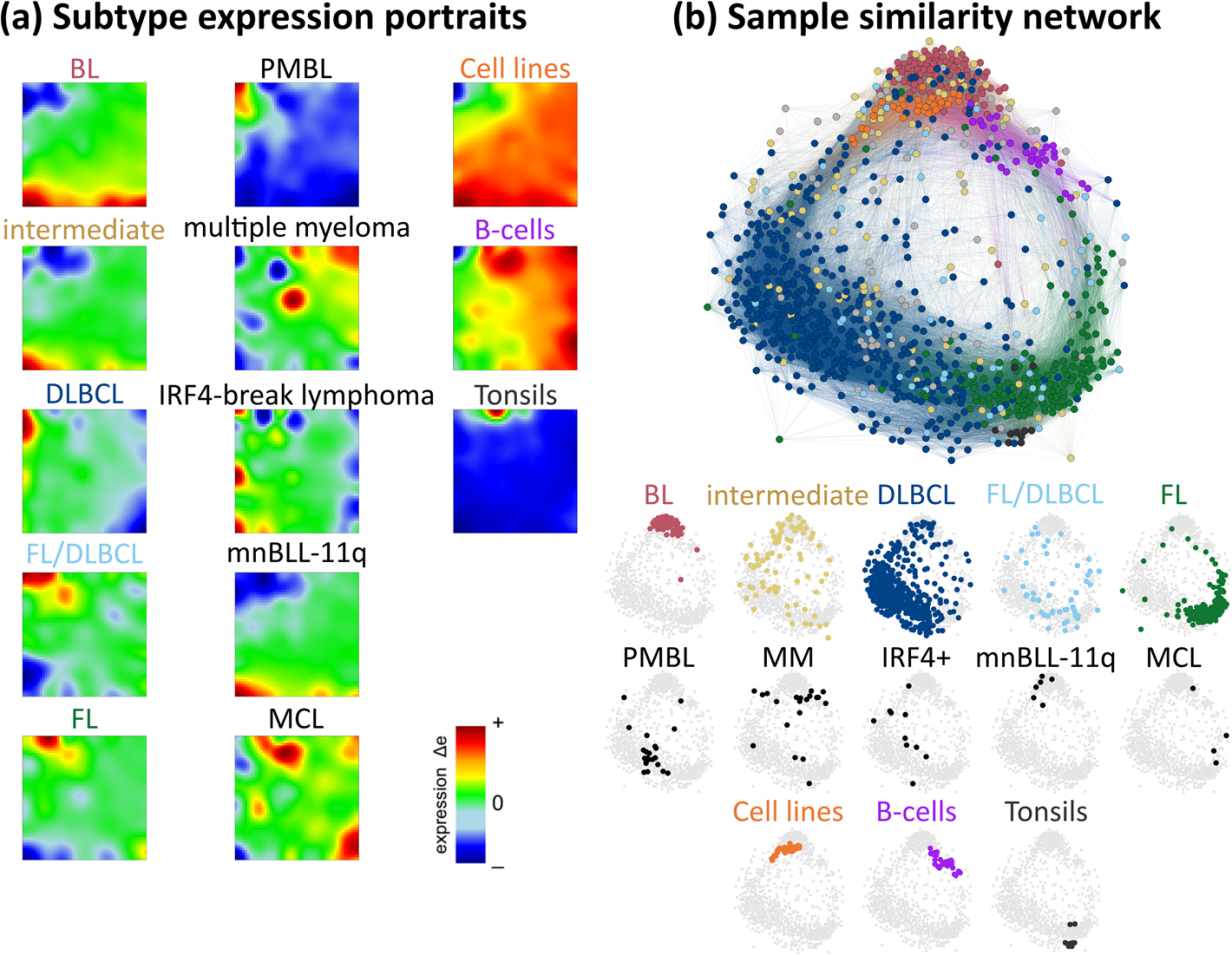
Expression and sample landscapes of lymphoma subtypes. **a** Mean expression portraits of the major B cell lymphoma subtypes and of the controls are characterized by red-blue spot patterns which reflect clusters of co-expressed genes up- and downregulated in the subtype on the average, respectively. The complete gallery of individual sample portraits is available in Additional file 3. **b** The correlation network visualizes the similarity relations between the samples as an undirected graph. The nodes represent the samples and are colored according to their class membership. The edges connect sample pairs whose expression landscapes are mutually correlated with Pearson’s correlation coefficients larger than 0.5. The small networks in the part below highlight each individual class considered. Part of the lymphoma types and of the controls occupy localized areas (e.g. BL and tonsils), while other types distribute over wider regions (e.g. intermediate lymphomas and FL/DLBCL) thus reflecting a more heterogeneous composition of the respective groups

The expression data of all samples were used to train a self-organizing map (SOM) which provides ‘portraits’ of the transcriptomic landscape of each individual sample (see Additional file 3 for the whole gallery of the expression portraits), and, after averaging, mean portraits of the different strata considered (Additional file 1: Figure S3). The mean transcriptomic portraits of the lymphoma strata (i)–(x) are shown in Fig. 1a together with the mean portraits of reference samples. The mean portraits reveal unique spot-like patterns of over- (colored in red) or under-expressed (in blue) gene clusters but also partly overlapping spots, e.g. between BL, mnBLL-11q and, partly, intermediate lymphoma and between DLBCL, PMBL and, partly, IRF4-LCL and FL. The correlation network visualizes the heterogeneity of the samples (Fig. 1b): BL cases (red-colored nodes) aggregate into a dense cloud which reflects relatively close similarity between them while the DLBCL cases (blue nodes) form an extended, widely distributed data cloud due to the heterogeneous character of this subtype. It overlaps with the cluster of FL cases (green nodes), thus forming a continuum ranging from BL-related to FL-related expression patterns. The samples of the three reference systems accumulate in localized regions of the similarity network, reflecting relatively homogenous expression patterns contrary to most of the lymphoma subtypes (Fig. 1b). They comprise different lymphoma cell lines and B cell types (Additional file 1: Table S1) showing however relatively similar SOM portraits (Additional file 1: Figure S3). We provided a detailed analysis of these reference systems and of BL in terms of zoom-in SOM analyses and class-related difference portraits in the supplementary text (Additional file 1: Figures S17 - S19). The zoom-in SOM maps partly provide an enhanced resolution of the expression landscapes of the particular subsystems. However, comparison with the results of all samples presented here confirms sufficiently high resolution of this analysis (Additional file 1: Figures S17 - S19). In summary, SOM portraying provides subtype-specific images that visualize their expression landscapes in terms of clusters of over- and under-expressed genes.

### Spot modules partition the expression map

**Fig. 2:**
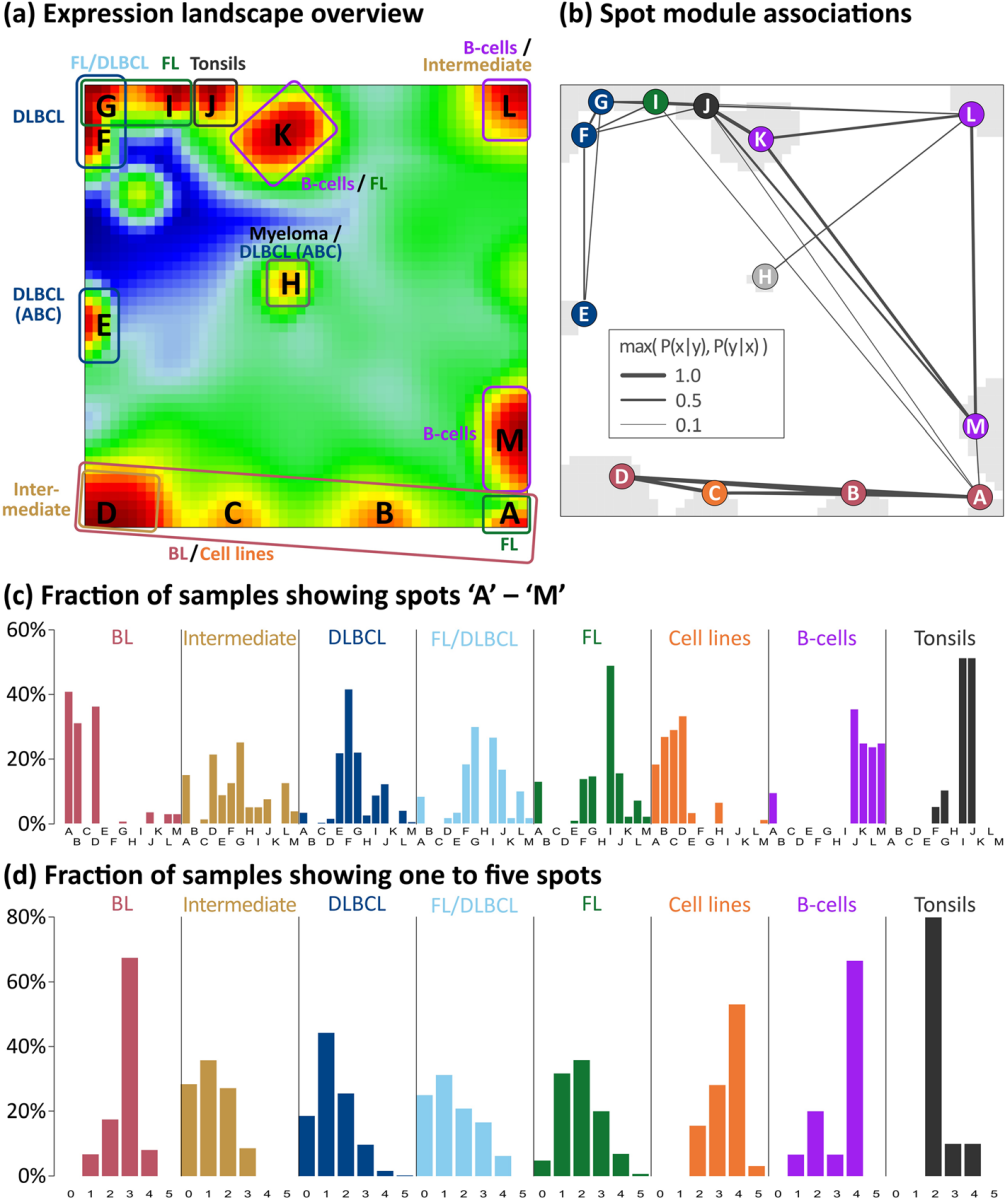
Decomposing the expression landscape of lymphomas into spot modules of co-expressed genes. **a** The overview map collects all differentially expressed modules observed in the subtype-specific portraits into one map. The sample class(es) expressing the respective spot module(s) is/are assigned in the figure, thus segmenting the landscape into regions typically upregulated in certain lymphoma subtypes. Spot modules were labeled by capital letters A–M. Dark red/blue areas refer to over-/under-expression, respectively. **b** Probabilities of concerted module activation show that diverse sets of spot modules, e.g. A, B and D, are frequently upregulated in concert. Notably, spot A often appears also together with spot I, which is characteristic for double-hit lymphomas (see also Additional file 1: Figure S4). The color of the module labels represents the corresponding lymphoma subtype. **c** Spot-class association histograms depict the fraction of samples showing a certain spot in each class. It indicates, for example, that spots A, B and D are prevalent in the BL portraits in agreement with the assignments shown in panel **a**. **d** The spot number histograms show the fraction of samples with one, two etc. over-expression spots in each class. It reveals, that in most BL samples three spots can be observed, while DLBCL and FL/DLBCL show a broader variability of spots. Only the five most abundant lymphoma strata (i)–(v) are shown

We generated an over-expression-spot map which summarizes all red over-expression spots observed in the single-sample portraits (Fig. 2a, see [23]). In total, 13 spot modules A–M were identified, where each of them represents a module of co-expressed genes with a specific mean expression profile (Additional file 1: Figure S5; for lists of genes, see Additional file 4). Nine of the spots are mainly activated in the lymphomas and four in the controls. The spot-connectivity map in Fig. 2b visualizes the probability of joint spot appearances in the single-sample portraits. Accordingly, BL samples frequently express spots A, B and D together (red circles) while DLBCL tend to co-express E–G (blue circles). The frequency distribution of activated spots and their number distribution in each class show two-to-four recurrently activated modules in BL, cell lines, B cells and tonsils (Fig. 2c, d). For example, tonsils are characterized by ubiquitous presence of the two spots I and J (see also the tonsil portrait in Fig. 1a), which are specifically over-expressed in tonsillar tissue specimen as well as in tumors contaminated with tonsillar tissue this way giving rise to the ‘blue-shift’ of the rest of the portrait (Additional file 1: Figure S3 and S5) [33]. The broader distribution in intermediate lymphoma, DLBCL and FL reflects their more heterogeneous character. No spots were assigned in 133 samples, mainly in DLBCL (77 samples), intermediate lymphoma (24), FL (7), FL/DLBCL (11) and BL (2) due to their relatively flat expression landscapes.

### A functional map of the spot modules

**Fig. 3:**
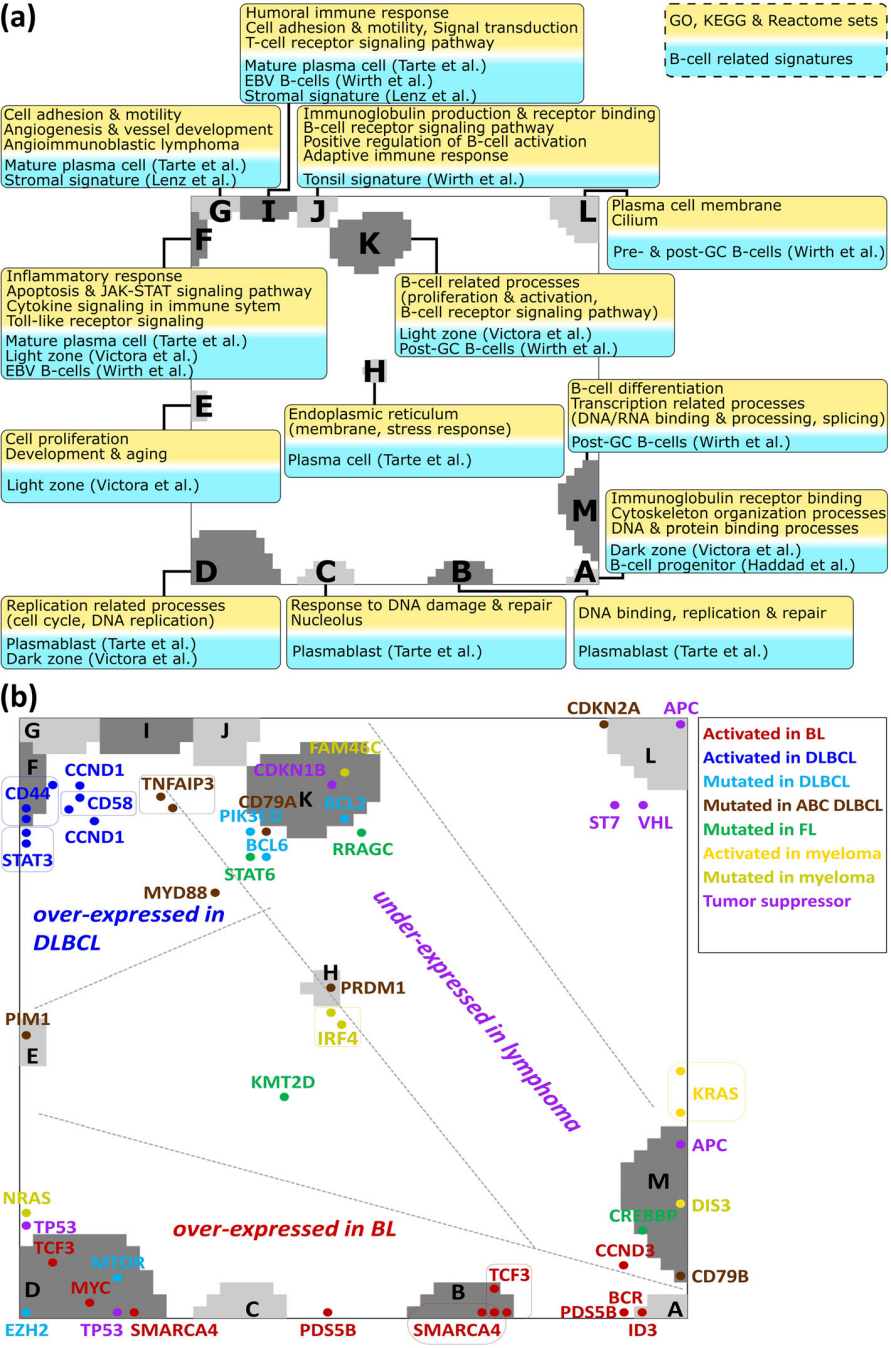
Functional analysis of the expression modules. **a** Enriched gene sets from GO, KEGG and Reactome databases (yellow background; *p* < 0.05, Fisher’s exact test) and of B cell-related signatures taken from [47–49] and from a separate analysis of our B cell samples (blue background) are assigned to each of the spot modules. For example, spots A and F associate with cell-cycle activity and inflammation, respectively. **b** Mapping of key genes mutated in lymphomas and multiple myeloma taken from [50–60] into the expression landscape: Most of the genes accumulate in or near the spot modules thus reflecting a subtype-specific modulation of their gene expression. Multiple appearances of gene names refer to different Affymetrix probe sets

Each of the 13 spot clusters is populated typically with a few hundred genes (Additional file 4). Their functional context was analyzed by gene set analysis [32] (Fig. 3a and Additional file 1: Figures S7–S9). Modules activated in BL tumors are related to ‘replication’ and ‘cell cycle’ (spot D, *p* values < 10^− 25^ in Fisher’s test) and those in DLBCL to ‘inflammation’ (spot F, < 10^− 25^) reflecting tumor-infiltrating immune cells [13, 45, 46]. Modules G and I show stromal signatures [9] while module J upregulated in tonsils significantly enriches gene sets related to ‘keratinization’ (< 10^− 23^), a ‘tonsil signature’ (< 10^− 10^) [23, 32], and to ‘B cell-mediated adaptive immune response’ (< 10^− 11^). Genes associated with biological functions of B cells are enriched in modules K (e.g. ‘B cell activation’) and M (‘B cell differentiation’, < 10^− 3^). For a more detailed assignment of the spot patterns to B cell biology, we estimated enrichment of a series of gene sets taken from literature [47, 48] and from a separate analysis of the B cell samples (Fig. 3a, boxes with blue background). Modules activated in BL accumulate signature genes of the dark zone of the GC whereas modules activated in DLBCL accumulate light zone signature genes. The modules H, K, L and M enrich genes related to ‘plasma cells’ and to ‘pre/post-GC B cells’, respectively. Hence, assigning the functional context of the spot patterns provides a functional map that enables interpretation of the lymphoma portraits in terms of activated cellular programs.

### Mapping key mutations

Mapping of selected genes with mutations in lymphoma [50, 51, 53–60] into the SOM associates their expression profiles with that of the adjacent expression modules (Fig. 3b). Genes frequently mutated in BL are located in the BL-specific spot A (e.g. ID3, CCND3) and D (e.g. TCF3, SMARCA4, MYC) indicating their increased activity in BL and partly in intermediate lymphomas [50, 61]. Genes frequently mutated in DLBCL, FL and/or multiple myelomas (MM) such as BCL6 and BCL2 are found in or near spot K upregulated in healthy B cells and, to a lesser degree, in FL, and downregulated in BL and DLBCL (Additional file 1: Figure S5). The chromatin-modifying genes CREBBP (mutated in 30% of GCB-DLBCL [11], in early FL stages [62] and shared between primary and transformed FL [63]) and KMT2D (alias MLL2) are located in spots up- or downregulated in part of the FL cases compared with DLBCL suggesting epigenetic deregulation in FL. It presumably also involves HLA class II antigens [64], as supported by genome-wide association study (GWAS) analyses (Additional file 1: Figure S12), and MYD88, CDKN2B and PIK3CD, all affected by mutations preferentially in ABC-DLBCL leading to ‘chronic active’ B cell receptor signaling [11] (see also Additional file 1: Figure S11 for pathway analyses).

**Fig. 4:**
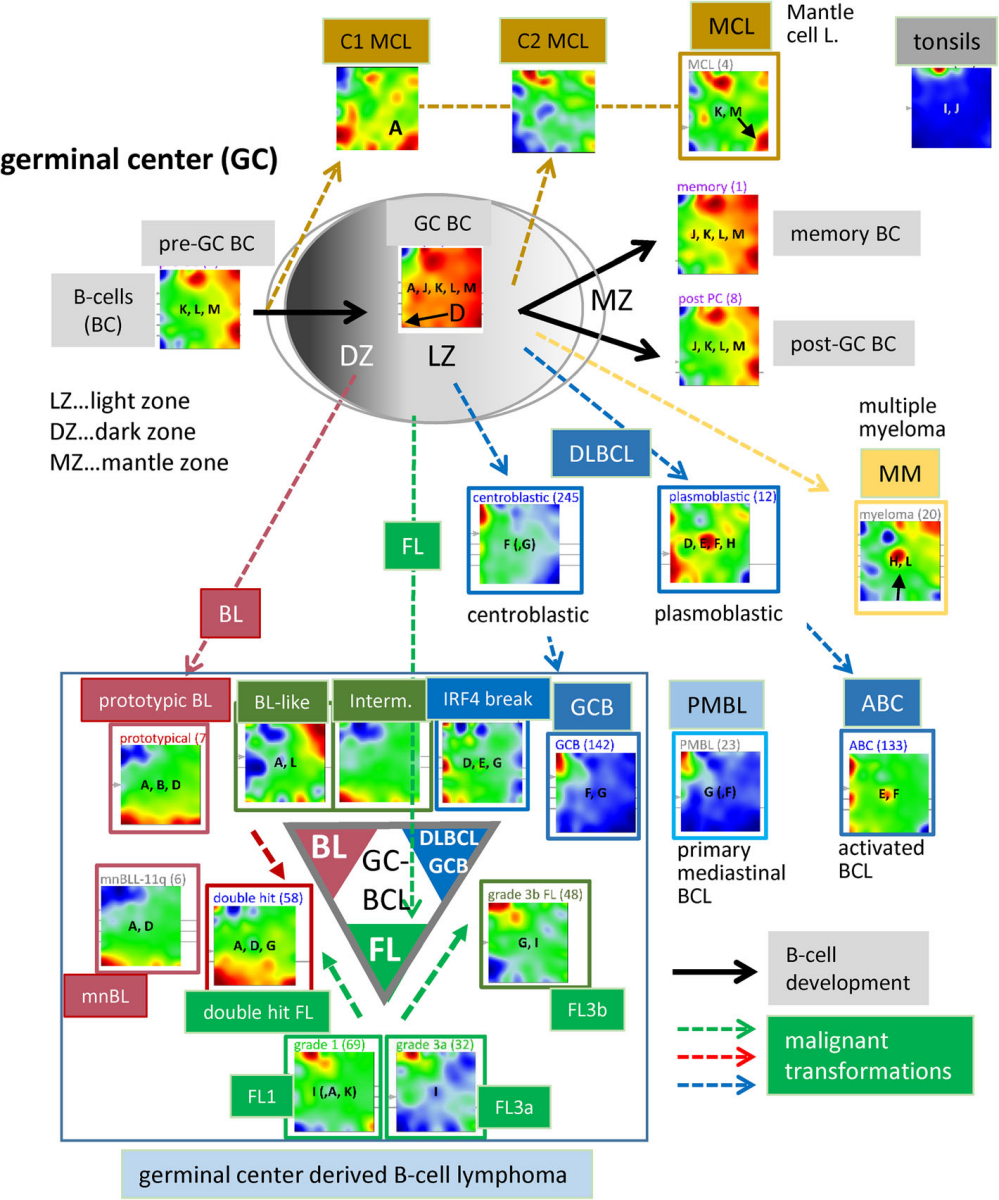
Expression portraits of B cells and lymphomas, and their relation with respect to the GC biology. See also Additional file 1: Figure S3 for the full gallery of group-related expression portraits. Activated spot combinations are given as letters in the portraits. The particular spot patterns observed for the different lymphoma subtypes can be related to their functional context and associated key genes (compare with Fig. 2). For example, DZ-related types such as BL are proliferative as indicated by upregulation of spot D, which is on a lower expression level in LZ-related DLBCL. ABC-DLBCL and MM activate spot H, which is, in turn, virtually inactive in BL, GCB-DLBCL and FL

Spot H, specifically upregulated in MM and immunoblastic and plasmablastic DLBCL, co-regulates with PRDM1 (alias BLIMP1) promoting plasma cell differentiation by repressing MYC activity [53]. PRDM1 is deactivated in GCB-DLBCL and presumably also other subtypes by mutations, deletions or epigenetic effects [65, 66]. Interestingly, also IRF4 co-regulates with PRDM1 as indicated by its co-location in spot H [11]. The PIM1 oncogene (spot E) is over-expressed in most ABC-DLBCL [63] and in transformed FL (about 50% of patients) with ABC characteristics but it is rarely mutated in primary FL (less than 10%) [65]. Interestingly, both genes, PIM1 (40% in ABC vs 15% in GCB) and PRDM1 (25% vs less than 5%), show high prevalence of activating mutations in ABC-DLBCL [14] as indicated by over-expression of spot modules E and H in the SOM portrait of ABC-DLBCL but not in GCB-DLBCL (see Fig. 4).

We also mapped hereditable risk genes for DLBCL and/or FL which were identified by GWAS (Additional file 1: Figure S12). These genes accumulate near the spots related to the somatic mutations in DLBCL and FL. In summary, mapping of mutations into the expression landscapes directly associates genomic with transcriptional events and allows linking mutations with their possible effects on the different subtypes.

### Expression portraits relate to the pathogenesis in the GC

The scheme in Fig. 4 illustrates the relation between the expression portraits of B cells and of lymphoma subtypes and GC biology [52] (see also Additional file 1: Figure S3). B cells simultaneously express the spots J (tonsil signature), and K, L and M as characteristic B cell-specific signatures (Fig. 3a). In contrast to pre- and post-GC B cells, GC B cells over-express spot D that reflects activated proliferation in the dark zone of the GC. Also the portraits of the cancer cell line specimen over-express this proliferation signature (Fig. 1). On the other hand, all cell line systems under-express spot F related to inflammation because of the absence of immunogenic bystander cells. For a more detailed view, we refer to the ‘zoom-in’ SOM analysis provided in the supplementary text (Additional file 1: Figure S17 and S 18).

DLBCL of the GCB and ABC types show common expression of spot F (inflammation), but they differ in the expression of spots containing the key genes MYC (spot D), PIM1 (E) and PRDM1 (H) (see Fig. 4 and previous subsection). The portrait of PMBL closely resembles GCB-DLBCL, which differs from that of ABC-DLBCL. It specifically expresses the plasma cell-related spot H and the proliferation-related spot D. Interestingly, the ABC-type portrait resembles that of plasmablastic and partly also immunoblastic DLBCL while the portraits of anaplastic and centroblastic DLBCL partly agree with that of GCB lymphoma (Additional file 1: Figure S3), where plasmablastic, immunoblastic, anaplastic and centroblastic lymphoma annotate three morphological variants of DLBCL. Spot H shows prominent expression also in multiple myelomas (MM) accompanied by deactivation of BCL6-related transcriptional programs (spot K) as a hallmark of plasma cell maturation which is further paralleled by high expression of spot L reflecting B cell-like characteristics. On the other hand, MM under-express spots D, E and F due to decreased proliferative and inflammatory properties compared with ABC-DLBCL. Interestingly, IRF4-LCL over-express spots D, E and G thus indicating a combination of BL-like (spot D), stromal (spot G) and ABC-DLBCL (spot E) characteristics (Fig. 4). BL-like intermediate lymphomas show over-expression of spot B that accumulates marker genes of BL [7] but also of spot L which is related to post- and pre-GC B cell characteristics. This spot is not observed in prototypic BL and possibly refers to early stages of BL development which is supported by the relatively weak expression of spot D harboring proliferation-related genes such as MYC, TP53 and EZH2 (Fig. 3b). The portrait of mnBLL-11q closely resembles that of intermediate lymphomas and only partly that of prototypic BL [67] which, in turn, resembles that of double-hit lymphoma (DHL, Fig. 4). In the supplemental text, we present a comprehensive analysis of the expression patterns before and after acquiring a second hit combining MYC- with BCL2 or BCL6 translocations (Additional file 1: Figure S4). It illustrates the capability of SOM portraying to identify specific transcriptional patterns. The DZ- (spots D and A) GC signatures were evident in BL, while the LZ-GC signature (spots E–G) was found in GCB-DLBCL, partly FL and also in ABC-DLBCL and intermediate lymphomas in mixed amounts.

FLs of all histological grades express spot I as a transcriptional hallmark of this subtype independent of the presence or absence of the genetic hallmark of FL, namely the t(14;18) translocation (BCL2-break). Spot I partly transforms into spot G with increasing grade of FL paralleled by decreasing gene activities in the regions of other spots which indicates the progressive dominance of FL characteristics over other processes such as DNA processing and B cell characteristics. Grade 3b FL (FL/DLBCL) show a combined pattern of the FL and DLBCL-specific spots I and F, respectively, indicating the continuous transformation from FL into DLBCL. The portrait of double-hit lymphoma resembles that of BL thus reflecting increased transcriptional activity compared with FL (see also Additional file 1: Figure S4 for details). The portrait of MCL shows a unique pattern different from all the other lymphoma groups but sharing similarities with the portraits of B cells especially with strong expression of spot K and, partly, of spot M. MCL split into two subtypes deriving from pre- (type C1) or post-GC memory (C2) B cells, respectively [68]. Both types carry the t(14:18) translation giving rise to over-expression of spot I also found in FL. C1 MCL, in contrast to C2 MCL, express the gene SOX11 near spot A which prevents them from entering the GC. The portrait of tonsils expresses spot J as the unique prominent characteristics.

In summary, stratification of the molecular subtype portraits with respect to histological and genetic diagnosis reveals detailed relations to GC biology such as DZ- and LZ-GC, plasma cell and B cell characteristics. Overall, the criteria used, however, do not provide a consensus with respect to the classification of the tumors.

### Pattern types

**Fig. 5:**
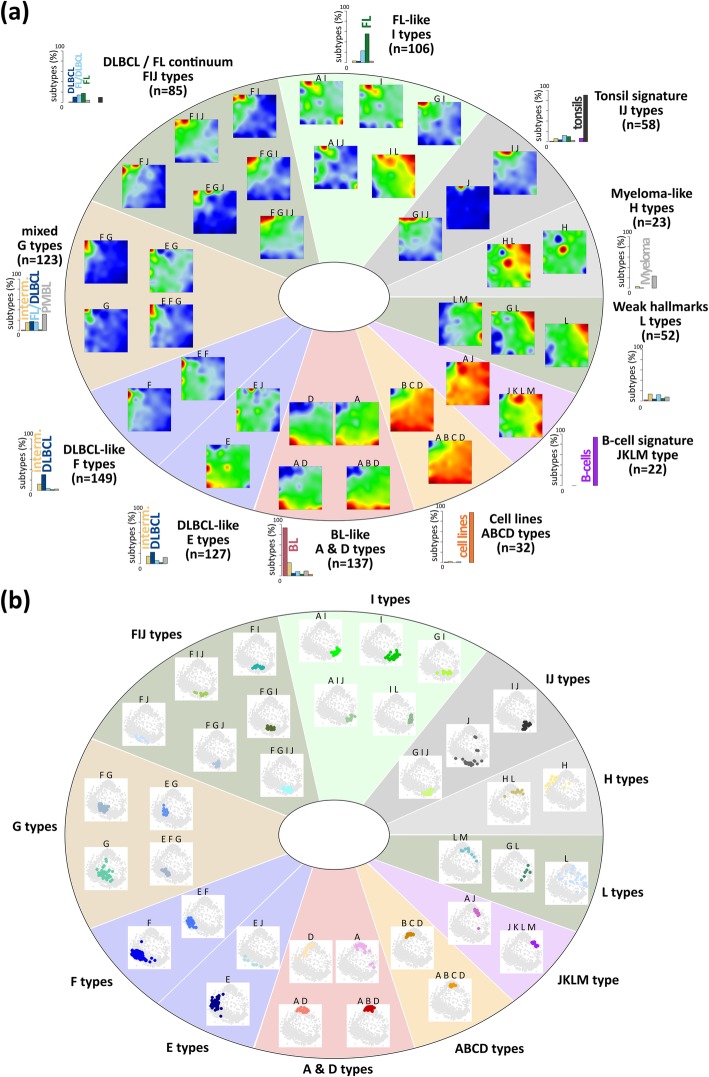
Expression (**a**) and sample (**b**) landscapes of the lymphoma pattern types (PATs). PATs were arranged into 11 groups. For each group, number-frequencies of samples diagnosed in the major histological lymphoma subtypes are given as barplot in panel **a** (see also the enrichment heatmap in Additional file 1: Figure S5). Each group collects similar and largely overlapping spot patterns. They arrange into dense sample clouds in the similarity networks, what is in contrast to the partly heterogeneous subtypes (compare with Fig. 1b)

All subclasses express a combination of spots which makes them suited candidates as landmarks in the expression landscape of lymphoma. To address this multi-dimensionality, we define ‘pattern types’ (PATs) as the combination of spot modules concertedly over-expressed in a sample. We use notations such as ‘A B D’ to annotate cases jointly over-expressing the three modules A, B and D. In total, we identified 35 different PATs where 30 of them refer to lymphomas (Fig. 5a). We further stratified the PATs into 11 PAT groups, where the groups were labeled according to the most characteristic overlapping module(s) of the respective PATs (Fig. 5a). For example, BLs accumulate within five PATs collected into one BL-like group, while DLBCL distribute over four groups with 14 PATs, where one of these groups overlaps with FL. DLBCL were assigned to proliferative PATs with ABC-DLBCL characteristics (E type) or inflammatory and stromal types with GCB-DLBCL characteristics (F and G types, respectively). FL and FL/DLBCL are found in two groups mainly over-expressing spot I and partly also G and F thus forming a continuum between DLBCL and FL expression patterns. Interestingly, a small subgroup of intermediate lymphomas and of FL forms the L type that shares similarities with multiple myeloma (H type), partly expressing plasma cell programs associated with spot H. High expression of spot J indicates contaminations of the lymphoma samples with non-neoplastic tonsillar tissue. They were clustered together with the tonsils showing spot J as a hallmark. B cells divide into two PATs, which accumulate either GC B cells (‘AJ’) or pre/post-GC B cells (‘JKLM’, see also Additional file 1: Figure S3). The samples of each PAT mostly aggregate into compact data clouds in the similarity net which confirms the homogeneous character of their expression landscapes (Fig. 5b).

In summary, PATs and PAT groups provide an expression-driven stratification of lymphoma and reference samples with enhanced resolution and homogeneity compared with the histological subtypes and with reference to activated cellular programs.

### Characteristics of the PATs

**Fig. 6:**
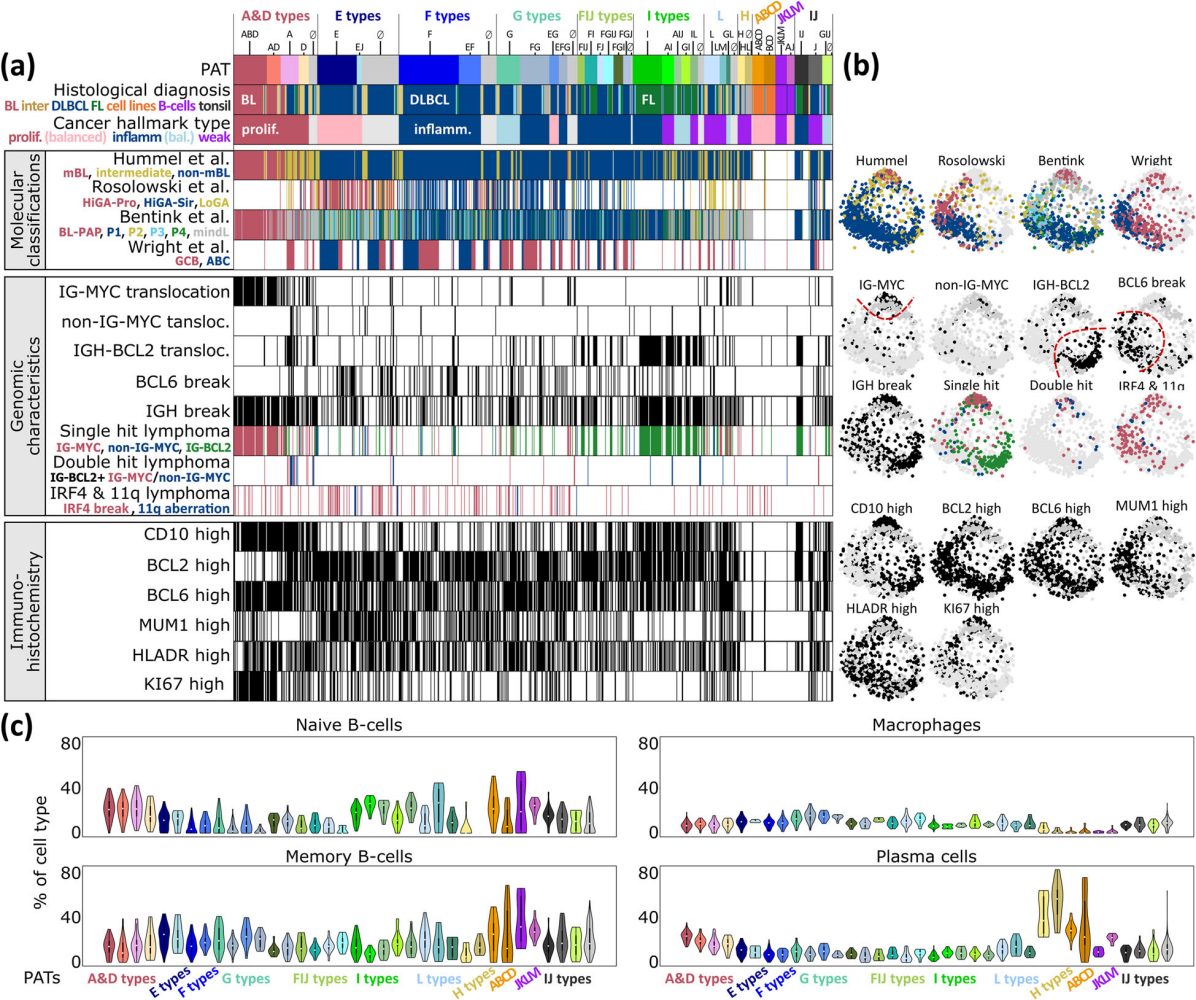
Characterization of lymphoma pattern types (PATs). **a** For each lymphoma patient, the PAT, clinical characteristics, previous molecular classifications, genomic characteristics and immunohistochemical (IHC) phenotypes are indicated in the barplots. Thresholds for classification of IHC markers are described in [17]. **b** Mapping of cases showing selected characteristics into the correlation network. It shows, e.g. that different previous classifications of lymphomas, such as ABC and GCB-DLBCL, accumulate in different areas of the network, which, in turn, associate with certain PATs. **c** Percentage of selected leukocyte cells according to their mRNA signatures across the PATs. ‘No PAT’ samples were assigned as ‘∅’ and distributed into PAT groups using a minimum *Euclidian* distance between sample and mean group portraits

The plot in Fig. 6a associates selected patient and functional characteristics with the PATs. The BL-related PATs show typical characteristics of this subtype such as the increased incidence in young patients, the presence of an IG-MYC translocation, low expression of BCL2 and a high percentage of KI67-positive highly proliferating cells [7]. DLBCL PATs enrich in older patients with high expression levels of the BCL2 markers and slower proliferation as seen by KI67. Expression modules activated in PATs of BL and FL reflect different transcriptional programs associated with IG-MYC and IG-BCL2 single hits, respectively. The joint appearance of both aberrations in double-hit lymphomas (DHL) specifically activates spot module A (PAT ‘A’) in agreement with recently published DHL expression signatures [69, 70] (Additional file 1: Figure S4c). Hence, the combination of different translocations in double-hit lymphomas does not necessarily combine the spot patterns of the respective single-hit lymphomas, but instead, they can induce new, non-additive expression patterns.

We related the PATs to expression signatures of previous lymphoma classifications schemes [6–8, 10]. As expected, samples of the mBL and non-mBL subtypes [7] show strong correspondence with BL and DLBCL, respectively. The intermediate class (by Hummel et al.) accumulates in the PATs expressing spots A and D but also in the I-type typical for FL which reflects its heterogeneity. This class tends to collect DLBCL with BL resemblance induced, e.g. by IG-BCL2 and IG-MYC translocations, respectively (Additional file 1: Figure S4a). It also collects virtually all double-hit lymphomas, which enrich in PAT ‘A’ as described above. DLBCL tumors with the ABC signature [6] significantly enrich in the PATs ‘E’, ‘F’ and ‘E F’, collecting 75 of all 183 ABC cases (41%, *p* value < 10^− 15^; see also the expression portrait of ABC lymphoma in Fig. 4) which associates them with a distinct molecular PAT signature. GCB-DLBCL express predominantly PATs of the G and FIJ types. The classification of Rosolowski et al. [10] shows correspondence with E-, F- and L-type PATs. It reveals enrichment of the HiGA-Pro (high gene activation with proliferative phenotype) class in PATs ‘E’ (*p* value < 10^− 14^) and ‘E J’ (*p* value < 0.005) that also enriches ABC-DLBC (see above), suggesting relevant involvement of spot module E genes in this classifier. LoGA (low gene activity) cases accumulate in PAT ‘L’ which associates with B cell characteristics and thus possibly with early stages of lymphoma development (*p* values < 0.005, see Fig. 3a). Inflammatory [45] and stromal [9] signatures associate with PATs containing spots F, G or I, respectively (Additional file 1: Figure S8). We also compared our transcriptomic strata with recently established genetic classes of DLBCL [12, 14] by mapping characteristic mutations and chromosomal aberrations into the expression landscape. It turned out that these genetic classes associate with different PAT types covering the expression spectrum ranging from phenotypes of BL resemblance, over ABC and GCB-DLBCL, to FL-like tumors (Additional file 1: Figure S10).

Next, we estimated the percentage of selected immune cells based on their mRNA content in the tumor transcriptomes using CIBERSORT [41] (Fig. 6c). The transcriptomes of BL and partly of intermediate lymphomas (A- and D-type PATs) reflect characteristics of naïve B cells while DLBCL transcriptomes are more related to memory B cells which reflects a higher maturation grade of the B cells upon neoplastic transformation into DLBCL compared with BL. H-type PATs enriching MM show a high abundance of a plasma cell mRNA signature. Tumor-infiltrating macrophages are detected in considerable amounts in DLBCL and FL (F- and G-type PATs) which overall reflects a changing tumor microenvironment with PAT resolution. Previous studies report similar results, however, with lower resolution on a subtype level for BL, DLBCL, FL and MM [71]. Altered B cell receptor signaling in B cell lymphomas [11] will possibly lead to changed immune cell signatures with possible consequences for digital immune cell decomposition. In summary, the PATs can be associated with different functional categories and they show correspondence with previous lymphoma classifications and leukocyte characteristics. The PAT approach thus provides a classification scheme based on a multidimensional understanding of the expression landscape of this disease.

### Cancer hallmark types

**Fig. 7:**
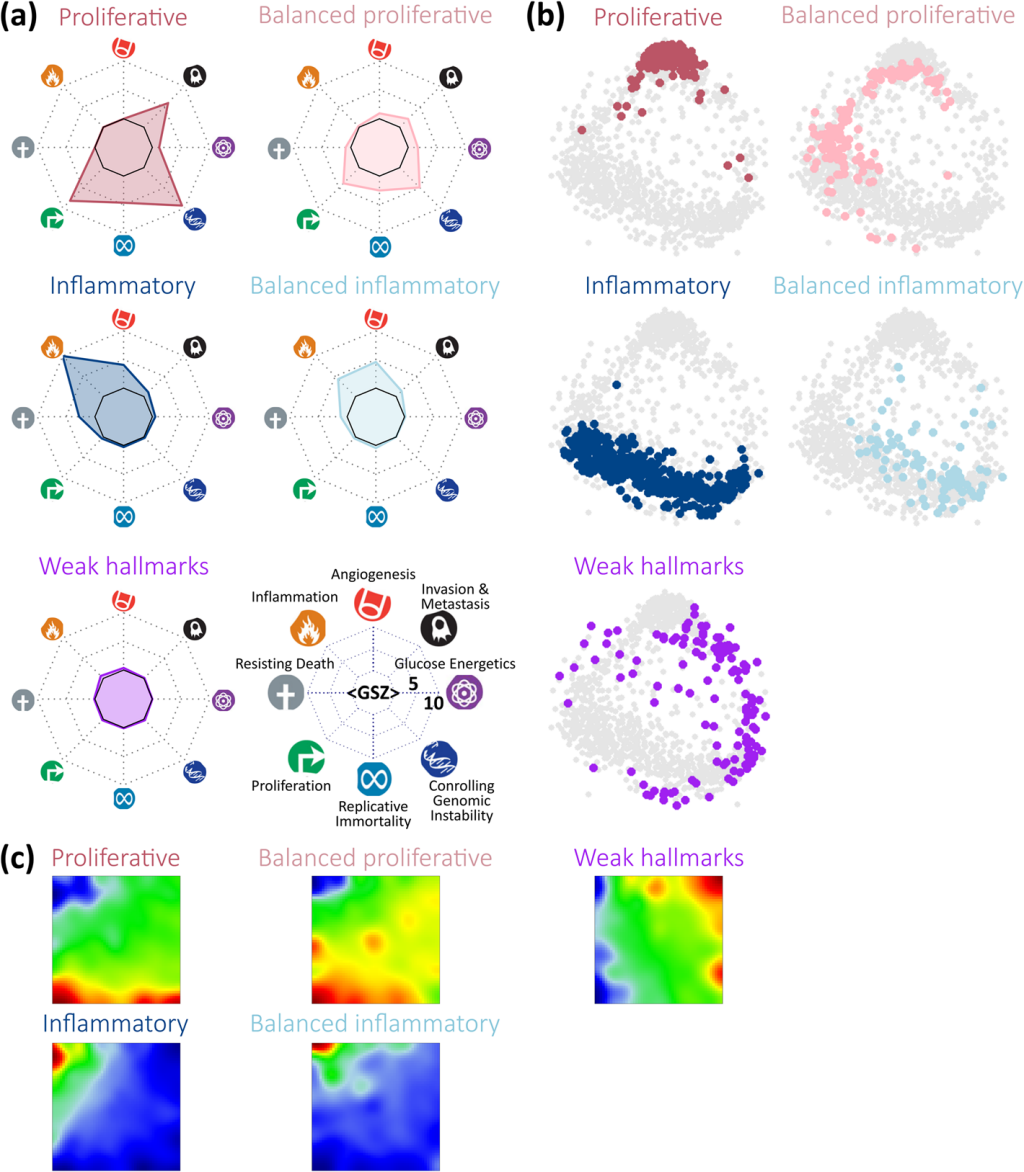
Cancer hallmark types (HT) were characterized using an expression signature for each of the eight hallmarks and clustering of the lymphoma samples into five HT. **a** The expression levels of the hallmark signatures were presented in terms of a polar plot (hallmark diagrams) for each of the HTs. Each hallmark is assigned to one polar axis as indicated in the legend. HTs differ markedly regarding the hallmarks ‘inflammation’ on the one hand and ‘proliferation’ and ‘invasion and metastasis’ on the other hand. **b** Samples assigned to each of the five HTs were colored in the correlation network, where each dot represents one sample. It reveals that the proliferative, inflammatory and weak HTs occupy three different, mutually separated regions while the two balanced HTs fill the transition zones in between them. **c** Mean expression portraits of the HTs reveal different regions of over- and under-expression, which can be directly compared with the portraits of the subtypes (Fig. 1a) and PATs (Fig. 5)

For a more generalized assignment of the PATs, we make use of a cancer hallmark scheme [40]. We defined eight hallmark signatures using GO and literature-gene sets, applied them to each PAT and represented its hallmark signature in terms of a polar diagram (Additional file 1: Figures S13 and S14). The PATs were then grouped into five hallmark types (HTs, see Fig. 7): (i) The *proliferative* HT with activated hallmark proliferation, controlling genetic instability, invasion and metastasis and, partly, regenerative immortality, collects mainly BL and intermediate lymphoma with over-expressed spots A, B and D. (ii) The *balanced proliferative* HT with a moderate activation of the hallmark proliferation and a reduced level of invasion and metastasis collects intermediate lymphoma and DLBCL over-expressing spots D, E and H including ABC-DLBCL. (iii) The *inflammatory* HT with the activated hallmark ‘inflammation’ contains DLBCL especially of the GCB type, FL and, to a lesser degree, DLBCL/FL expressing spots E, F and partly G. (iv) The *balanced inflammatory* HT with reduced activity of ‘inflammation’ and dominating hallmark ‘angiogenesis’ due to the over-expression of spots G and I collects mainly DLBCL/FL; (v) The *weakly carcinogenic* HT with generally low overall hallmark activities which collects lymphoma showing partly healthy B cell characteristics. Note that the hallmark ‘angiogenesis’ associates mainly with spot G that enriches stromal [9] and also inflammatory [45] characteristics (Additional file 1: Figure S13c). The samples assigned to each HT occupy almost distinct regions of the similarity net thus reflecting homogeneous expression landscapes (Fig. 7b). Their over-expression spot patterns shift along the edges of the map due to mutual similarities between the HTs (Fig. 7c). Hence, the concept of cancer hallmarks coarsens the expression characteristics and provides a simplified stratification scheme of lymphomas.

### Prognostic HR map

**Fig. 8:**
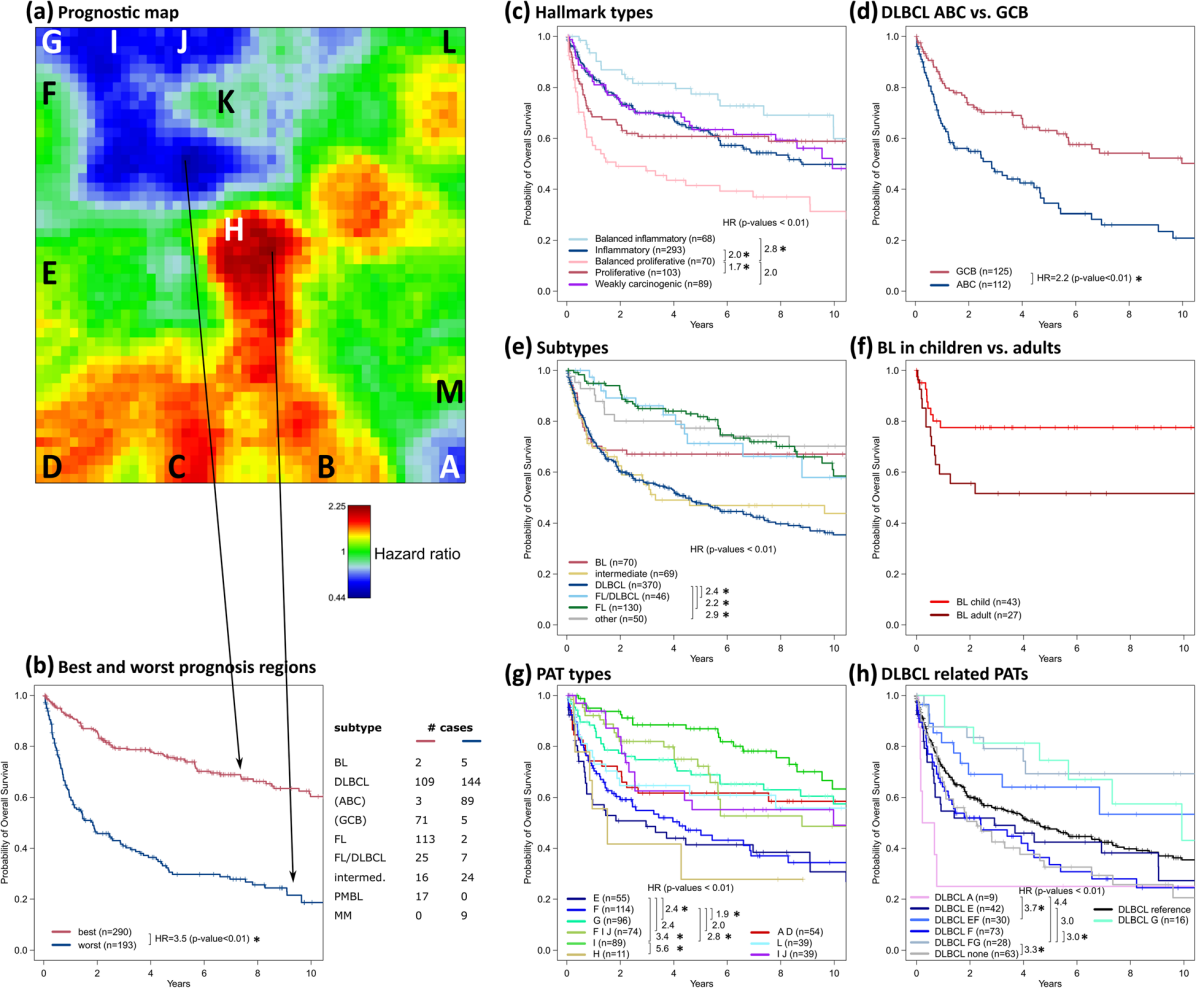
Prognostic map and overall survival (OS) curves for selected groups of tumors. The prognostic map obtained reveals regions of worse prognosis in red and of better prognosis in blue (panel **a**). The dark blue region near spots K (HR < 0.5) and the maroon region near spot H (HR > 2) associate with best and worst prognosis, respectively. The respective OS curves (**b**) resemble that of the balanced proliferative HT and of ABC-DLBCL on the one side and that of the balanced inflammatory HT and of GCB-DLBCL on the other side (panels **c** and **d**). **e**–**h** OS curves of the major subtypes (**e**) which are further stratified for children and adults for BL (**f**). OS curves of selected PATs (**g**) and of DLBCL-related PATs (**h**) associate spot combinations with prognosis. Hazard ratios (HR) are given for significantly differential curves with *p* value < 0.01 in Cox model. HRs which are still significant after adjustment for therapy are marked with an asterisk. See also Table S4 and Table S5 for HRs and *p* values of all pairwise comparisons and of the co-factors

Next, we generated a prognostic map by associating high expression levels in each of the metagenes of the SOM with the hazard ratio (HR) between the lymphoma patients expressing and not expressing this metagene (Fig. 8a). Red regions of bad prognosis include spots B–D upregulated typically in the proliferative HT and especially the balanced proliferative HT, while blue areas of better prognosis refer mainly to genes upregulated in the balanced inflammatory HT expressing spots G–J predominantly in DLBCL, FL and FL/DLBCL (compare with Fig. 7c). The overall survival (OS) curves of the HTs confirm this observation (Fig. 8c). Inflammation (and stromal) signatures in combination with healthy B cell and tonsil characteristics obviously associate with better survival, while proliferation in combination with inflammation worsens it. Regions of best and worst prognosis near spots K (HR< 0.5) and H (HR > 2), respectively, indeed collect genes that upregulate in the two balanced HTs (compare with Fig. 7c). Interestingly, the respective OS curves (Fig. 8b) resemble that of GCB- and ABC-DLBCL (Fig. 8d), whose portraits show over-expression in the regions of low and high HR around spots K and H, respectively (see Fig. 4). These regions were assigned to B cell development and B cell receptor pathway activity (spot K) and maturation into plasma cells (spot H) harboring the genes BCL6 and PRDM1, respectively, with key roles in lymphomagenesis [72, 73]. The composition of cases from both regions indeed reveals a higher prevalence of ABC-DLBCL and MM with plasma cell characteristics for worse prognosis and of GCB-DLBCL, FL, FL/DLBCL and PMBCL for better prognosis (Fig. 8b). Stratification of the HR map regarding the lymphoma subtypes reveals common prognostic patterns as evident in the overall HR map (Additional file 1: Figure S15).

Figure 8e shows OS curves of the major lymphoma subtypes. That of FL tumors reflects the indolent but in most instances incurable character of this disease [74]. In contrast, about 25% of the BL cases die within 2 years after diagnosis, but afterward, the survival curve indicates good prognosis for the survivors. Stratification with respect to age provides a significantly better long-term prognosis for children (*p* = 0.02, HR = 0.4) in terms of the plateau level (Fig. 8f). Stratification of the OS curves for the PATs further diversifies prognosis (Fig. 8g). The DLBCL cases split into PATs with better (‘G’, ‘E F’ and ‘F G’; HR = 0.5–0.7; HRs refer to all other DLBCL) and worse (‘F’, ‘E’, ‘A’ and ‘none’; HR = 1.3–2.2) prognosis (Fig. 8h, Additional file 1: Table S4). Hence, spot F collecting genes involved in inflammatory response seems to play an ambivalent role, depending if activation is in concert with, e.g., module ‘E’ or sole of spot ‘F’. Sole expression of spot A in double-hit DLBCL drastically worsens prognosis (Fig. 8h). Poor prognosis of DLBCL associates with expression of spot D (see, e.g. the portraits of PATs ‘A’ and ‘E’ in Fig. 5a, and Fig. 8a). These PATs are in correspondence with a recently identified molecular high-grade (MHG) group of DLBC which is characterized by a proliferative and BL-like phenotype which enriches double-hit lymphomas [75].

Overall, it should be taken into account that due to the retrospective nature of the study, patients had been treated with various chemotherapy regimens including rituximab in only a part of cases. Nevertheless, the prognostic map links gene signatures of poor and good prognosis with underlying molecular functions. ABC- and GCB-like transcriptional characteristics associate with worst and best prognosis of DLBCL, respectively. Stratification with respect to PATs associates spot-related molecular programs with the aggressiveness of the disease. GIF animations visualize the mutual relatedness of the PAT- and HT-related SOM portraits (Additional files 5 and 6).

### Phenotype similarity and tumor development

**Fig. 9:**
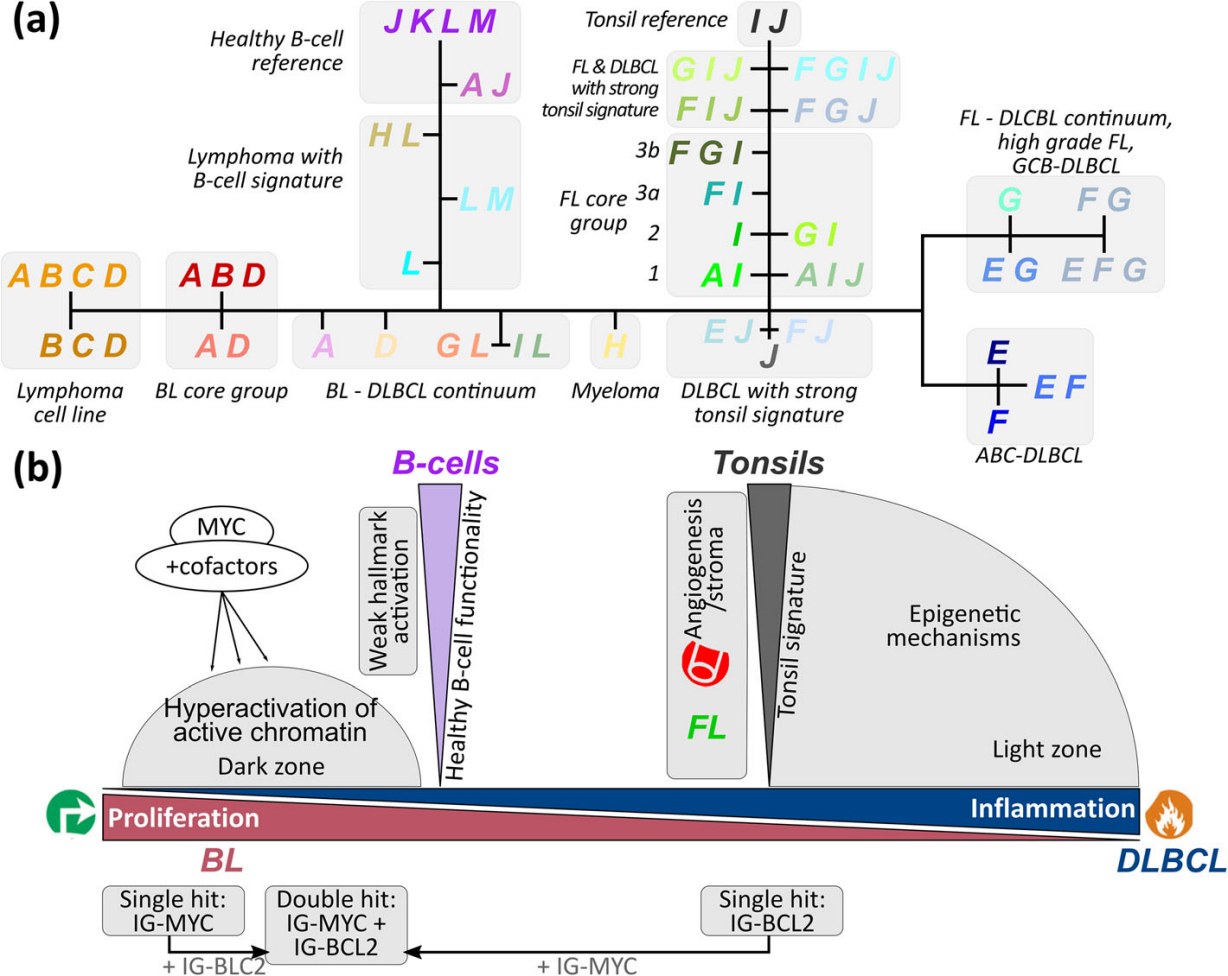
The lymphoma phenotype similarity tree. **a** The PAT-level tree visualizes the similarity relations between the core regions of the subtypes, the mutual transition ranges and their relation to the controls. **b** Different regions of the landscape associate with different B cell-related expression signatures and changing hallmark characteristics

SOM portraying further enabled us to establish phenotypic trees of mutual relatedness on three levels of resolution, namely for individual sample portraits, mean subtypes and mean PAT portraits, respectively (Additional file 1: Figure S16). The intermediate PAT level provides the most informative tree structure showing one backbone with two major side branches and well-resolved PAT leaves (Fig. 9). The horizontal backbone describes a series of PATs referring predominantly to lymphomas of the BL, intermediate and DLBCL subtypes (from the left to the right). It is characterized by antagonistic alterations of a dark zone (DZ)-like proliferative signature and more light zone (LZ)-like and inflammatory signatures.

The left vertical side branch collects mainly DLBCL cases with weak carcinogenic hallmark characteristics and also multiple myeloma showing both similarities of their transcriptomes with healthy B cells. The second side branch on the right contains mainly FL with increasing resemblance with tonsil’s expression signature. On average, the grading of FL increases towards the end of this branch due to gained transcriptional specifics of FL in terms of PATs expressing spot I with increasing grade. On the other hand, FL/DLBCL (FL3b) accumulate along the main backbone as mixed G-type PATs expressing also spot F as the main hallmark of DLBCL which manifests transformation of FL into DLBCL. Hence, FL development splits into two different paths, either reflecting an increasing level of the FL characteristics (spot I) or an increasing contribution of the DLBCL-specific spot-signature F in FL/DLBCL in correspondence with [76]. The expression landscape illustrates also another path of FL progression which is associated with the appearance of a second chromosomal translocation gained in addition to the primary t(14;18) hit [69]. Here, we exemplarily considered a secondary t(8;14) IG-MYC translocation, which induces a jump-like change of the expression phenotype by activating module A. It leads to PATs closely resembling that of IG-MYC-positive single-hit lymphoma with an activated proliferative cellular program (Fig. 9b). Overall, the phenotypic tree establishes similarity relations between the transcriptomes of the major lymphoma subtypes in terms of common and different transcriptional programs; it identifies a distinct branch of lymphomas expressing similarities with healthy B cells, and it reveals possible progression paths, e.g. of FL with increasing grade and composite lymphomas such as DLBCL/FL.

## Discussion

We presented a transcriptome map of B cell lymphoma which provides a holistic view on their expression landscape, the heterogeneity of activated gene-regulatory programs and their association with different lymphoma subtypes. The novelty hereby is that the map considers the whole range of variation of mature B cell lymphoma including a series of subtypes and healthy cell references and that it enables modularization of the landscape into expression states, their functional interpretation and visualization in terms of portraits of the different lymphoma strata and individual cases. These states can be grouped into five hallmark types on the coarsest level of stratification with proliferation, inflammation and stroma/angiogenesis as the most relevant hallmark dimensions. Combinatorial pattern types of activated modules stratify the lymphomas with higher resolution. The lymphoma map allows the evaluation of the transcriptome landscape which combines different aspects: (i) subtype-specific over- and under-expression; (ii) biological functions of the related expression modules; (iii) mutations of key genes according to their location in the map and (iv) survival hazard ratios and regions of better and of worse prognosis. Mapping of previous subtyping schemes enables the mutual comparison and characterization of GC-derived B cell lymphomas, of multiple myeloma and mantle cell lymphoma and also of the reference B cells within a unique data landscape. It reflects major aspects of B cell maturation and GC biology.

Exemplarily, our analysis provided a close look on the transition range between FL and DLBCL, on DLBCL with poor prognosis showing expression patterns resembling that of BL, and particularly on ‘double-hit’ MYC and BCL2 transformed lymphomas. In these respects, the definition of clear-cut separating criteria between the different sub-entities of lymphomas is difficult to establish due to the smooth character of their expression landscape that forms rather a continuum of molecular states than distinct clusters. These transition regions have impact regarding tumor development and transformations between different subtypes.

## Conclusions

The transcriptome map of lymphomas provides a tool that aggregates, refines, interprets and visualizes previous lymphoma data to provide a reference system in current and future studies. Particularly, it provides a reference landscape which can be utilized to map sets of signature genes and classifiers obtained in new and independent studies for comparison with the MMML cases and strata presented here, and for judging their impact in terms of function and prognosis. It considers the whole spectrum of cases in the MMML cohort thus representing an overview map. Zoom-in maps with enhanced resolution can be generated for more detailed molecular pictures of subsets of cases as demonstrated here for B cells, lymphoma cell lines and BL, and previously for DLBCL and BL [33] and in the context of human tissues [23]. Our analyses demonstrated that consideration of a wide collection of different subtypes into a joint landscape extends the state space of expression phenotypes covered in the map with sufficient resolution and allows for their interpretation in a common context. The map offers the option of extension by adding new cases from other lymphoma studies to further widen the transcriptional landscape and/or to classify and to interpret them according to the classification schemes presented. Tools such as an interactive ‘oposSOM-browser’ are presently under development for potential use in lymphoma diagnostics and molecular interpretation of gene expression patterns. Finally, our multivariate PAT concept provides a nosology scheme for describing heterogeneity also of other cancer types with high granularity.

## Additional files

Additional file 1: Supplementary text containing **Figures S1–S19** and **Tables S1–S5.** (PDF 6310 kb)

Additional file 2: Table of samples, providing histopathological and molecular subtypes. (XLSX 41 kb)

Additional file 3: Complete gallery of all 936 sample expression portraits. (PDF 14216 kb)

Additional file 4: Lists of genes for each of the spot modules. (XLSX 256 kb)

Additional file 5: Animated expression portraits of the PATs together with survival curves. (GIF 556 kb)

Additional file 6: Animated expression portraits of the HTs together with survival curves. (GIF 249 kb)

## Abbreviations

ABC: Lymphoma of the activated B cell type
BL: Burkitt’s lymphoma
DHL: Double-hit lymphoma
DLBCL: Diffuse large B cell lymphoma
DZ: Dark zone of germinal center
FL: Follicular lymphoma with t(14;18) translocation (BCL2-positive FL)
GC: Germinal center
GCB: Lymphoma of the germinal center B cell type
GSZ: Gene set enrichment *Z*-score as introduced by [38]
GWAS: Genome-wide association study
HiGA-Pro: High gene activity, proliferative phenotype as defined by [10]
HiGA-Sir: High gene activity, stroma and immune response phenotype as defined by [10]
IG-MYC: Tumor-biopsy specimens in which MYC was fused to IGH, IGK or IGL
IHC: Immunohistochemical
LoGA: Low gene activity phenotype as defined by [10]
LZ: Light zone of germinal center
mBL: Molecular Burkitt’s lymphoma subtype according to Hummel et al. [7]
MM: Multiple myelomas
MMML: Molecular Mechanisms of Malignant Lymphoma
non-IG-MYC: Lymphomas with MYC breakpoints without fusion of MYC to an IG locus
non-mBL: Non-molecular Burkitt’s lymphoma according to Hummel et al. [7]
PAP: Pathway activation pattern as defined in [8]
PAT: Pattern types defined in this study
SOM: Self-organizing map
