# Supplementary material for: A modular transcriptome map of mature B cell lymphomas
Source: Genome Med. 2019 Apr 30;11:27. doi: 10.1186/s13073-019-0637-7 (PMC6492344; doi:10.1186/s13073-019-0637-7)
Supplement: Supplementary file 1 — Supplementary text containing Figures S1–S19 and Tables S1–S5. (PDF 6310 kb) [file 13073_2019_637_MOESM1_ESM.pdf]

Supplementary Text

A modular transcriptome map of B-cell lymphomas

Henry Loeffler-Wirth#, Markus Kreuz, Lydia Hopp, Arsen Arakelyan, Andrea Haake, Sergio B. Cogliatti, Alfred C. Feller, Martin-Leo Hansmann, Dido Lenze, Peter Möller, Hans Konrad Müller-Hermelink, Erik Fortenbacher, Edith Willscher, German Ott, Andreas Rosenwald, Christiane Pott, Carsten Schwaenen, Heiko Trautmann, Swen Wessendorf, Harald Stein, Monika Szczepanowski, Lorenz Trümper, Michael Hummel, Wolfram Klapper, Reiner Siebert+, Markus Loeffler+, Hans Binder+# for the German Cancer Aid consortium Molecular Mechanisms for Malignant Lymphoma (MMML)

# corresponding authors
+ shared senior authorship

1 Full list of members of the MMML consortium 2
2 Detailed description of the methods 3
Preprocessing of microarray data 3
SOM expression portraying 4
Sample diversity analysis 4
Detection of expression modules 4
Gene set enrichment analysis 5
Reference samples: Lymphoma cell lines, B-cells and tonsils 5
3 Supplementary results 6
Supporting maps characterize the SOM metagene space: Population, variance and significance maps 6
Portraying of lymphoma strata and cell types 7
Portraying of double hit lymphomas and recurrent FL cases 10
Expression profiles of the spot modules 12
Association between PATs and lymphoma subtypes 13
Functional profiling using gene set-maps and -profiles 14
Genomic DLBCL classification (Schmitz et al. 2018, and Chapuy et al. 2018) 19
Pathway signal flow analyses 21
Mapping of GWAS gene loci associated with lymphoma susceptibility 23
Cancer hallmark types 23
Prognostic maps of the subtypes 26
Phylogenetic trees with sample, subtype and PAT resolution 28
Zoom-in SOM analysis of lymphoma cell lines and of B-cells 29
Zoom-in SOM analysis of Burkitt's Lymphomas 33
4 Tables 35
Stratification of the MMML cohort 35
Phenotypic and molecular characterization of the lymphoma subtypes 38
Meta gene sets characterizing hallmarks of cancer 39
Survival curve comparisons 40
5 References 46

1 Full list of members of the MMML consortium

Pathology group and analytes preparation: Thomas F.E. Barth [1], Heinz-Wolfram Bernd [2], Sergio B. Cogliatti [3], Alfred C. Feller [2], Martin-Leo Hansmann [4], Michael Hummel [5], Wolfram Klapper [6], Dido Lenze [5], Peter Möller [1], Hans-Konrad Müller-Hermelink [7], German Ott [7], Andreas Rosenwald [7], Harald Stein [5], Monika Szczepanowski [6], Hans-Heinrich Wacker [6].
Genetics group: Thomas F.E. Barth [1], Petra Behrmann [8], Peter Daniel [10], Judith Dierlammm [8], Eugenia Haralambieva [7], Lana Harder [11], Paul-Martin Holterhus [12], Ralf Küppers [13], Dieter Kube [13], Peter Lichter [14], Jose I. Martín-Subero [11], Peter Möller [1], Eva M. Murga-Peñas [9], German Ott [7], Christiane Pott [16], Armin Pscherer [15], Andreas Rosenwald [7], Carsten Schwaenen [17], Reiner Siebert [11], Heiko Trautmann [16], Martina Vockerodt [18], Swen Wessendorf [17].
Bioinformatics group: Stefan Bentink [19], Hilmar Berger [20], Dirk Hasenclever [20], Markus Kreuz [20], Markus Loeffler [20], Maciej Rosolowski [20], Rainer Spang [19].
Project coordination: Benjamin Stürzenhofecker [14], Lorenz Trümper [14], Maren Wehner [14].
Steering committee: Markus Loeffler [19], Reiner Siebert [11], Harald Stein [5], Lorenz Trümper [14].

1 Institute of Pathology, University Hospital of Ulm, Ulm, Germany;
2 Institute of Pathology, University Hospital Schleswig-Holstein Campus Lübeck, Lübeck, Germany;
3 Institute of Pathology, Kantonsspital St. Gallen, St.Gallen, Switzerland;
4 Institute of Pathology, University Hospital of Frankfurt, Frankfurt, Germany;
5 Institute of Pathology, Campus Benjamin Franklin, Charité–Universitätsmedizin Berlin, Berlin, Germany;
6 Institute of Hematopathology, University Hospital Schleswig-Holstein Campus Kiel/ Christian-Albrechts University Kiel, Kiel, Germany;
7 Institute of Pathology, University of Würzburg, Würzburg, Germany;
8 Cytogenetic and Molecular Diagnostics, Internal Medicine III, University Hospital of Ulm, Ulm, Germany;
9 University Medical Center Hamburg-Eppendorf, Hamburg, Germany;
10 Department of Hematology, Oncology and Tumor Immunology, University Medical Center Charité, Berlin, Germany;
11 Institute of Human Genetics, University Hospital Schleswig-Holstein Campus Kiel/Christian-Albrechts University Kiel, Kiel, Germany;
12 Division of Pediatric Endocrinology and Diabetes, Department of Pediatrics, University Hospital Schleswig-Holstein Campus Kiel / Christian-Albrechts University Kiel, Kiel, Germany;
13 Institute for Cell Biology (Tumor Research), University of Duisburg-Essen, Essen, Germany;
14 Department of Hematology and Oncology, Georg-August University of Göttingen, Göttingen, Germany;
15 German Cancer Research Center (DKFZ), Heidelberg, Germany;
16 Second Medical Department, University Hospital Schleswig-Holstein Campus Kiel/ Christian-Albrechts University Kiel, Kiel, Germany;
17 Cytogenetic and Molecular Diagnostics, Internal Medicine III, University Hospital of Ulm, Ulm, Germany;
18 Department of Pediatrics I, Georg-August University of Göttingen, Göttingen, Germany;
19 Institute of Functional Genomics, University of Regensburg, Regensburg, Germany;
20 Institute for Medical Informatics, Statistics and Epidemiology, University of Leipzig, Leipzig, Germany.

2 Detailed description of the methods

Preprocessing of microarray data

Firstly, raw probe intensity values of the Affymetrix HG-U133A arrays were calibrated and summarized into one expression value per probe set using the hook method [1, 2]. Secondly, the expression values of each gene were transformed into log10-scale and quantile-normalized conforming the distributions of expression values into one common distribution [3] This step corrects for possible outlier samples, batch effects and for sample- and transcript-specific background in cancer data [4, 5] (Figure S 1). Then, the expression values of each gene were translated into fold change units by centering them with respect to each gene's mean expression value averaged over the samples collective studied.

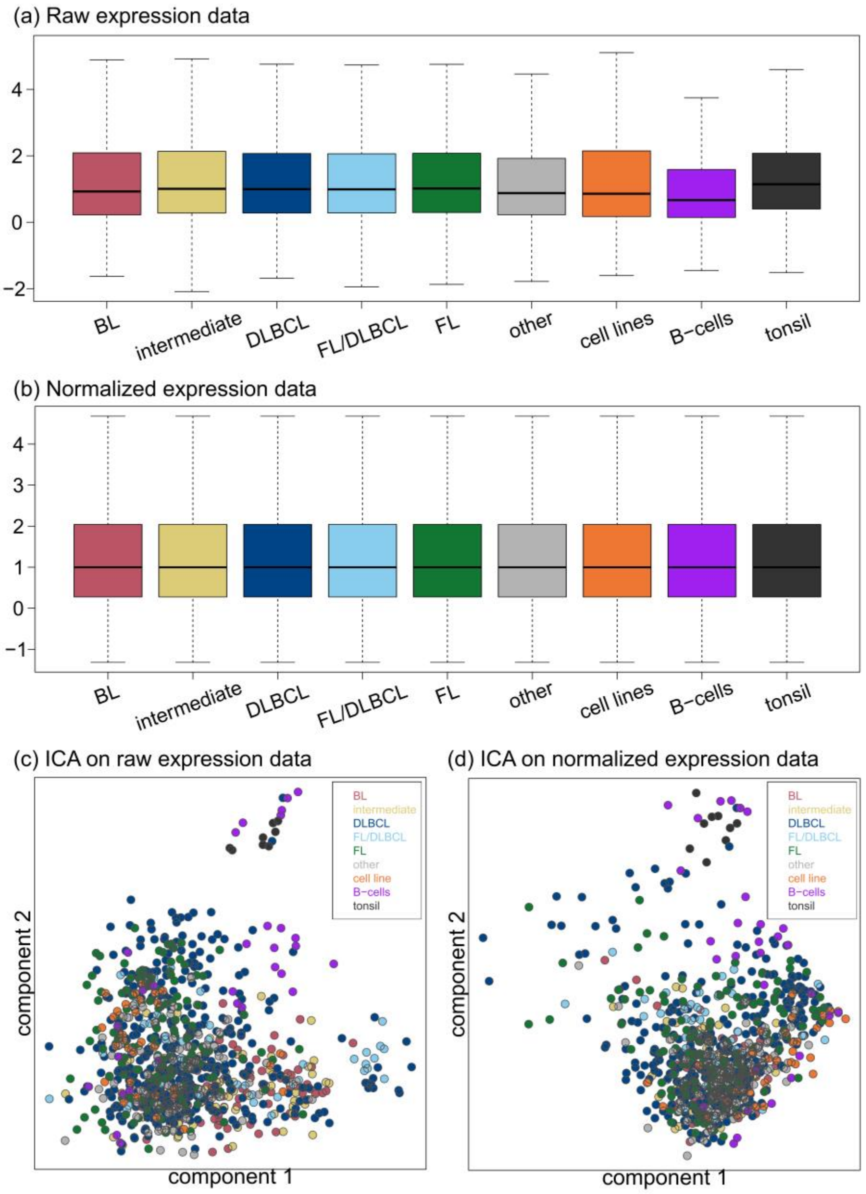

Figure S 1: Normalization of the expression data: Boxplots represent all raw expression values of the samples stratified according to the sample classes (panel a). After quantile normalization, the samples' values follow one common distribution, correcting for systematic biases and outlying samples (b). Comparison of independent component analysis (ICA) on raw (c) and quantile normalized expression data (d) reveals very similar scatter of the samples from different classes.

SOM expression portraying

Preprocessing provides a matrix of expression values of dimension $N \times M$ where N denotes the number of genes measured and M the number of cases in the study. Throughout the paper, we will address a row of this matrix as ‘expression profile’ of the respective gene, and the columns as ‘expression landscape’ or ‘state’ of the respective sample. The expression values are clustered using self-organizing map (SOM) machine learning [6]. It translates the expression data matrix into a $K \times M$ metagene data matrix of reduced dimensionality ($K << N$, K is the number of so-called metagenes). This reduces the dimensionality of the data by about one order of magnitude from N = 22,283 probe sets to K = 2,500 metagenes. The metagene expression data are visualized in a sample-specific fashion by arranging the metagenes in a two-dimensional grid of quadratic size ($K = 50 \times 50$) and by appropriately color coding their expression values. The mosaic images obtained serve as fingerprint portraits of the transcriptional activity of each sample. Importantly, the expression portraits of different samples can be directly compared each with another because a pattern at a certain position of the images refers to the same genes in all portraits. Subtype-specific mean portraits are generated by averaging the metagene landscapes of all cases belonging to one subtype. These subtype-specific expression landscapes level out the heterogeneity of the individual cases.

Sample diversity analysis

Diversity analysis aims at characterizing the heterogeneity of the tumors by means of similarity patterns of their expression landscapes. As standard, we use a graph representation called correlation network which intuitively displays multivariate relations between the expression landscapes based on their mutual Pearson’s correlation coefficients (see refs. [4, 5, 7] for details). As a complementary method, we visualize the similarity relations between the samples as a radial dendrogram by applying the neighbor-joining algorithm [8] (R-package ‘ape’ [9]). The distance between pairs of samples in the obtained tree scales with the Euclidian distance between their expression landscapes.

Importantly, both methods were applied to the metagene data and not to the original ‘single gene data’ to compare the expression landscapes as characterized by the SOM portraits. The use of metagene data improves representativeness and resolution of the methods as shown previously [7, 10–12].

Detection of expression modules

SOM machine learning arranges metagenes with similar expression profiles in neighboring positions in the two-dimensional grid, whereas dissimilar ones are located more distantly. In consequence, the expression portraits display smooth color textures with disjoint red and blue spot-like regions. They represent clusters of correlated metagenes over- and under-expressed in the respective samples, respectively. ‘Over-expression’ spot-clusters were determined in two steps: Firstly, metagenes exceeding a given expression threshold (here: 90% of maximum expression) were selected in each of the subtype-specific mean expression portraits. Then, spot module clusters were extracted as joint areas of over-expressed metagenes. Theses spot clusters can be interpreted as functional modules of co-regulated genes [10, 13].

The over-expression criterion chosen ensures the selection of a sufficient number of candidate genes per module on the one hand and a reasonable number of relevant spot modules on the other hand [4, 5, 10, 13, 14]. Note also, that the algorithm is robust with respect to moderate modifications of the selection criteria. It is also intuitive because it collects potential marker genes with mutually correlated expression profiles *and* strong over-expression in, at minimum, one of the subtypes. Importantly, our method extracts the spot modules in an unsupervised

fashion because their number is not predetermined and hence they can be interpreted as an intrinsic measure of the complexity of expression patterns observed in the data.
All spot modules detected were transferred into the expression landscape overview map shown in the main article. It represents an integrative master map which visualizes the global spot patterns observed. The underlying 'spot expression matrix' of dimension $S \times M$ (S: number of spot modules identified; M: number of samples) contains the expression profiles of the modules which were calculated as average over the single genes' profiles mapped in the respective spot.

Gene set enrichment analysis

The co-expressed genes of each spot module are assumed to be functionally related according to the 'guilt-by-association' principle [15]. We applied gene set enrichment analysis to the lists of single genes in each of the modules to discover their functional context.
In total, we included 9,769 pre-defined gene sets from the following categories in our analysis: (i) GO-sets (3,325 gene sets) were derived from the Gene Ontology (GO) annotation database [16] using the 'biomaRt' interface [17]. They subdivided into the GO-terms 'biological process' (2,226 sets), 'molecular function' (642 sets) and 'cellular component' (457 sets). (ii) Pathway-related sets were included using the Biocarta (217 sets), KEGG (186 sets) and Reactome (674 sets) databases. (iii) Literature-curated gene sets were extracted from publications describing, for example, disease signatures and biomarkers ('literature sets', 5,343 sets). (iv) Tissue-specific gene sets (24 sets) were derived from a gene expression study on healthy human tissues [13].
The degree of enrichment of a selected gene set among the genes included in a given spot module is estimated using right-tailed Fisher's exact test as described previously [18, 19]. In addition, we used the gene set enrichment z-score (GSZ) to evaluate the profile of the respective gene sets across the study (see [13, 20] for details). Systematic changes of GSZ values between the subtypes indicate potential functional relevance of the respective gene set with respect to the molecular classes studied.
The GSZ profiles are complemented by gene set (population) maps which visualize the distribution of the member genes of a selected set within the SOM grid. The gene set population map enables to identify highly populated regions of the map and to compare their positions with that of the over-expression spots detected. Accumulation of genes of a set within a spot reflects functional associations between the gene set and the spot module.

Reference samples: Lymphoma cell lines, B-cells and tonsils

Our study considers reference expression data obtained from normal (healthy) B-cells, lymphoma cell lines and tonsils. They comprise lymphoma cell line types (32 samples), B-cell types (30 samples) and tonsils (10 samples) as specified in Table S1. Tonsillar tissue samples were prepared as whole tissue RNA extracts. Naïve, post GC, and memory B-cells were isolated from peripheral blood samples of healthy individuals. GC B-cells were isolated from suspended tonsillar cells. For isolation of the B-cell subsets, FACS sorting employing antibodies against CD19 and IgD (naïve B-cells), CD20 and CD38 (tonsillar GC B-cells), and CD19 and CD27 (post GC memory B-cells) was used. Lymphoma cell lines were described in [21].

3 Supplementary results

Supporting maps characterize the SOM metagene space: Population, variance and significance maps

We implemented a series of so-called supporting maps and profiles, which provide additional information about the SOM metagene space to assess the population of metagenes with genes, the variability of metagene profiles, the significance of differential expression with respect to the mean expression value of each gene and the correlation between metagene and single gene expression (see [7, 10]). The population map visualizes the number of single genes mapped to each metagene (Figure S 2a). It reveals that the single genes heterogeneously distribute among the map forming densely populated regions along the edges (red and brown colors) separated by empty and lowly populated areas (dark and light blue).
The variance map in Figure S 2b illustrates the variability of the metagene expression profiles. Most variable metagenes are found in the corners and along the edges of the SOM (red and brown) while invariant genes accumulate in the center of the map (blue regions). The significance map color codes the significance of differential expression with respect to the mean expression for each of the metagenes using an adjusted t-test [13] (Figure S 2c). Significance and variability are correlated properties because highly variable genes mostly show a more significant degree differential expression and also stronger coefficients of correlation between the metagene and single gene profiles (Figure S 2d).
The supporting maps clearly illustrate that the spot-modules of co-regulated genes selected for further analysis mostly agree with highly-populated areas of the map. They also refer to highly variant and thus significantly differentially expressed genes (compare the supporting maps with the over-expression overview map shown in the main article).

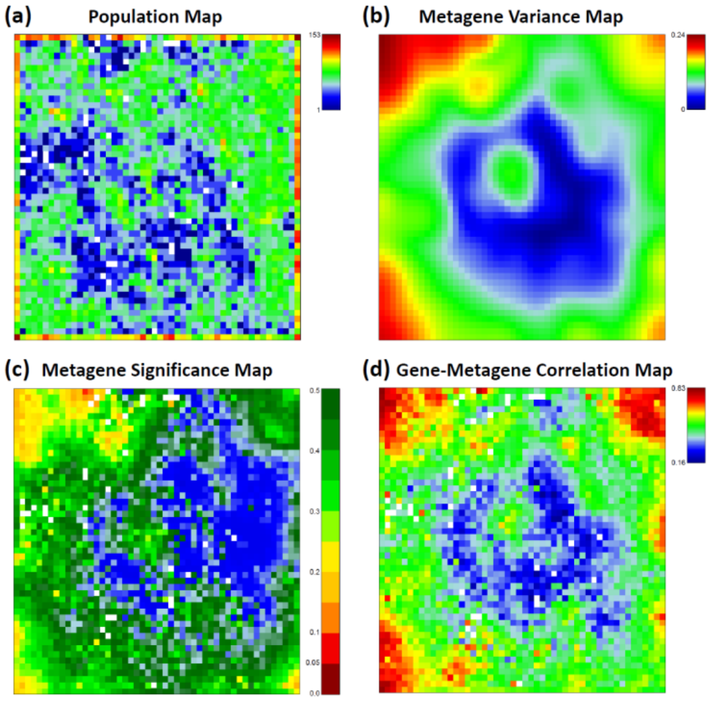

Figure S 2: Supporting maps: (a) The population map illustrates the number of single genes mapped to each metagene. (b) The variance map shows the variance of the metagene expression profiles. (c) The significance map illustrates the mean p-value for differential expression averaged over all metagene member genes. (d) The gene-metagene correlation map shows the mean correlation between the metagene expression profile and the member genes' profiles.

Portraying of lymphoma strata and cell types

We generated mean expression portraits averaged over the histological subgroups in each sample class under study (Figure S 3). Intermediate lymphoma split into two complementary subgroups with almost orthogonal module activation patterns (strong over-expression of module L and D, respectively). BL-like intermediate lymphomas show a complex expression pattern with multiple over-expressed spots, while D is specific for the other intermediate lymphoma. Note that the mBL signature genes as defined in [22] accumulate strongly in spot B as described below. Activation of B hence supports classification of the BL-like intermediate lymphoma.

DLBCL split into GCB and ABC classes, which both show F and, partly, G as marker spots. Additionally, spot module E is specific for ABC DLBCL and will be evaluated below. We further subdivided DLBCL cases according to their morphology determined by haemato-pathological review as described in [23–25]. The resulting subgroup portraits of anaplastic and centroblastic DLBCL are nearly identical showing the DLBCL specific spots F and G. The immunoblastic and plasmoblastic cases reveal a slightly altered gene activity pattern with more activated spot modules, among them also F and G.

Cases assigned as FL mostly feature a t(14,18) translocation (BCL2 break), however portraits and activated modules are virtually independent of presence or absence of this aberration: The resulting portraits consistently show the FL-specific spot I, in case of BCL2-break FL in combination with increased gene activities in the areas of spots K and A also found in B-cells and BL, respectively. With increasing grade, the FL specific spot I partly transforms into spot F paralleled by decreasing gene activities in the regions of spots B, C and K. Grade 3b FL, which constitute the class FL/DLBCL, show an intermediate pattern combining FL and DLBCL specific spots I and F. These changes are compatible with the continuous transformation from FL into DLBCL.

Few lymphomas in the data could not be assigned to one of the former subtypes. Multiple myelomas consistently show a specific spot module H located in the center of the map. The H spot harbors the PRDM1 gene which becomes activated at late stages of plasma-cell maturation paralleled by deactivation of spot K including MHC II genes in agreement with [26].

PMBL and IRF4-break positive lymphoma strongly resemble GCB and ABC DLBCL, respectively. mnBLL-11q lymphomas resemble BL as expected, with A and D activated.

The lymphoma cell lines show very similar expression patterns (over-expression of spot modules A, B, C, and D) with additional spots selectively activated in double hit BL cell lines (M) and myeloma cell lines (H, in agreement with the myeloma samples), which confirms the respective cell of origin characteristics.

All lymphoma cell line samples show very low expression of spot modules F and G (see also the spot profiles in Figure S 5) due to the absence of inflammatory and stromal bystander cells in the cell cultures. Because of the min-max normalization of the color scale of the portraits, this low expression of modules F and G gives rise to a 'red shift' of the remaining part of the map which appears mostly colored in red. This color-shift slightly masks the underlying spot patterns in the 'red part' of the map. Nevertheless, the plasma-cell of origin characteristics of the MM-cell line portrait becomes evident by the strong expression of spot H harboring, e.g., the plasma cell marker gene PRDM1 as mentioned above. Note also that the DLBCL cell line portraits differ from the respective DLBCL tissue specimen due to the absence of inflammatory bystander cells. A more detailed evaluation using adjusted portraits and zoom-in SOM analysis reveals subtle details of the different cell lines, which allows for further characterization of their transcriptomic landscapes (see below).

The B-cell reference samples divide into groups defined by their maturation grade. The mean portrait of the B-cell class simultaneously expresses the spot modules s J (tonsil signature spot), and K, L, and M (B-cell specific). This expression pattern is virtually identical in the portraits

of pre- and post-germinal center (GC) B-cells. Interestingly, these cells under-express module D, whereas GC-B-cells over-express these genes reflecting activated proliferation in the germinal center (see below). The B-cell specific spot modules K, L, and M differ in their activity in the lymphoma subtypes: For example, K is up in FL but down in BL and DLBCL, L is up in BL-like intermediate lymphomas only, and M is strongly deactivated in DLBCL but activated on high and moderate levels in the other subtypes. Hence, modules K-M collect regulatory modes activated in B-cells and selectively deactivated in the different lymphoma subtypes.

Also the B-cell specimen show low expression of spot modules F and G (Figure S 5) due to the absence of inflammatory and stromal cells in the cell cultures, which gives rise to an analogous color shift as observed in the lymphoma cell-line portraits. Nevertheless, cell of origin characteristics of GC-B and pre-/post GC-B-cells are still evident in the different expression patterns of the proliferation module D (see above). Adjusted portrait and zoom-in analysis provide more details of the B-cell portraits studied (see below). Note that the portrait of tonsils is characterized by the strong over-expression of spot J (Figure S 5) reflecting a specific transcriptomic signature of this tissue enriched in keratin-related compounds [5]. This one-spot over-expression characteristic leads to a 'blue-shifted' coloring of the remaining part of the portrait.

In summary, stratification of the molecular subtype portraits with respect to histological and genetic diagnosis reveals subtle differences between the subclasses in accordance with the associated functional changes (e.g. transformation from FL into DLBCL with increasing grade or increased proliferative activity in GC-B-cells compared with pre- and post-GC-B cells).

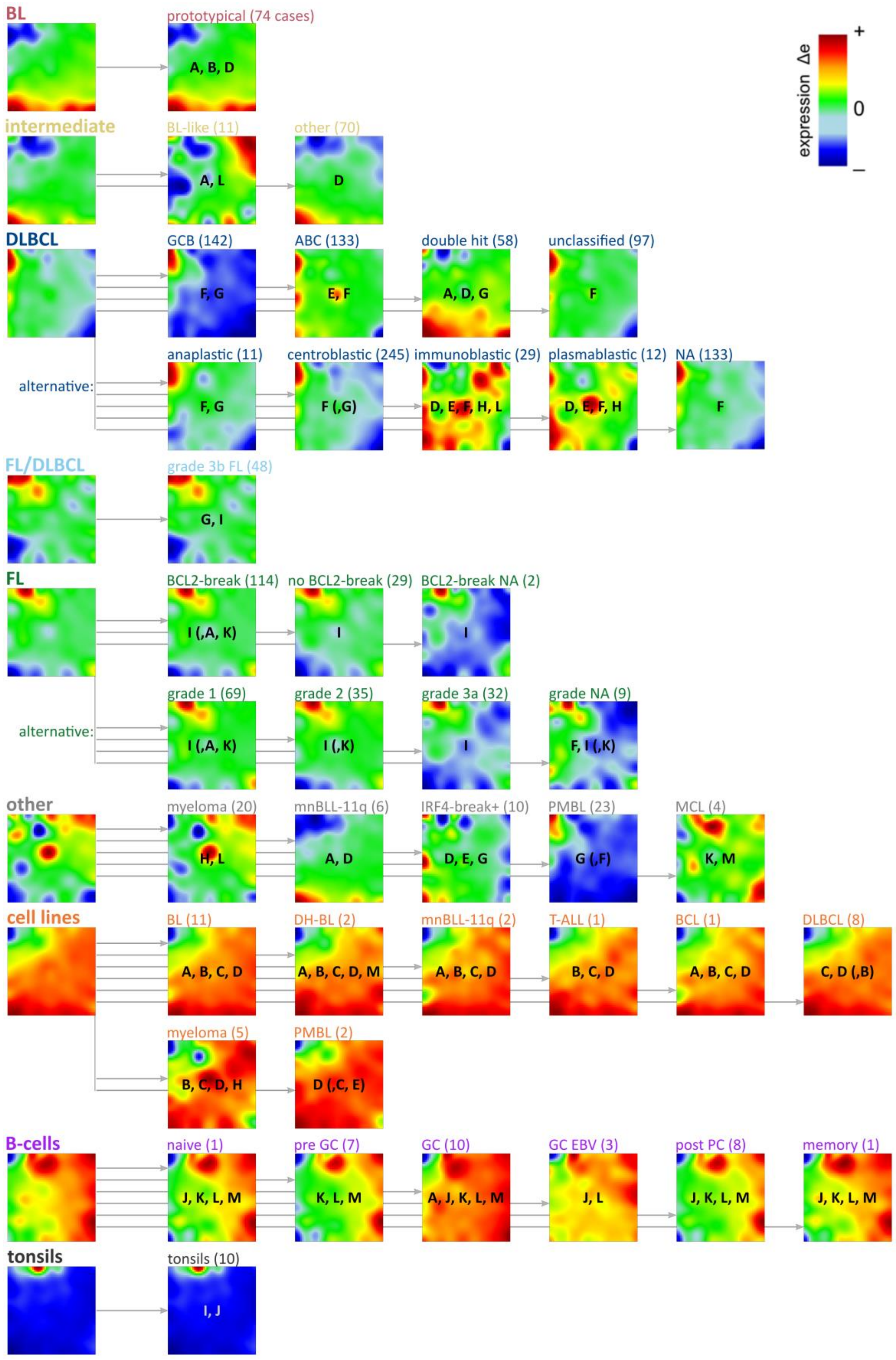

Figure S 3: Mean expression portraits of histologically defined subgroups in each class: The left portrait in each row shows the mean class portrait, which is decomposed into the corresponding histological subgroups. Recurrently activated spot modules are indicated within the portraits by capital letters.

Portraying of double hit lymphomas and recurrent FL cases

We stratified the double hit lymphomas (DHLs) in our data set to portray the specifics of their expression landscapes. In DHLs, a MYC translocation is accompanied by a second chromosomal break targeting the BCL2 or BCL6 gene. Chromosomal translocations affecting the MYC oncogene are the biological hallmark of BLs, but also occur in a subset of other mature B-cell lymphomas. Following the tumor selection strategy applied in [27], we focused on the latter tumors and excluded BLs. Firstly, we generated mean expression portraits of single hit lymphomas carrying either an IG-MYC, non-IG-MYC or IGH-BCL2 (t14,18) translocation (Figure S 4a). The latter cases are FL showing their typical expression pattern which is dominated by spot module I (compare with Figure S 3). The IG-MYC and non-IG-MYC cases are DLBCL or intermediate lymphomas expressing module D in combination with B and C typically over-expressed in BL (IG-MYC cases). DHLs carrying the BCL2 translocation and the IG-MYC or non-IG-MYC both show similar portraits with over-expressed spot A and under-expressed spot F. This spot patterns are not a combination of the patterns of the respective single hit lymphomas. Instead, they represent a novel feature of these DHLs which express spot A as a hallmark. The analogous analysis of lymphomas combining the MYC- and BCL6-translocation shows, that these DHLs are characterized by expression portraits which combine the spot patterns of the respective single hit lymphomas including the ‘inflammation module’ F and the ‘proliferation module’ D (Figure S 4b). Next, we analyzed two IGH-BCL2/-MYC signatures in our data (Figure S 4c) [27, 28]. These signatures are very similar in upregulating genes which accumulate in spot A and which show high expression also in BL and BL-resembling intermediate lymphomas, but not in the majority of DLBCL. Accordingly, BCL2-DHLs downregulate genes in the spots E, F, H and, partly, K in both signatures. Note that the signatures of Aukema et al. and of Ennishi et al. do not contain overlapping genes. Their joint accumulation in the same spot patterns, however, reflects their almost identical expression profiles and demonstrates the power of gene-mapping for comparison of gene sets.

Finally, we selected four paired lymphoma samples of primary lymphomas diagnosed as FL grade II or III carrying the IGH-BCL2 translocation, and relapsed tumors from the same patients (Figure S 4d). Two of the secondary tumors were hit by IG-MYC or non-IG-MYC during relapse while the two others are not. The DHL cases show the hallmark spot A while the MYC-negative single hit recurrent lymphoma show clear indications of transformation into DLBCL or intermediate lymphomas. The latter example illustrates the capabilities of SOM portraying for studying changes in the expression patterns for individual cases. Taken together, SOM portraying comprehensively characterizes expression patterns before and after acquiring a second hit in DHL combining MYC with BCL2 or BCL6 translocations for matched and non-matched tumor pairings.

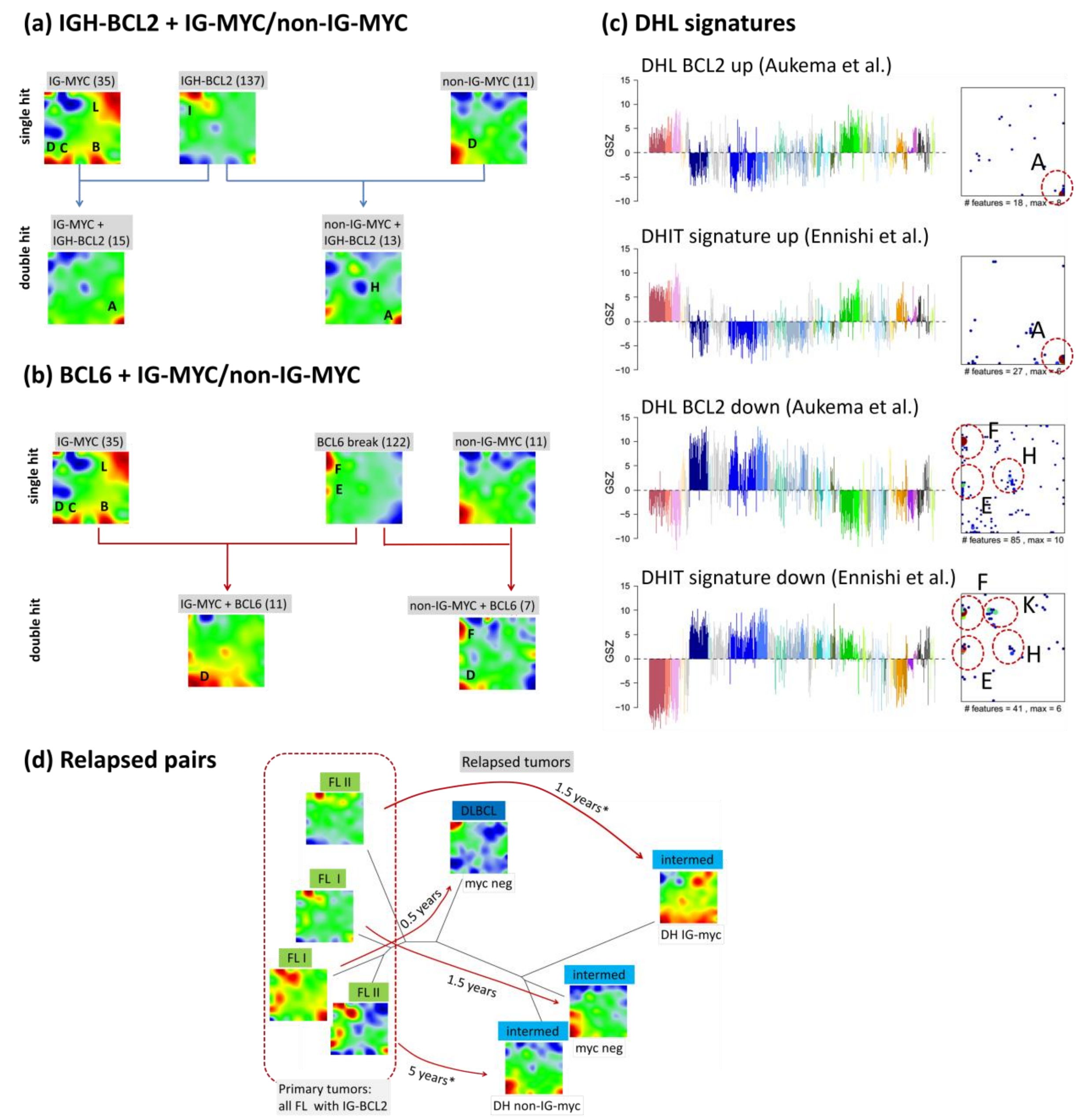

Figure S 4: SOM portraying of double-hit lymphomas and of recurrent FL: (a) Mean expression portraits of single hit IG-MYC DLBCLs, non-IG-MYC DLBCLs and IGH-BCL2 FLs, and of DHL showing combinations of the aberrations. Notably, spot A appears in the DHL as a new feature not observed in the single hit lymphomas. (b) Portraits of IG-MYC and non-IG-MYC DLBCL (as in part a) and of BCL6-break DLBCL single hit lymphomas, and of DHL showing combinations of the aberrations. The DHL virtually combine the expression features of the respective single hit lymphoma types. (c) Expression profiles and gene maps of DHL signatures taken from [27, 28]. The two different DHL up and DHL down signatures closely resemble each other as indicated by the accumulation of the gene set genes in the same spot regions (see red circles and spot letters) and by the nearly identical profiles. (d) The portraits of four FL IGH-BCL2 cases (grade I or II) and of the paired tumors after recurrence (see arrows) are analyzed in terms of phylogenetic-tree similarity comparison. Two recurrent cases showed a second hit (IGH-MYC and non-IGH-MYC). The portraits of the recurrent DHL show upregulated spots A and D as the typical features of DHL (see part a). The two other recurrent lymphomas reflect the transformation of FL into either DLBCL or into intermediate lymphoma types.

Expression profiles of the spot modules

Figure S 5 shows the spot module expression profiles which are calculated as mean expression values averaged over all genes of the module for each sample studied. Most of these profiles show a subtype-specific maximum in agreement with the spot-assignments given in the main article. The samples were sorted according to their PAT-assignments which partly results in the systematic modulation of the profile amplitudes.

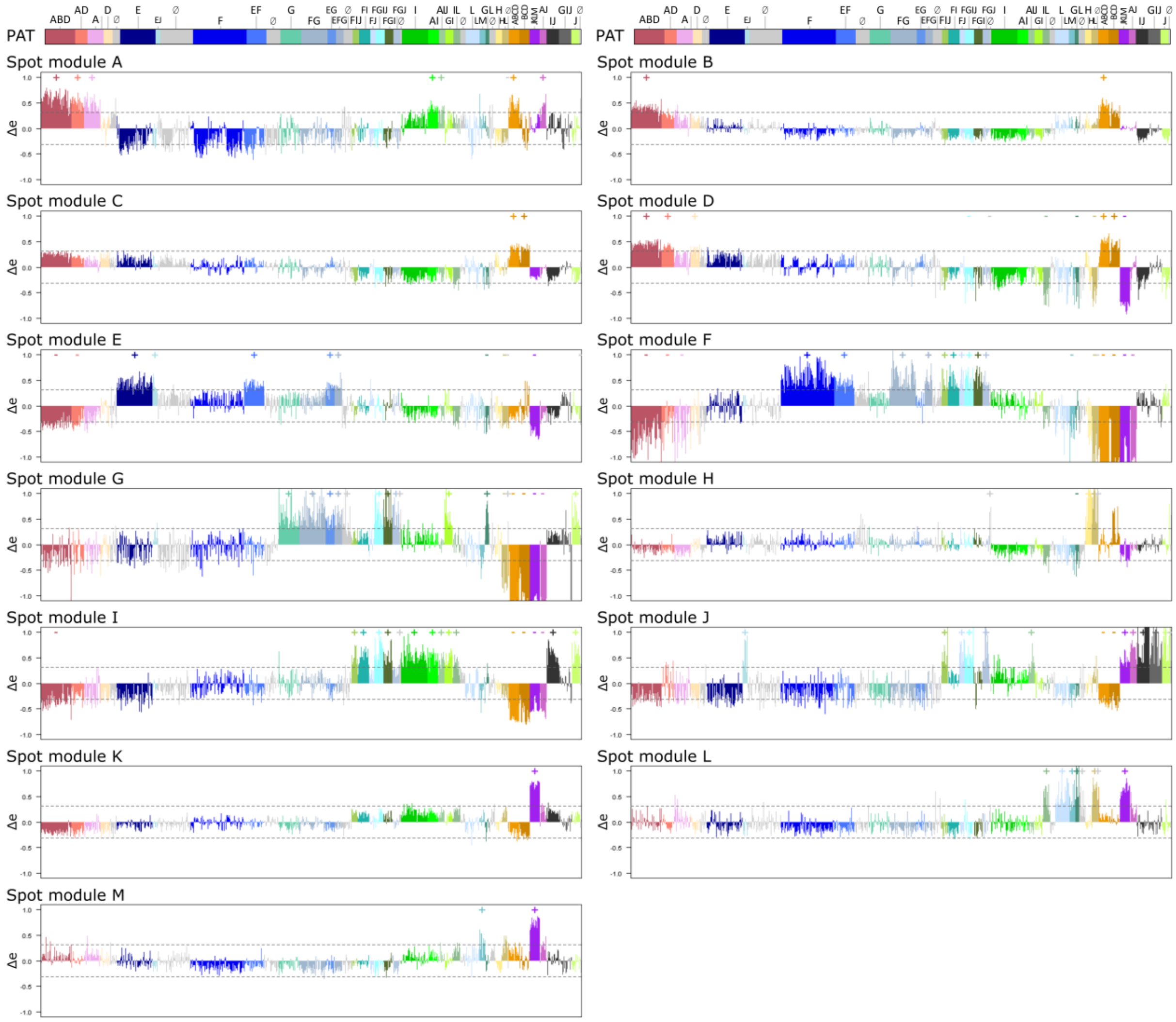

Figure S 5: Spot expression profiles across all samples: The profiles are generated as the mean expression profile averaged over all genes contained in the respective expression module. Each bar represents one sample. The signs indicate strong over- (+) and under (-) –expression according to the standard deviation threshold indicated by dashed horizontal lines.

Association between PATs and lymphoma subtypes

For association of PATs with lymphoma subtypes, we used right-tailed Fisher's exact test. The resulting p-values are given in terms of a heatmap (Figure S 6). It reveals, that BLs exclusively enrich in PATs 'A B D', 'A D', and, to a less degree, in PAT 'A'. Intermediate lymphomas associate with PAT 'D' solely. ABC DLBCLs mainly associate to E- and F-type PATs, in contrast to GCB DLBCLs, which enrich in PATs 'A' and 'F G'. FLs consistently associate with I-type PATs and also with PAT 'I J'. Interestingly, PAT 'G' only enriches in GCB DBCLs and in FL/DLBCLs, reflecting the similarity of these two subtypes. Some PATs correspond to a low number of samples only, e.g. 'A J', 'E G' or 'L M', and therefore lack of significant enrichment in the subtypes. In summary, this analysis supports our assertion that certain PATs associate consistently with certain disease types.

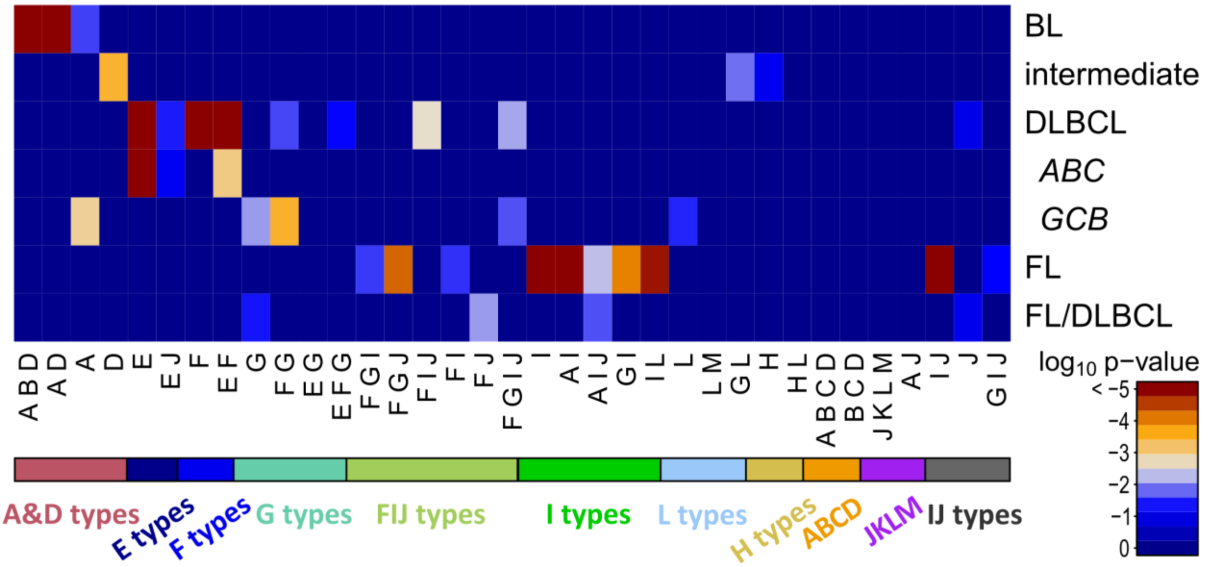

Figure S 6: Association between PATs and lymphoma subtypes was estimated in enrichment p-values (Fisher's exact test).

Functional profiling using gene set-maps and -profiles

Gene set enrichment Z- (GSZ-) profiles and population maps were generated for a series of gene sets related to specific activation in BL, DLBCL FL and cell line samples (Figure S 7), to previous lymphoma marker sets and selected pathways (Figure S 8) and to B-cell and immune system characteristics (Figure S 9). The gene set maps allow to identify regions of increased local density of genes (see dots) and to compare their localization with selected over-expression spot areas. The GSZ-profiles show subtype-specific activation and de-activation of the related cellular programs. Gene set maps and profiles thus supplement the spot-related enrichment analysis and enable a more detailed functional analysis of the map.

Proliferation-related processes such as cell cycling and DNA repair were upregulated in BL and cell line samples (Figure S 7a), whereas immune response and inflammation were activated in DLBCL and partly also FL (Figure S 7b). The population maps reveal that the member genes of the sets mostly accumulate in the regions of the respective signature spots (compare with Figure 3 in the main article). We also evaluated the mBL-vs-non-mBL differential signatures provided by Hummel et al. [22] (top panels in Figure S 7a and b). The signature genes accumulate in the spots 'B' (up in mBL) and 'F' (down) which cover only part of the gene activation patterns of lymphomas. Stromal signatures [29] upregulate mainly in FL while the signature genes accumulate in spots G and I (Figure S 8). Signature genes of B-cell activation and –proliferation accumulate in the regions of spots M, A and K, respectively (Figure S 7c). T- and B-cell receptor signaling pathways accumulate in modules I and K, respectively, showing low GSZ values in BL and high values in FL (Figure S 7d). Interestingly, PAT 'E' shows antagonistic activities of T- (down) and B- (up) cell receptor signaling thus representing a differential feature between both pathways.

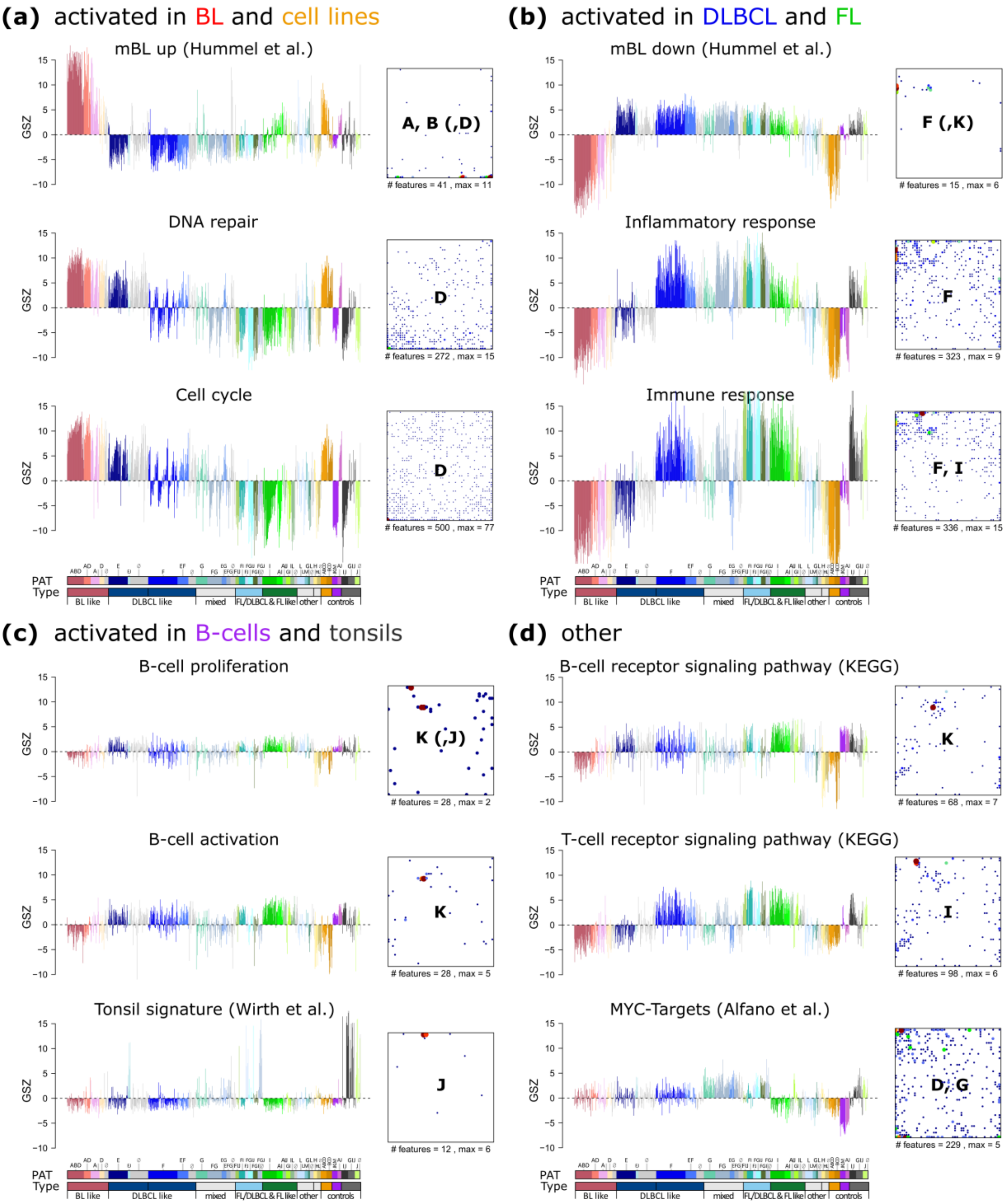

Figure S 7: GSZ-profiles and population maps: Gene sets activated in (**a**) BL and cell line samples, (**b**) in DLBCL and FL, and (**c**) in B-cells and tonsils. The bars correspond to the samples and are colored according to their PAT classification (see legend on bottom). The population maps illustrate the distribution of gene set members across the SOM landscape. Highest to lowest population is depicted as big red to small blue circles, white areas indicate areas not containing genes from the respective set.

Next we analyzed further marker sets published previously (Figure S 8): GCB-ABC-signature genes [30], immune response signatures [29, 31], targets enhanced and repressed by transcription factors [32], targets of TP53 [33], genes up- and down-regulated by PI3K [34], as well as genes involved in IL21 [35], NFκB [36], toll-like receptor signaling and MAPK pathways. Most of these gene sets reveal characteristics as described in the main article. The immune signature sets are highly activated in DLBCL (except PAT 'E') and FL subtypes and deactivated in the BL subtype. The ABC-signature is maximally expressed in PAT 'E', mainly associated with ABC DLBCL.

Additionally, we included signature gene sets into our enrichment analysis corresponding to different stages of healthy B-cells and to different components of the immune system (Figure S 9). In particular, signatures of plasmoblasts, plasma cells, mature plasma cells, and B-cells were taken from Tarte et al. [37]. Interestingly, these different B-cell signature genes accumulate in different PATs as indicated in the figure. Accumulation of plasmablast signature genes in spot D is attributed to the dominating proliferative activity of these cells. Plasma cell signature genes clearly cluster in spot H. This spot module is activated in multiple myeloma. The mature plasma cell signature genes accumulate in spots F and G, strongly over-expressed in DLBCL and also FL. The B-cell signature genes accumulate in spot K activated in the B-cells used as controls.

We included signature gene sets activated in dark (DZ) and light zone (LZ) of germinal centers taken from [38]. As expected, these sets are associated with BL (DZ), and to DLBCL and FL (LZ). Interestingly, the LZ and DZ expression signatures change in anti-concert with the signatures of CD40-targets repression and activation, respectively, which were taken from [39]. Hence, CD40 deactivation obviously associates with LZ functionality and, in turn, CD40 activation with DZ function. This observation supports the view that CD40 signaling modulates the GC reaction and the development of B-cells [39]. Note that CD40 deactivation antagonistically regulates with the LZ signature for all subtypes except healthy B-cells and parts of FL. CD40-targets decouple from the LZ/DZ-signature suggesting that FL remain 'locked' in LZ state.

Signatures of diverse components of the immune system were taken from Angelova et al.[40]: Signature genes of T-helper cells accumulate in spot I and partly F. They are consistently activated in FL (recall that module I is the FL specific module), but only in DLBCL related PATs involving module F ('F', 'E F', 'F G'). Activated CD4 and CD8, and memory CD4 signatures show very similar GSZ profiles across the samples with activation in BL and DLBCL, and deactivation in FL. Contrary, the profile of memory CD8 resembles the T-helper cells with deactivation in BL and activation in F and I related PATs of DLBCL and FL. Dendritic cells and monocytes also show a similar profile with subtle differences.

In summary, most of the signatures show PAT specific profiles, which are partly consistent with regard to the different lymphoma subtypes. Some of the PATs however, first of all 'E' and 'A' for DLBCL, show opposing characteristics compared to the other PATs of the subtype.

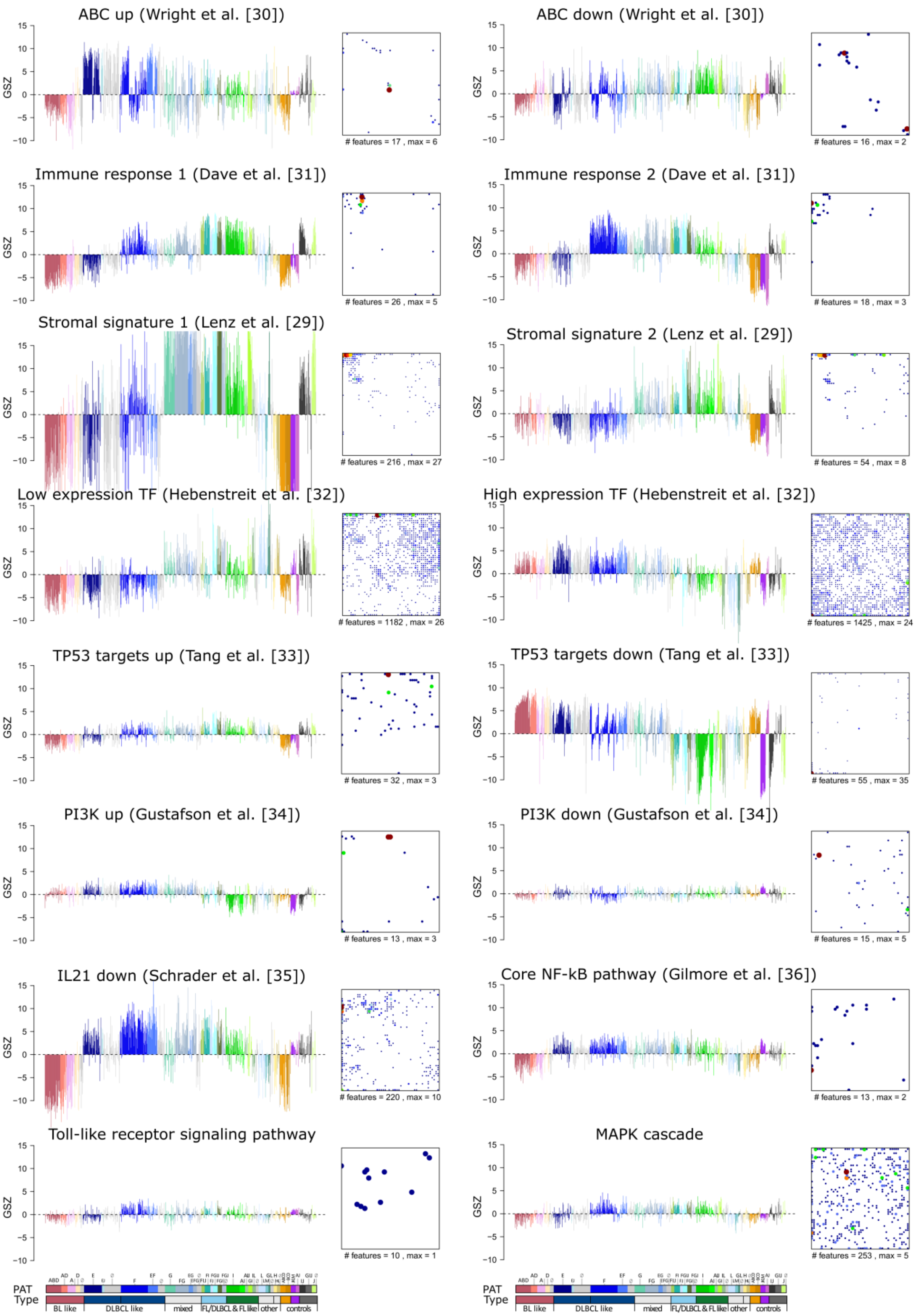

Figure S 8: Gene set profiling of selected gene sets related to previous lymphoma classifications, selected pathway activities, and biological functions. The bars in the GSZ profile plots correspond to the samples. The population maps illustrate the distribution of gene set members in the map. Highest to lowest population is depicted as big red to small blue circles. White areas indicate metagenes not containing genes from the respective set.

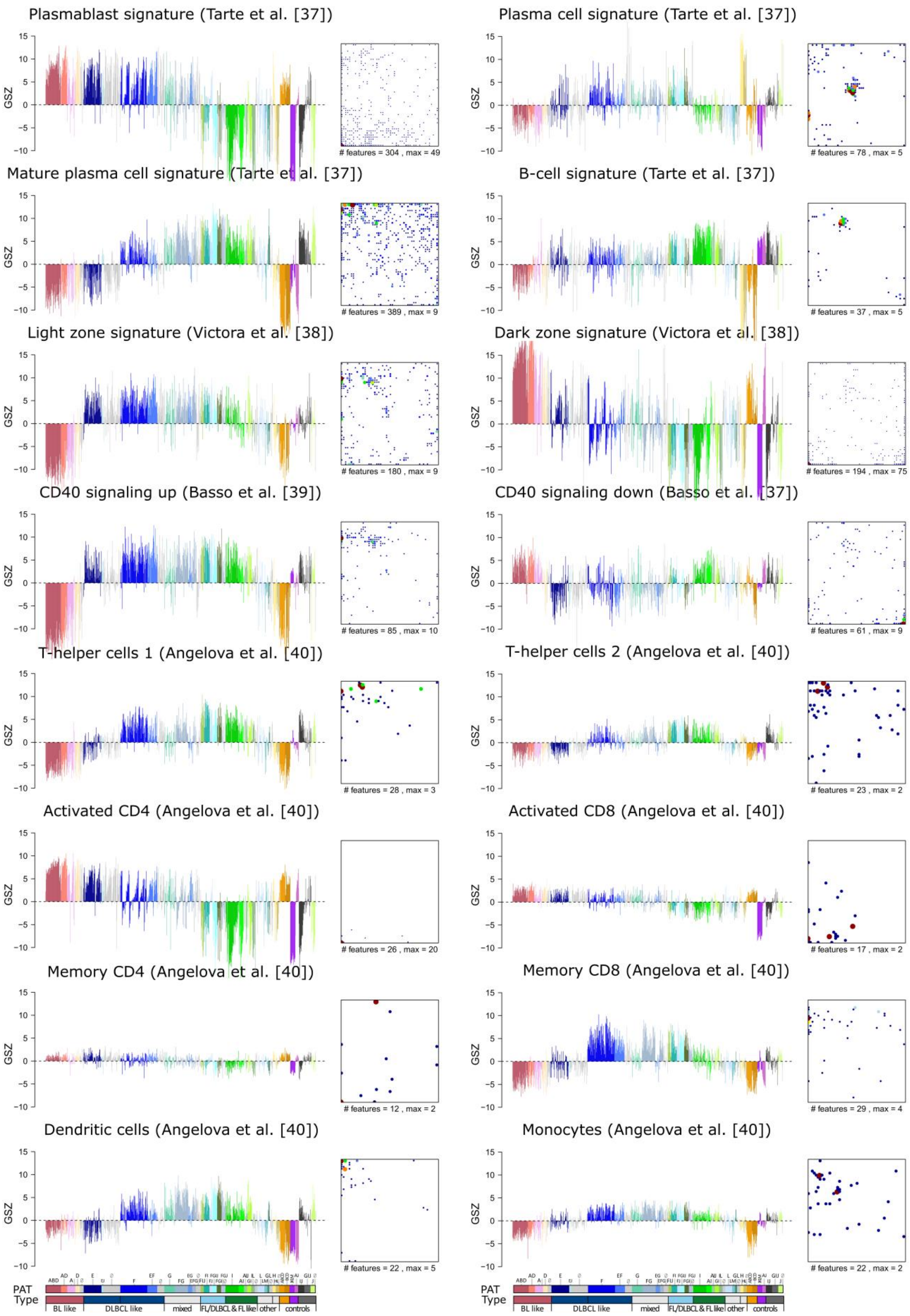

Figure S 9: Gene set profiling of signatures related to B-cell signatures, to GC-biology and to selected immune cells. See caption of Figure S 8.

Genomic DLBCL classification (Schmitz et al. 2018, and Chapuy et al. 2018)

Two recent publications presented a classification of DLBCL based on genetic data. Schmitz et al. distinguish the subtypes MCD (based on the co-occurrence of MYD88 and CD79B mutations; it collects mostly ABC DLBCL), BN2 (based on BCL6 fusions and NOTCH2 mutations), N1 (based on NOTCH1 mutations, mostly ABC DLBCL), and EZB (based on EZH2 mutations and BCL2 translocations, mostly GCB DLBCL) [41]. Chapuy et al. split DLBCL into five consensus clusters C1 – C5 assigned mostly to BCL6 structure variants and ABC-DLBCL (C1), TP53 mutations and large scale copy number aberrations (C2), BCL2 mutations and structural variants of predominantly GCB DLBCL (C3), SGK1 and HISTH1 mutations and mostly GCB DLBCL (C4) and high level gains on Chr. 18q and Chr. 3q and mutations of MYD88, PIM1 and CD79B of mostly ABC DLBCL (C5) [42].
Genes showing mutations and/or copy number aberrations in the respective subtypes were taken from the original publications, collected into subtype-related gene sets and analyzed by mean of their GSZ-profile and gene maps (Figure S 10). In addition, we counted lymphomas of our study as ‘present’ in each of the classes (GSZ exceeding one standard deviation above the mean), and then provided the composition of all present cases in terms of pie diagrams of the PAT types and hallmark types (Figure S 10).
We find that the expression profiles of the aberrant genes of the classes C2 and, to a less degree, N1 show a BL-resembling characteristics with largest amounts of PAT types ‘A and D’ (red) and ‘F’ (blue), and HTs ‘proliferative’ (red) and ‘balanced proliferative’ (rose). The C5 cluster group contains highest amounts of ‘balanced proliferative’ HT and of E-type PATs which confirms its predominant ABC DLBCL characteristics established in [41]. The MCD group and clusters C1 and C4 show largest expression for ‘F‘, ‘G’ and ‘I’-type PATs and ‘contain large amounts inflammatory HTs thus reflecting GCB DLBCL and partly FL resemblance. FL resemblance further increases for groups BN2, EZB and cluster 3 which show largest amounts of the balanced inflammatory HT. Notably, nearly all genetic classes (except N1 and C3) accumulate aberrant genes (among them BCL2, BCL6, CD79A) in and around spot K assigned to B-cell functions which reflects their impact for lymphoma biology (see also Figure 3).
Taken together, genetic classes cover the expression spectrum of HTs ranging from proliferative, balanced proliferative, inflammatory, balanced inflammatory and weakly carcinogenic HTs referring to lymphoma with BL-like, ABC- and GCB-DLBCL and FL-like resemblance, respectively. In other words, genetic characteristics associate with transcriptome strata and support the wide and continuous spectrum of DLBC expression phenotypes described here.

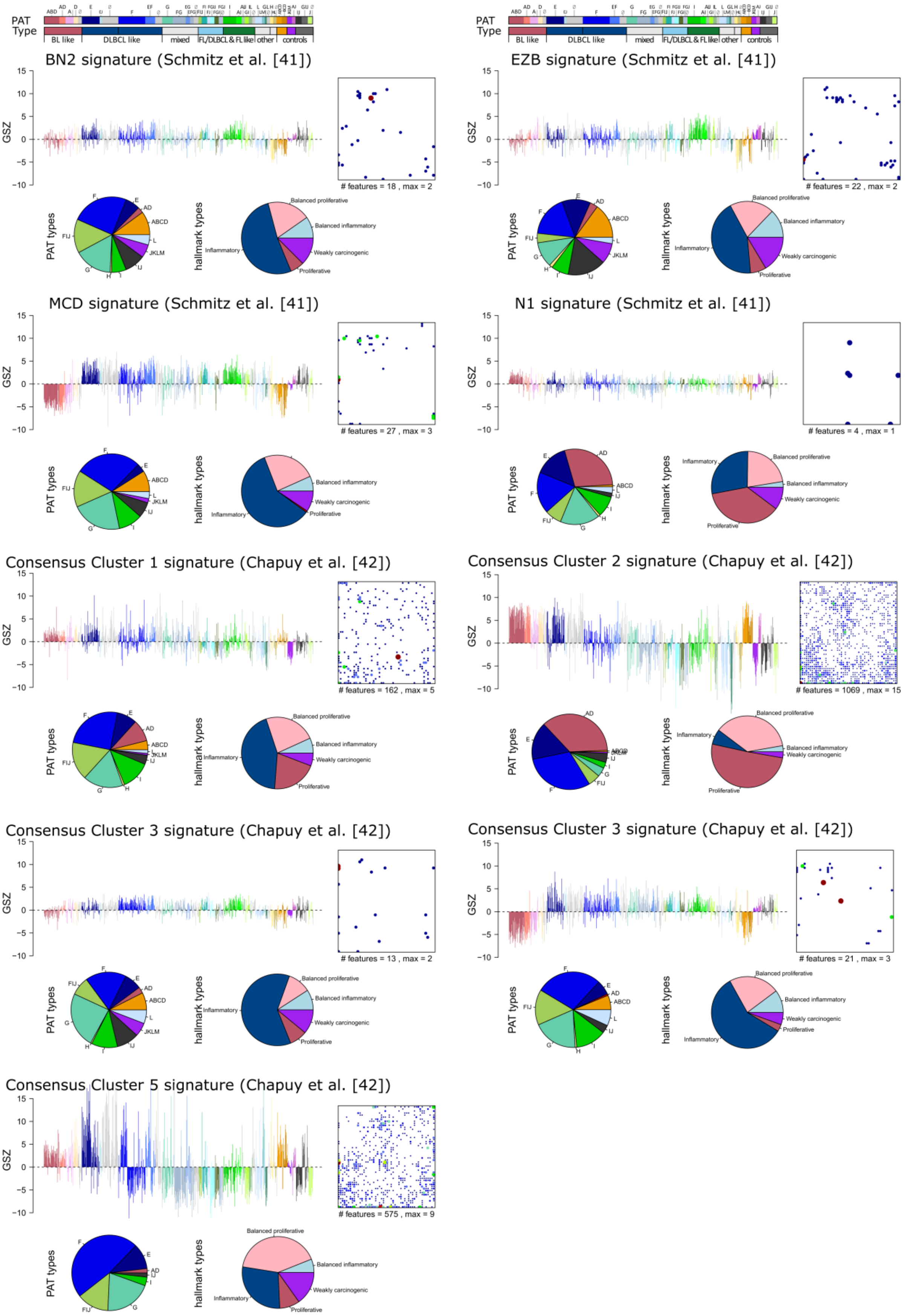

Figure S 10: Profiling of genetic groups provided by Schmitz et al. [41] and Chapuy et al. [42]. The gene sets were characterized by means of GSZ-profiles, gene maps showing the location of the set genes in the map, and percentages of PAT types and hallmark types.

Pathway signal flow analyses

The activity of selected KEGG-pathways was estimated using the pathway signal flow (PSF) method which takes into account the expression level of the genes which form the nodes in the pathway graph, and their mutual interactions [43]. The genes of both, the B-cell receptor (BCR) and of the NF-kappa-B pathways accumulate in and near spot K, which relates to B-cell function, but also in/near the spots F and I (NF-kappa-B-pathway) upregulated in DLBCL and FL, respectively (see gene maps in Figure S 11). Part of the pathway genes, however, are found also in regions of other spots reflecting different types of subtype-specific interactions (activating and inhibiting) between them. The barplot of the mean maximum pathways signal flow (PSF) value per PAT provides an overview of the pathway activities. The NF-kappa-B pathway is on higher activity level in DLBCL and FL related PATs and in healthy B-cells in contrast to BL-related PATs. The coloring of the genes in the pathways according to their PSF-value (red and blue stand for high and low, respectively) indicates PAT-specific activation patterns.

Especially the MAPK-, NF-kappa-B- and PIK3-AKT-branches of the BCR-pathway become activated in part of the DLBCL and FL compared with BL and MM (H-type PAT) because of activation of the CD79A/B and CD19 receptors. Inspection of the PSF-patterns of the NF-kappa-B pathway indicates activation of the toll-like-receptor (TLR) branch with MYD88 in DLBCL and FL compared with BL and MM. These data support the notion of 'chronic active' BCR in part of DLBCL cases due to mutation of genes in these pathways such as MYD88 and CD79A/B (gain of function) and LYN (loss of function) [44] (these genes are indicated in Figure S 11).

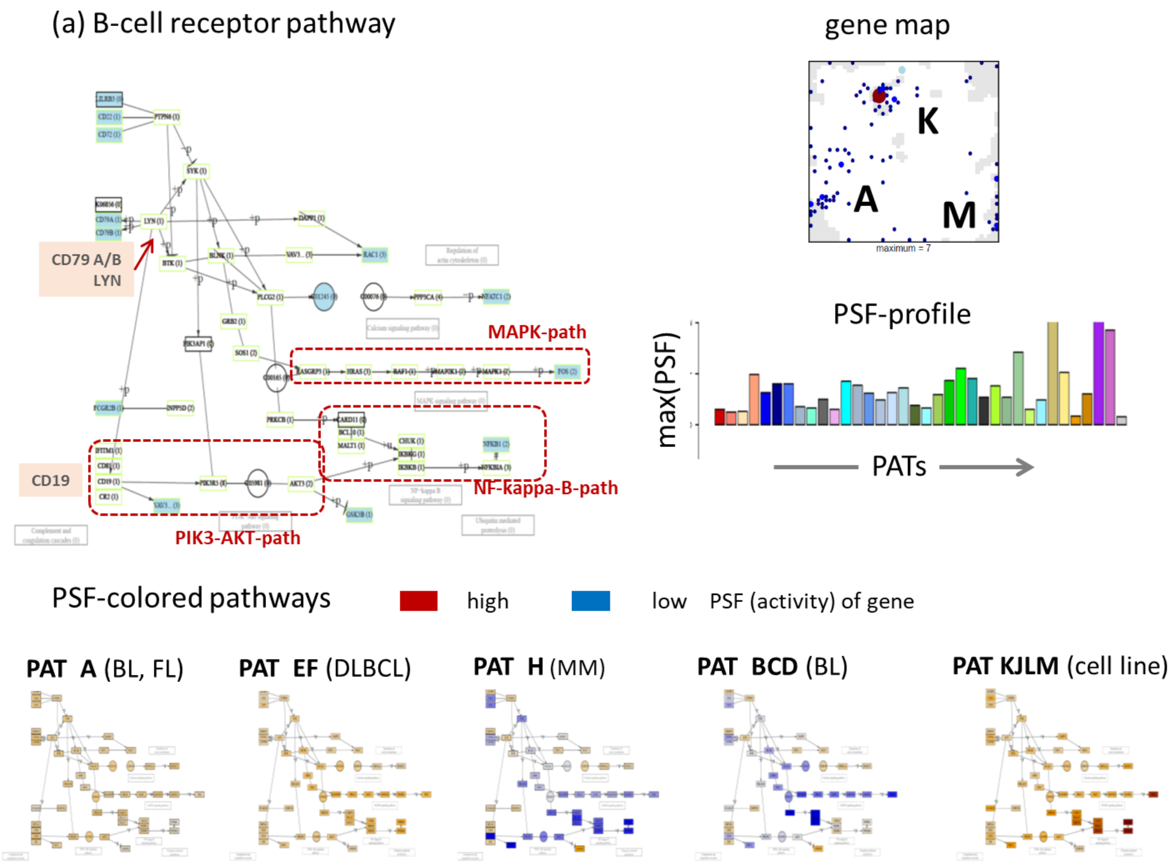

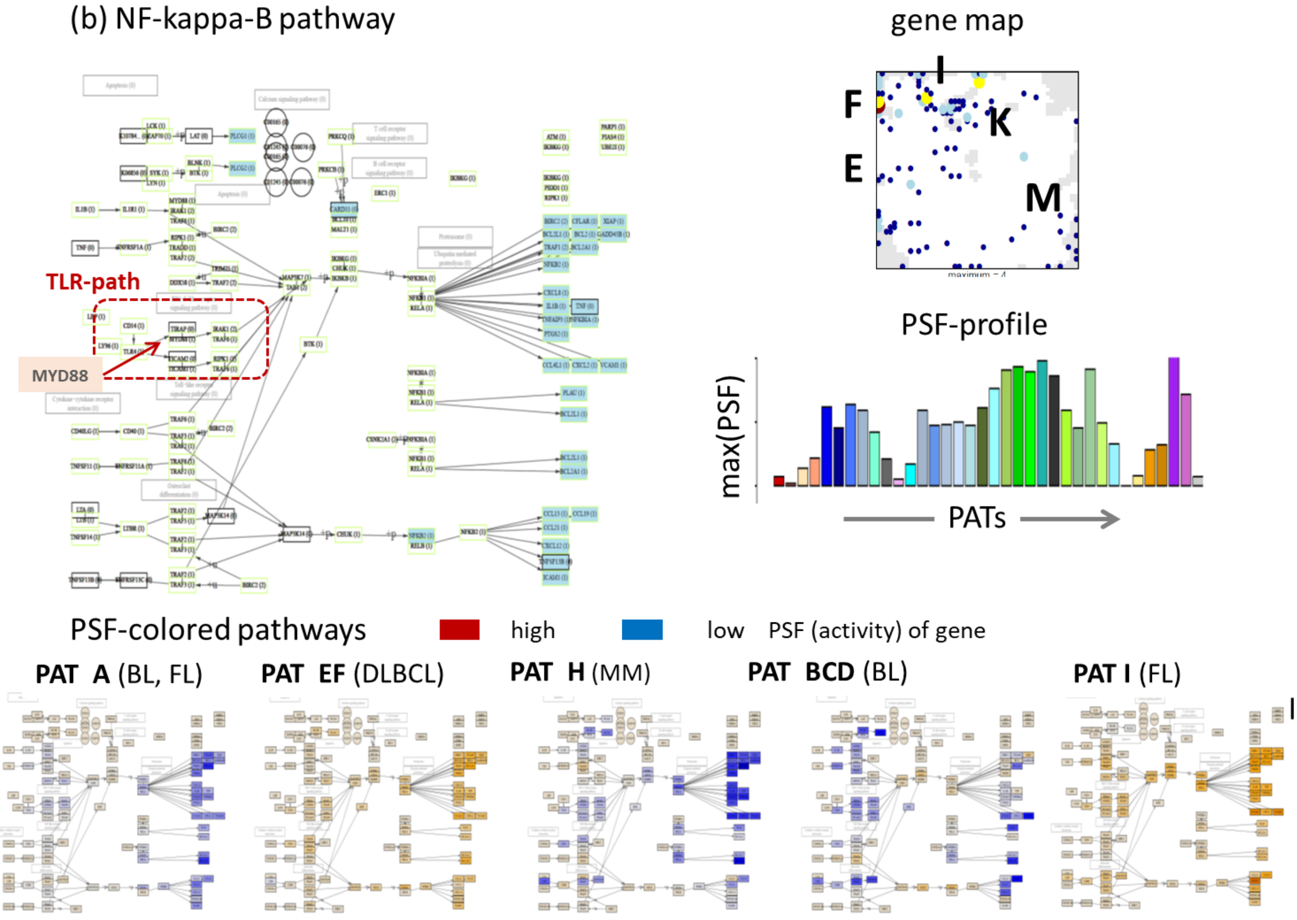

Figure S 11: Pathway signal flow (PSF) analysis of the B-cell receptor signaling pathway (BCR) (part a) and of the NF-Kappa-B- pathway (part b). The figure shows the pathway graph, the map of the pathway genes, the mean, group-averaged maximum activity of a node as boxplot in the part above. In the part below the nodes are colored according to their PSF-activity in selected PATs.

Mapping of GWAS gene loci associated with lymphoma susceptibility

We mapped selected gene loci susceptible for lymphoma into our SOM. These genes were identified recently by genome-wide association studies (GWAS) [45, 46]. These hereditary risk genes for FL and/or DLBCL accumulate near the spots K and E, and thus in similar regions as the somatic mutations associated with these lymphoma types discussed in the main article. These genetic defects in final consequence disturb the same or at least similar expression modules.

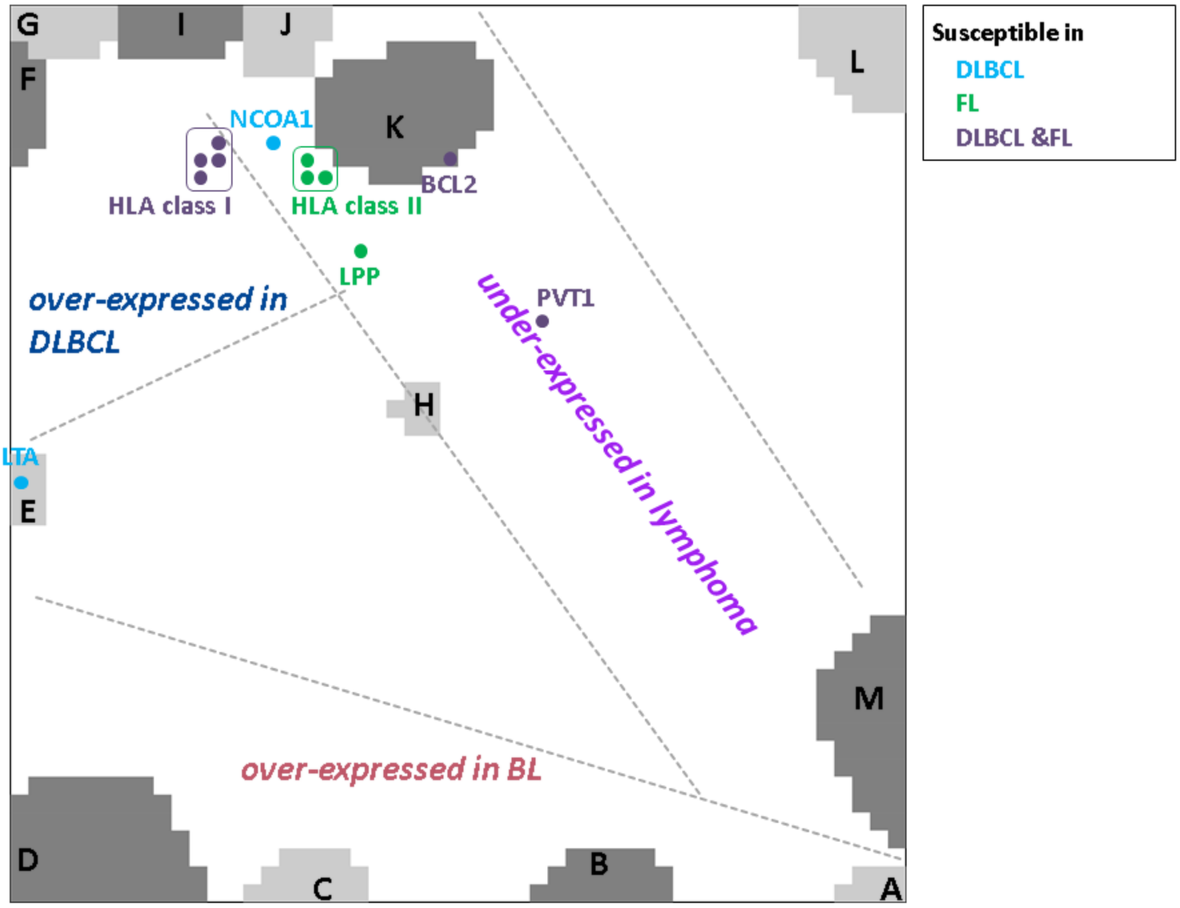

Figure S 12: Mapping of hereditable gene loci susceptible for lymphoma [45, 46].

Cancer hallmark types

We defined eight hallmark signatures using GO- and literature-gene sets (Table S 3). Figure S 13 shows their GSZ profiles in terms of subtype-related boxplots. Basically, the profiles reveal largest differential activation for the hallmarks ‘inflammation’ in DLBCL and ‘proliferation’ in BL, respectively (p-values $< 10^{-16}$ compared to healthy B-cells using the Wilcoxon rank-sum test). These results agree with well-known differences between BL and DLBCL physiology: while BLs are fast growing (hallmark ‘proliferation’) and genomically relatively stable due to their activated DNA repair mechanisms (hallmark ‘controlling genomic instability’), DLBCL were characterized by enhanced perfusion (hallmarks ‘inflammation’ and ‘angiogenesis’). The hallmark activation patterns were visualized as polar diagrams where each polar coordinate scales with the GSZ-score of one hallmark signature (Figure S 13b). The shape of these

diagrams intuitively reflects the basal character of each subtype and of each PAT in terms of cancer hallmark activities. The subtypes roughly divide into more ‘inflammatory’ (DLBCL and partly FL), and more ‘proliferative’ (BL) entities in terms of the hallmark characteristics. The large amplitude of the hallmark ‘proliferation’ in the latter tumor type is accompanied by large amplitudes of the hallmarks ‘controlling genomic instability’ and ‘metastasis and invasion’, whereas the inflammatory subtype also shows large amplitudes of the hallmark ‘angiogenesis’. Next, we analyzed the distribution of hallmark activity across the map by calculating the enrichment of hallmark set member genes in the spot modules. Figure S 13c annotates enriched hallmarks to different spot areas of the lymphoma expression landscape. As expected, hallmarks activated in BL enrich in the BL-related spot A, B and C while DLBCL and FL related hallmarks enrich in the areas of spots F, G and I. The distribution of subtype composition of the HTs is in correspondence with their spot expression patterns and the subtype distribution as discussed above (Figure S 13d).

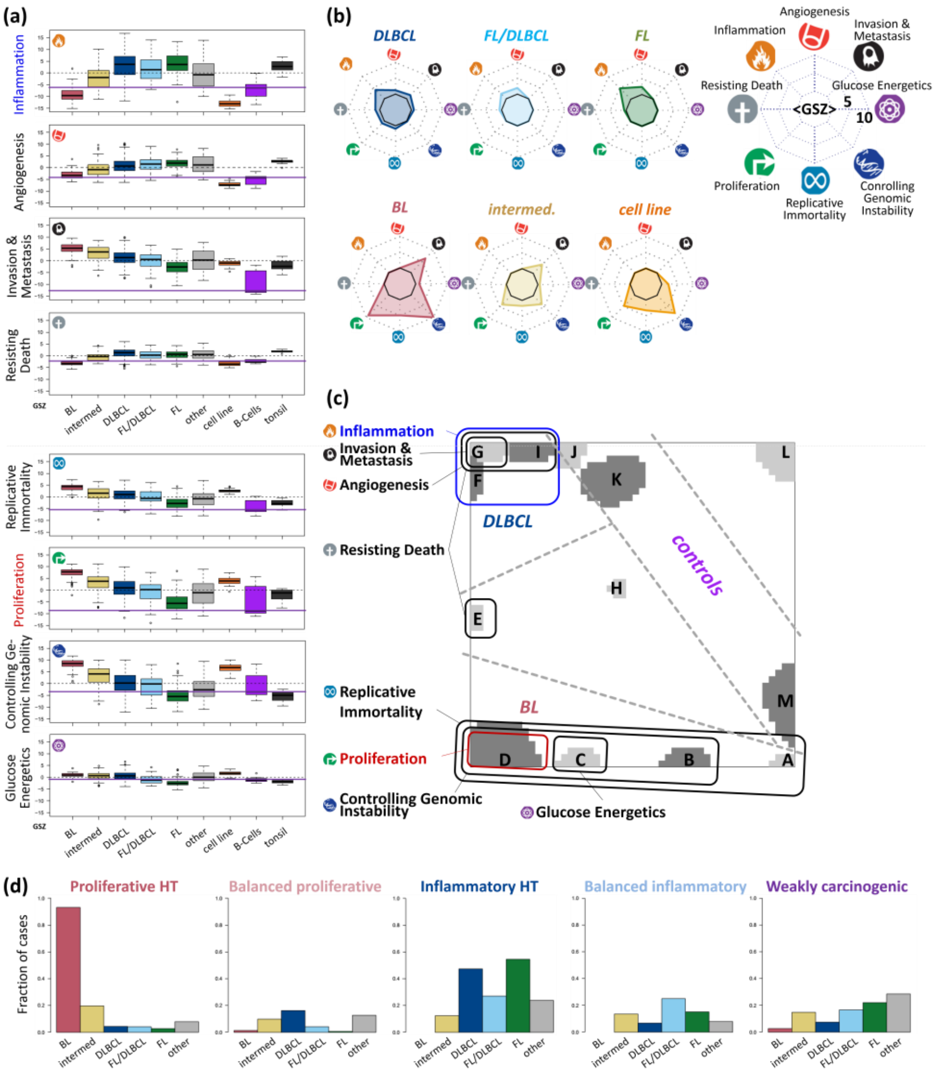

Figure S 13: Cancer hallmark characteristics of lymphomas: (a) Class specific boxplots of the GSZ-enrichment score of the cancer hallmarks meta-gene sets. Reference activity of healthy B-cells are highlighted with a horizontal line (b & d) Mean hallmark diagrams of the lymphoma subtypes: The polar coordinates are scaled in units of the GSZ-score of the different hallmark-sets as indicated in the legend. The icons are taken from ref. [47] (c) The hallmark association map relates spot modules to the cancer hallmark sets. Over-represented hallmarks are given in the figure beside the spots (p-value < 0.05 in Fisher’s exact test). (d) The subtype composition of the HTs reveals that the proliferative HT mainly contains BL, while the other HTs contain a wider distribution of subtypes in an HT-specific way.

We generated hallmark diagrams for each of the PATs (Figure S 14). One can distinguish five distinct hallmark-types: (i) The *proliferative hallmark* type with activated hallmarks proliferation, controlling genetic instability, invasion, and metastasis and, partly, regenerative immortality; (ii) The *balanced-proliferative* hallmark type with a moderate activation of the hallmarks proliferation and inflammation; (iii) The *inflammatory* type with activated hallmarks inflammation, and to a less degree proliferation and metastasis; (iv) The *balanced-inflammatory* type which partly resembles the inflammatory type however with further reduced activity of ‘proliferation’ and ‘controlling genetic instability’; and (v) the *weak hallmark* type with generally low overall hallmark activities.

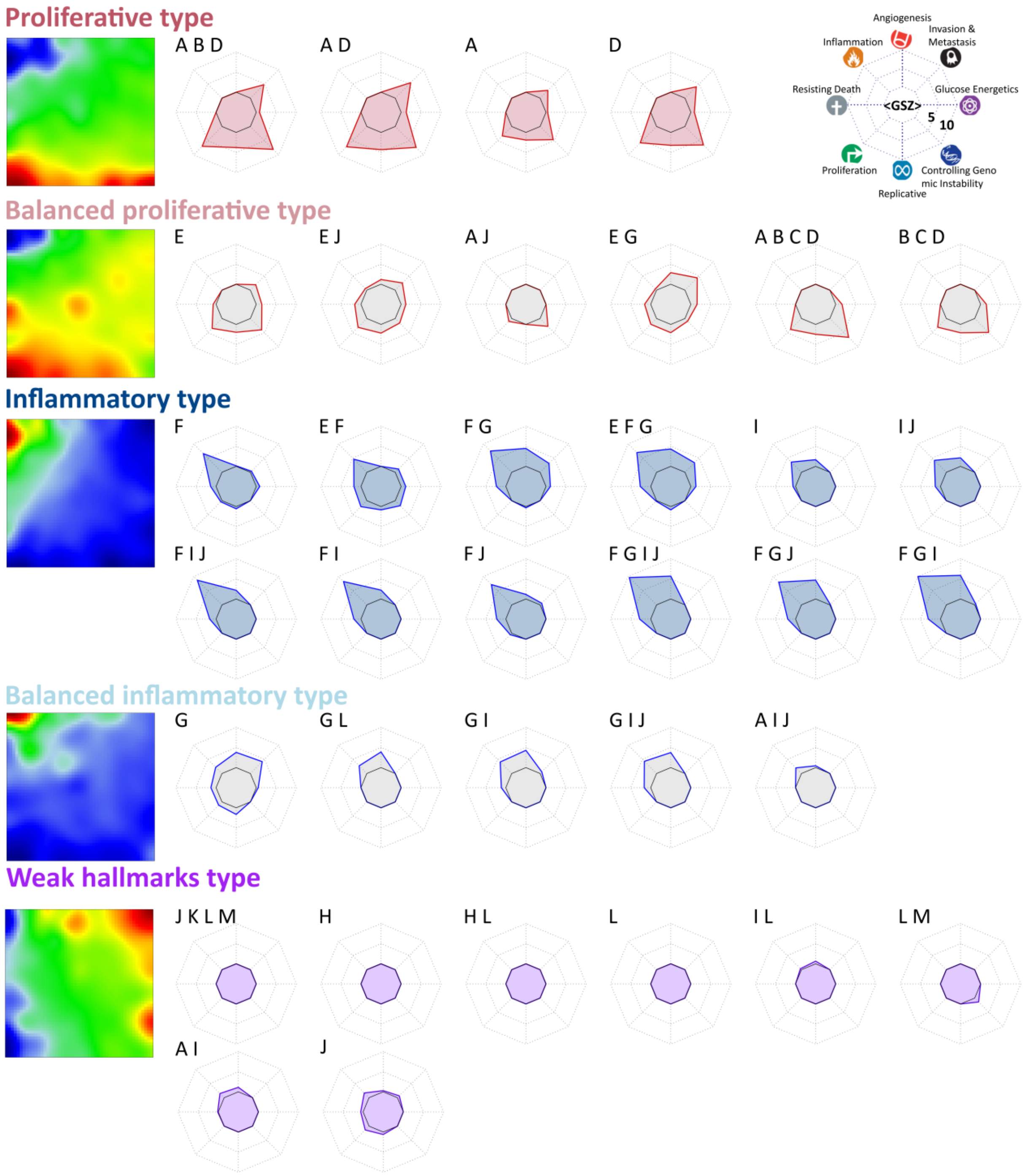

Figure S 14: Cancer hallmark characteristics of the PATs: Polar diagrams visualize activation of cancer hallmarks (see legend) for each of the PATs. They are divided into five groups of similar cancer hallmark activation, where each is characterized by the mean expression portrait of the corresponding samples.

Prognostic maps of the subtypes

In the main article, we provided a prognostic HR-map which considers all lymphoma cases of the MMML-data set (also shown in Figure S 15a for direct comparison). In addition, we calculated the subtype-specific prognostic ‘hazard ratio’ maps using the same algorithm as described in the materials and methods section. Therefore we stratified the data according to the lymphoma subtypes BL, intermediate lymphoma, DLBCL, and FL or DLBCL/FL in order to extract subtype specifics of the HR-map (Figure S 15b - e).
On the one hand side, we find differences between these subtype-specific maps, showing, for example, that DLBCLs expressing module H (hallmark of ABC-DLBCL) or module B (feature of BL-resembling DLBCLs) associate with poor prognosis, while DLBCLs expressing modules F and G (feature of GCB-DLBCLs and FL-transformed DLBCLs) associate with better prognosis. A similar pattern is observed for intermediate lymphomas where, however, the HR range (7.1 to 0.15) is much larger than for DLBCL (2.2 – 0.4). The HR-map of FL and FL/DLBCL indicates that the prognosis of these subtypes obviously worsens upon expression of proliferative spot D, while BLs with poor prognosis express genes located in and around the B-cell related spot L.
On the other hand, the different subtype specific HR-maps all reflect a common underlying pattern of genes which associate with better and worse prognosis as subsumed in the overall HR-map. Particularly, plasma cell (spot H), proliferative (spot D), BL-resembling (spots C and B) and certain B-cell (spot L) properties associate with higher HRs, while inflammatory (spot F), stromal (spot G) and FL-resembling (spot I) characteristics associate with lower HRs in most of the subtypes. These findings suggest that different molecular phenotypes associate with similar prognostic trends partly independent of the respective subtype.

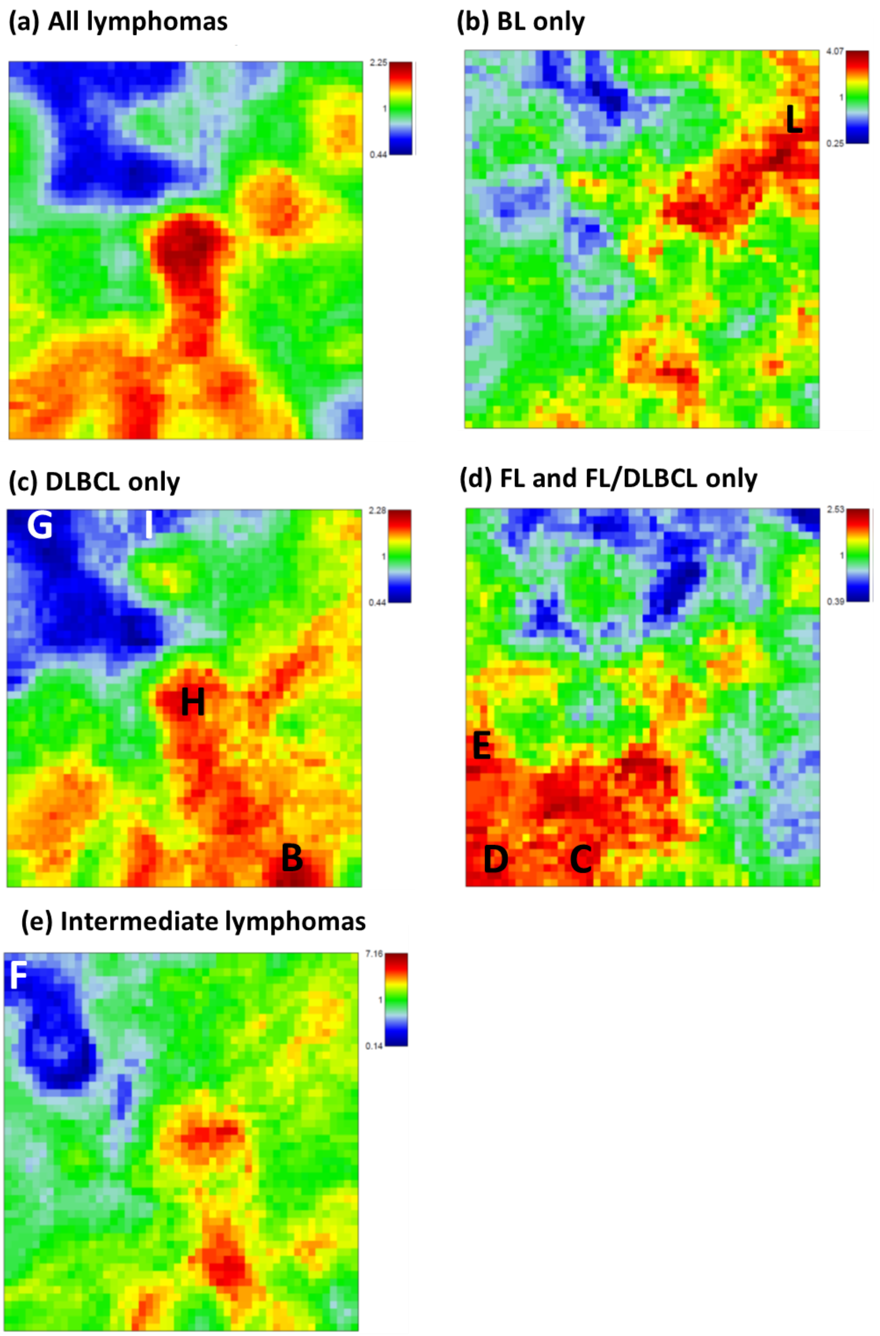

Figure S 15: Prognostic maps were calculated considering all lymphomas (part a) or only selected subtypes as indicated in the figure (b – e). The maps show a common trend, namely the better prognosis for tumors with activated inflammatory and stromal DLBCL-like characteristic (blue regions in the upper part of the map), and worse prognosis for tumors with activated proliferative and BL-like characteristics (red regions in the lower part of the map). The letters in the maps indicate the spot regions. Note that the individual color scale used in each of the maps refers to the corresponding HR range.

Phylogenetic trees with sample, subtype and PAT resolution

We apply the neighbor-joining algorithm to visualize similarity relations between the samples in terms of a phylogenetic cluster tree. We performed phylogenetic clustering on three levels, namely on the individual samples, on the lymphoma subtypes and classes of reference samples, and on the PATs (see Figure S 16a, b, and c, respectively). The finest resolution on sample level provides a bush-like dendrogram where the vast amount of cases, however, hampers its detailed evaluation. On the other hand, the subtype level is obviously too rough and over-simplifies the similarity relations between data. The intermediate, PAT level provides an optimal resolution of the intra-subtype variability (Figure S 16c).

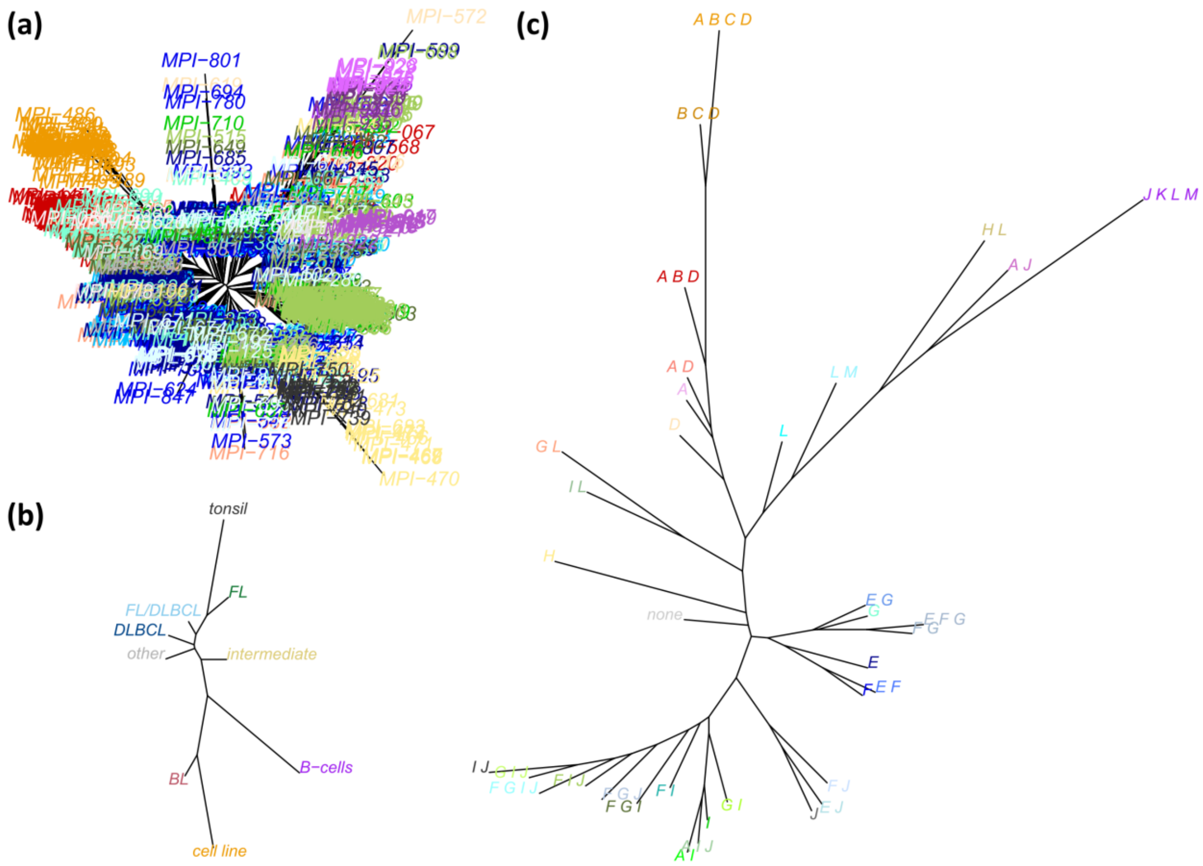

Figure S 16: Phylogenetic cluster trees of (a) all 914 individual cases, (b) the nine subtypes and control groups, and (c) the 36 pattern-types. The distances between leaves are scaled proportionally to their mutual dissimilarity measured in terms of the Euclidian distances.

Zoom-in SOM analysis of lymphoma cell lines and of B-cells

Lymphoma cell lines and B-cells densely accumulate in relatively narrow regions in the similarity network (Figure 1b) what reflects the very similar SOM portraits of both classes (Figure S 3). For a more detailed evaluation of the transcriptomic landscapes of lymphoma cell lines and B-cells, we trained zoom-in SOMs separately for each of the reference cell systems [10]. They make use of the respective subset of samples instead of the whole set of samples used for the SOM described in the main article. This 'zoom-in' approach reduces the transcriptomic state space and thus is expected to provide an increased resolution of the expression landscape of lymphoma cell lines (Figure S 17) and of the B-cells (Figure S 18), respectively.

Similarity analysis of the SOM portraits of the lymphoma cell line samples reveals three major regions, enriched either by BL-like (including BL-, BCL- and mnBLL-11q cells), by DLBCL-like (including DLBCL-, PMBL- and DH BL-cells), or by MM-like (including MM- and plasma cell myeloma cells), respectively (Figure S 17a). Their mean ('zoom-in'-) SOM-portraits show that these three types are characterized by activation of specific spot patterns which were visualized in the spot over-expression summary map together with the functional context of the spots (Figure S 17b) and selected key genes (Figure S 17c). Note that the spot patterns of the zoom-in SOM is different from that of the original full SOM and it, therefore, requires separate annotations. As expected, it expresses details of the transcriptome landscape with higher resolution (compare with the mean portraits of the cell line types in Figure S 3). For example, BL cell lines upregulate the BL tumor signature [22] together with the ID3 gene expression as their hallmarks while MM cell lines activate the plasma cell signature [37] together with PRDM1. The DLBCL-like cells upregulate a more diverse spot pattern including functions related to inflammation (MHC-class II and interferon response) and to epigenetic effects (EZH2- and histone-deacetylases (HDAC)-targets). Interestingly, the EZH2 gene upregulates in DLBCL-like and BL-like (especially mnBLL-11q) cells as well. In contrast, the PIM1 gene links MM-like and DLBCL-like cells, a relation which was also found in lymphoma with ABC-characteristics sharing partly features of GCB and plasma cells. PMBL cells express an interferon response signature in combination with BCL6 repression and STAT6 activation in agreement with [48]. Double hit BL cells show a specific spot enriching genes of pre/post B cells.

The B-cell samples essentially divide into GCB-cells and pre/post GCB-cells with regard to the similarity tree (Figure S 18a) and the spot expression patterns (Figure S 18b, c). Pre-GCB-cells show a deactivated signature of mature B-cells expressing MYC and BCL2 compared with post-GCB cells. GCB-cells upregulate BCL6 and EZH2 and the BL signature [22]. Epstein-Barr virus infected GCB-cells, in addition, upregulate interferon response genes in agreement with [49].

Notably, the respective similarity sub-trees derived from the complete data show a very similar structure to the trees derived from the zoom-in analyses (Figure S 17d and Figure S 18d). This agreement reflects the fact, that the original SOM already resolves essential details of the transcriptional landscapes of the lymphoma cell lines and of the B-cell samples. Their spot patterns are partly masked in the corresponding expression portraits because of the red cross-fading effect discussed above. To better resolve the fine structure of the transcriptomic landscape, we generated class-related difference portraits by subtracting the mean portrait of lymphoma cell lines or B-cells, respectively, from the portraits of the different cell types. In consequence, this step removes the cross-fading effect (see the SOM portraits in Figure S 17d and Figure S 18d). Importantly, the class-related difference SOM portraits obtained can be directly compared and interpreted in terms of the spot patterns annotated in the original 'full' map. We find clear differences, e.g., between BL and DLBCL cell lines over-expressing either spot A or F, respectively, corresponding to the expression patterns observed in the BL and

DLBCL tumor samples (Figure S 17d). The portraits of the MM- and plasma cell myeloma cell lines show strong expression of spots K and H reflecting their cell of origin. In conclusion, the SOM map of the complete data set clearly resolves details of the expression landscapes after appropriate adjustment. The same result is obtained for the B-cell samples (Figure S 18d). The GC-B-cells over-express the proliferative spot D, contrary to the pre- & post-GC-B-cells. Note also that EBV-infected cells express the inflammatory spot F harboring a series of interferon response markers.

In summary, the zoom-in analyses reveal specific details of the transcriptomes of lymphoma cell line and B-cell specimen with high resolution. A similar resolution is however already obtained from the SOM of the complete data set after appropriate adjustment.

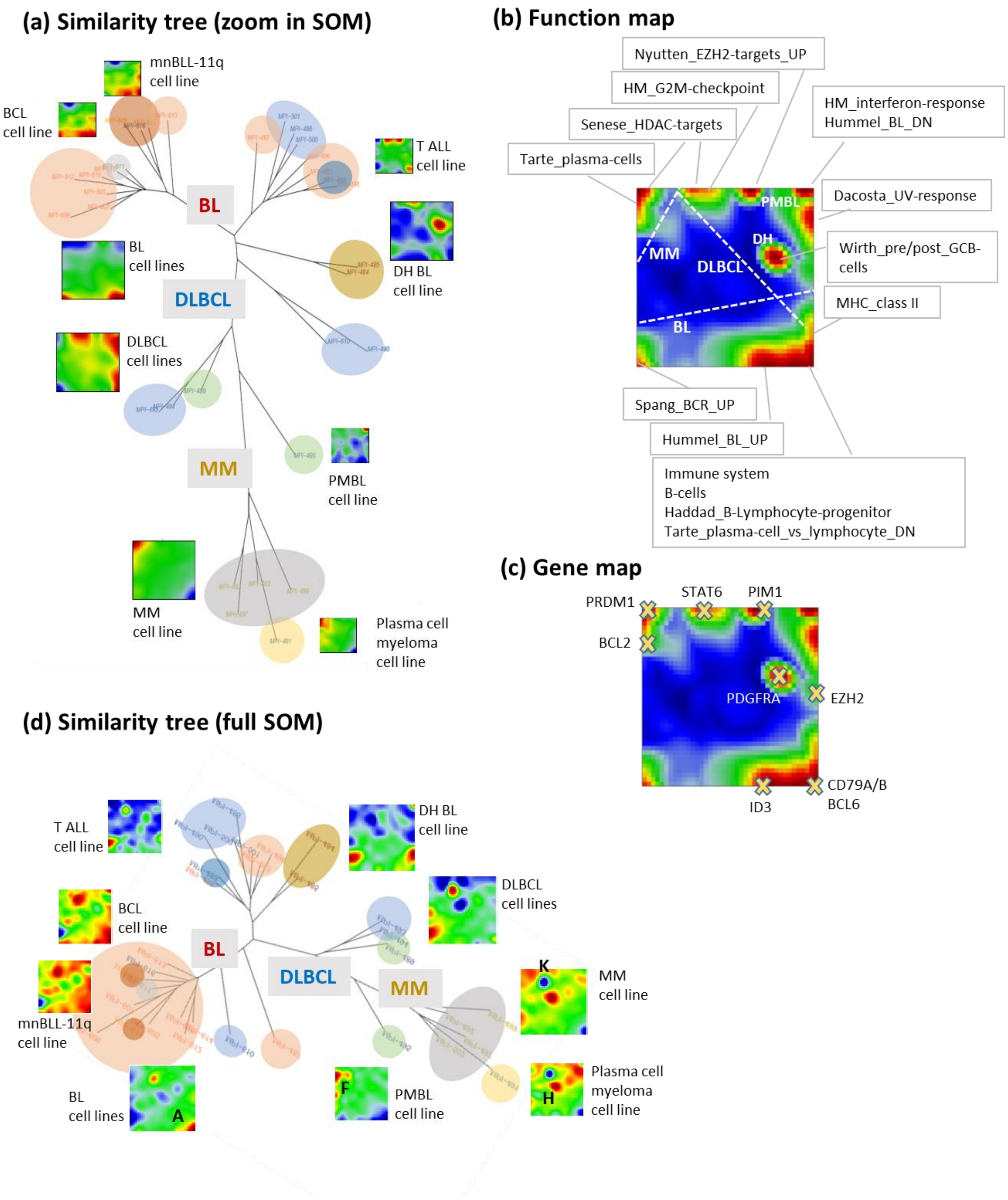

Figure S 17: Zoom-in SOM analysis of lymphoma cell lines: (a) The similarity tree divides into three major areas which contain mainly BL, DLBCL, and MM cell lines, respectively (from top to bottom). Mean portraits of the cell line types were shown near the respective leaves. Each cell line type is indicated by differently colored areas at the end of the respective branches. (b) The over-expression spot map of the zoom-in SOM divides into three areas referring to spots upregulated in DLBCL- BL- and MM- cell lines, respectively. The functional context of the spots was estimated by means of gene set enrichment analysis. (c) The gene map shows the location of selected key genes in the zoom-in SOM (see also Figure 3a). (d) The similarity tree of the cell line samples based on the original 'full' data SOM analysis as presented in the main article closely resembles the similarity of the zoom-in SOM (compare with part a). This reflects the fact that both metagene-expression landscapes are nearly identical regarding the cell line samples. This result is further illustrated by mean portraits of the different cell line types referring to the original SOM. To correct for the red cross-fading effect we generated class-related difference portraits by subtracting the mean portrait averaged over all cell line samples from each of the individual portraits. These class-related differences show up- and down-regulated spots more clearly (compare with the respective portraits in Figure S 3), and their patterns can be interpreted using their functional annotations as described in the main article.

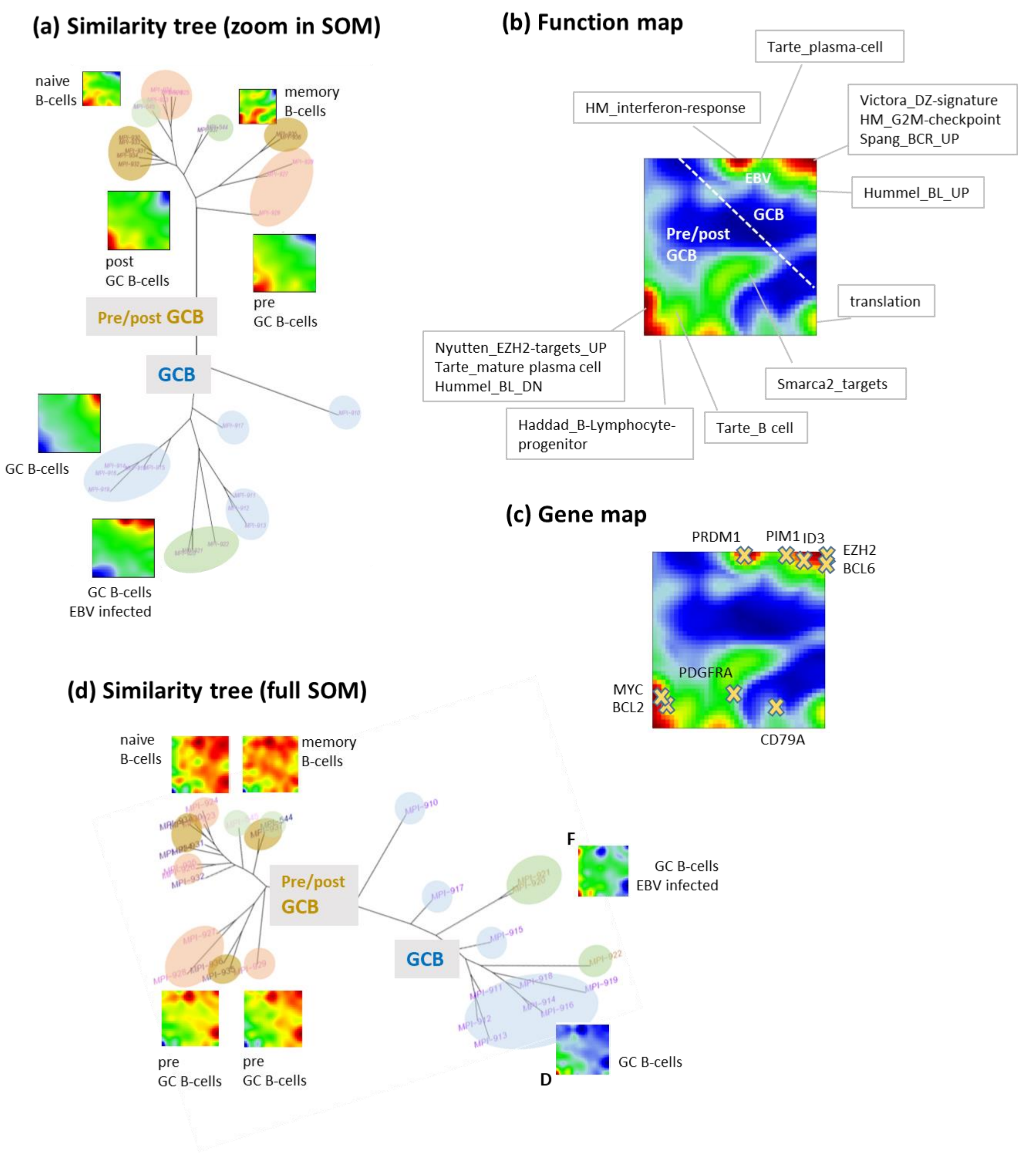

Figure S 18: Zoom-in SOM analysis of sorted B-cells: (a) The similarity tree divides into two major sample groups which contain mainly GCB and pre/post-GCB cells, respectively. Mean portraits of the B-cell types were shown near the respective leaves. Each B-cell type is illustrated by differently colored areas at the end of the respective branches. (b) The over-expression spot map of the zoom-in SOM divides into two areas referring to spots upregulated in GCB- and pre/post GCB cells, respectively. The functional context of the spots was estimated by means of gene set enrichment analysis. (c) The gene map shows the location of selected key genes in the zoom-in SOM (see also Figure 3a and Figure S 17). (d) Similarity tree of the B-cell samples based on the original ‘full’ data SOM analysis. The class-related difference portraits better resolve the spot patterns after subtracting the mean portrait averaged over all B-cell portraits, virtually removing the cross-fading effect in the original portraits shown in Figure S 3.

Zoom-in SOM analysis of Burkitt's Lymphomas

A 'zoom-in' SOM analysis of BL was performed taking into account only the transcriptomic data of tumors of this subtype. The resulting similarity tree splits into three major parts differing in the expression landscapes of the respective BL-tumors included (Figure S 19a). The first group of tumors can be assigned to cases showing high expression of the BL expression signature and of proliferative markers (Figure S 19b and c). The second group of BL tumors reveals a relatively high activity of inflammatory signatures and reduced expression of the BL signature. The third group upregulates target genes of the polycomb repressive complex 2 (PRC2) usually activated in weakly differentiated cells, and genes also upregulated in healthy pre-/post-GCB B-cells. For comparison with the spots identified in the original 'full' SOM, we mapped the respective spot-genes into the zoom-in SOM (Figure S 19d). Most of these genes accumulate in the spot-regions of the zoom-in SOM. Based on this local accumulation we established a virtually one-to-one correspondence between most of the spots in the two different maps as indicated by the letters in Figure S 19b referring to the modules A - M in the full SOM. The spot module expression profiles differ systematically between the three BL groups identified (Figure S 19d). A similarity tree of the SOM portraits of the BL tumors taken from the original SOM shows a very similar structure as the tree of the zoom-in SOM (Figure S 19f). This agreement indicates that the full SOM expresses details of the respective transcriptomic landscapes of the BLs already with sufficient resolution, as also shown above for cell line and B-cell samples. Detailed inspection of the spot module profiles of the full SOM supports this conclusion (Figure S 5). For example, the profiles of the proliferative spot D and of the inflammatory spot F reveal a gradient within the BLs, which is simply amplified in the zoom-in SOM. These profiles also show that the expression level of the inflammatory spot F in BLs is markedly smaller than in DLBCL and FL. Hence, the annotation of the 'inflammatory' BL group means that it includes tumors with activated inflammatory characteristics (e.g. of HLA type I and II genes, Figure S 19e) on a relative scale, but still on markedly lower levels compared to DLBCL (Figure S 5).

Summarizing, we find that detailed analysis of the relatively homogeneous molecular entity of BL can be split into subgroups with distinct expression characteristics regarding proliferative, inflammatory and developmental B-cell programs in agreement with a previous gene expression study of BL [50]. It reported subgroup differences regarding cell cycle control, B-cell and TNF-kappaB signaling. We also found that the resolution of the full SOM is sufficiently high for detailed subtype-specific analyses.

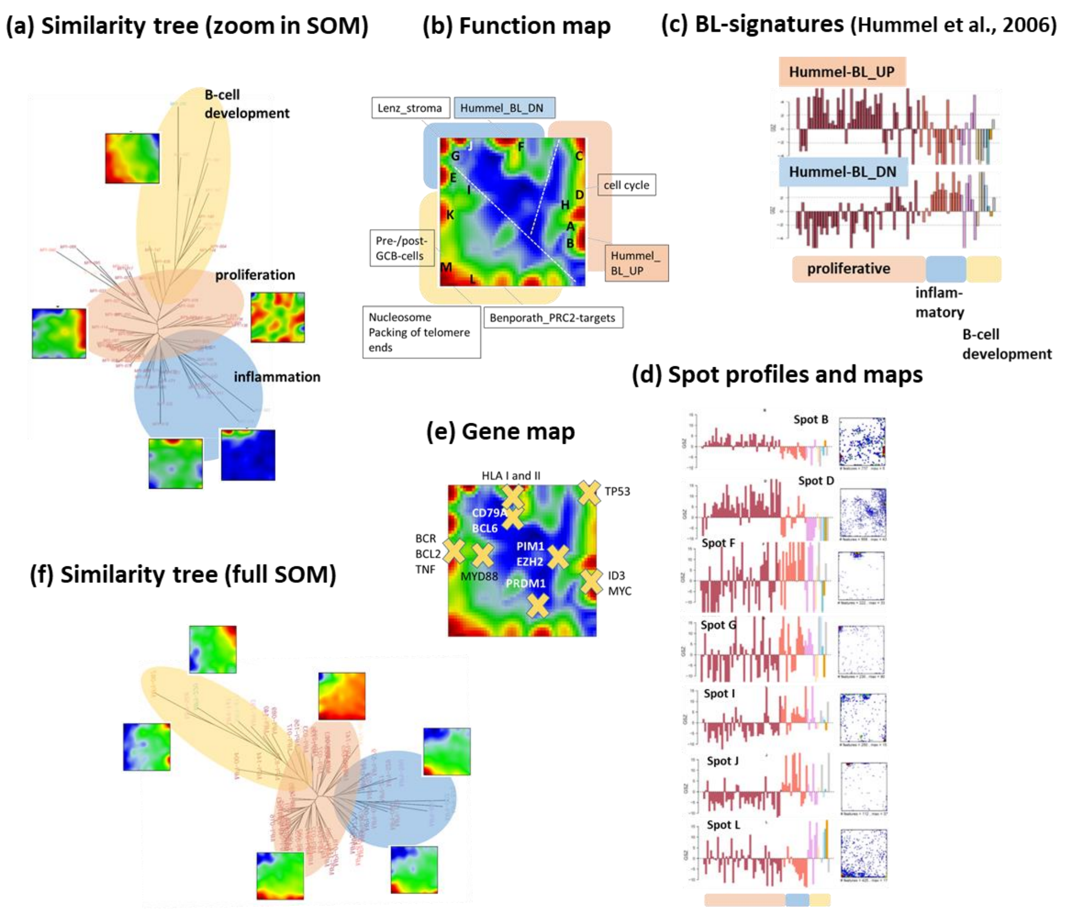

Figure S 19: Zoom-in SOM analysis of BL cases: (a) The similarity tree reveals three major subgroups of BL which are characterized as 'proliferative', 'inflammatory' and 'PRC2-repressed' according to their expression patterns. (b) The over-expression summary map roughly divides into three major spot groups which upregulate in the respective BL-groups. The capital letters indicate spot module correspondence with the modules annotated in the full SOM. (c) The molecular BL signature [22] upregulates in the proliferative BL-group while it downregulates in the two other groups. (d) Module genes identified in the full SOM were mapped into the zoom-in map to visualize their distribution (dots mark metagenes which contain at minimum one spot gene). Most of the module genes accumulate in areas of the map which are identified also as spots in the zoom-in map. The spot profiles reveal BL-group specific up- and down-regulation. Not all spots are shown. (e) Mapping of selected genes into the zoom-in SOM. (f) The similarity tree of the SOM portraits of the full SOM closely resembles that of the zoom-in SOM. This agreement indicates that the full SOM ensures sufficient resolution to study details of the BL transcriptomes.

4 Tables

Stratification of the MMML cohort

Table S 1: Stratification of the MMML-cohort

**913 specimen**	**Description**
**32** cancer cell lines, see [21, 51] for details	Samples of different cancer cell lines used as references
**11** BL cell line samples	Burkitt's lymphoma cell lines: BL-41, BLUE-1, CA-46, DG-75, Daudi, Gumbus, Ramos, Namalwa (2x), Raji (2x)
**8** DLBCL cell line samples	Diffuse large B-cell lymphoma cell lines: DoHH2, Ly3, Ly10, Su-DHL-4, Su-DHL-6 (2x), Karpas-422 (2x)
**5** MM cell lines	Multiple myeloma cell lines: LP-1, OPM2, RPMI8226, U266, L363
**2** PMBL cell lines	Primary mediastinal B-cell lymphoma cell lines Karpas1106, MedB1
**2** DH-BL cell lines	Double-hit BL cell lines: DoGKit, DoGum
**2** mnBLL-11q cell lines	Burkitt-like cell line with 11q aberration Su-DHL-5, HT
1 T-ALL cell line	T-cell acute lymphoblastic leukemia cell line: JM1
1 BCL cell line	B-cell non-Hodgkin lymphoma cell line: MC-116
**40** Reference tissues	
**30** B-cell specimen	Healthy B-cell and Epstein-Barr-Virus (EBV) B-cell specimen
**10** Germinal center (GC) B-cells	
**8** Post GC B-cells	
**7** Pre GC B-cells	
**3** EBV positive GC B-cells	
**1** Naive B-cells	
**1** Memory B-cells	
**10** Tonsils	Healthy reference specimen
**841** Lymphoma specimen	Tumor specimen collected within the MMML-consortium classified according to histopathological panel review
**430** DLBCL	Diffuse large B-cell lymphoma
**245** centroblastic	Histopathological classification according to [24, 25]
**29** immunoblastic	
**12** plasmoblastic	
**11** anaplastic	
**133** unclassified	
**145** FL	Follicular lymphoma without DLBCL or FL3b component
**69** grade 1	Histopathological classification
**35** grade 2	
**32** grade 3a	
**9** unclassified	
**81** intermediate lymphoma	Hummel classification ‘intermediate’ or ‘mBL’, but no prototypical Burkitt’s lymphoma and no double hit
**11** BL-like	IG-MYC positive and Burkitt morphology, or IG-MYC positive and age<40 years and BL-Score > 0.5
**70** other	all other intermediate cases

**74**	prototypical BL	Classified as molecular Burkitt's lymphoma (mBL) and IG-MYC positive and no t(14;18) and no BCL6-break
**48**	FL/DLBCL	FL grade 3b, or DLBCL and FL components; no double hit
**23**	PMBL	Primary mediastinal B-cell lymphoma; no double hit
**20**	Multiple myeloma	Plasmacytoma or multiple myeloma
**10**	IRF4-break positive lymphoma	Interferon regulatory factor 4 -break positive, see [52]
**6**	mnBLL-11q	Burkitt-like lymphoma with 11q aberration, see [53]
**4**	MCL	Mantle cell lymphoma

Alternative molecular classification according to Hummel et al. [22]

**602**	non-mBL	non-molecular Burkitt's lymphoma with low BL-Score
**156**	intermediate	intermediate BL-Score
**83**	mBL	molecular Burkitt's lymphoma with high BL-Score

Alternative molecular classification according to Rosolowski et al. [54]

**189**	HiGA-Sir	High gene activity with stromal and immune response cluster
**106**	HiGA-Pro	High gene activity with proliferative phenotype cluster
**69**	LowGA	Low gene activity cluster
**477**	unclassified	

Alternative molecular classification according to Bentink et al. [55]

**78**	BL-PAP	Burkitt-like expression pattern
**345**	PAP-1	DLBCL-like expression patterns 1 – 4
**63**	PAP-2	
**71**	PAP-3	
**81**	PAP-4	
**203**	mind-L	Molecularly individual lymphomas

Alternative molecular classification according to Wright et al. [30]

**506**	GCB	Cell of origin: germinal center B-cell
**166**	ABC	Cell of origin: activated B-cell
**169**	unclassified	

Alternative genomic classification according to IG-MYC translocation

**134**	IG-MYC translocation positive	Aberration detected by fluorescence in situ hybridization
**35**	non-IG-MYC translocation positive	
**672**	MYC translocation negative	

Alternative genomic classification according to IG-BCL2 translocation

**204**	IG-BCL translocation positive	Aberration detected fluorescence in situ hybridization
**637**	IG-BCL translocation negative	

Alternative genomic classification according to BCL6 break

**179**	BCL6 break positive	Aberration detected fluorescence in situ hybridization
**662**	BCL6 break negative	

Alternative genomic classification according to IGH break

**448**	IGH break positive	Aberration detected fluorescence in situ hybridization
**393**	IGH break negative	

Alternative genomic classification of single-hit lymphomas

**170** IG-BCL2 positive<br>**117** IG-MYC positive<br>**18** non-IG-MYC positive<br>**536** negative	Aberration detected fluorescence in situ hybridization

Alternative genomic classification of double-hit lymphomas

**17** IG-BCL2 & IG-MYC positive<br>**17** IG-BCL2 & non-IG-MYC positive<br>**807** negative	Aberration detected fluorescence in situ hybridization

Phenotypic and molecular characterization of the lymphoma subtypes

Table S 2: Phenotypic and molecular characterization of the lymphoma subtypes. Enriched characteristics are highlighted using bold typeset (p-value <0.001 in Fisher's exact test).

Characteristic [a]		Total	Lymhoma subtype					
			BL	Intermed.	DLBCL	FL/DLBCL	FL	other
Total	number of cases	842	74 (8 %)	81 (10 %)	430 (51 %)	48 (6 %)	145 (17 %)	63 (8%)
Age	≤18 y	86 (9%)	**43 (58%)**	13 (16%)	18 (4%)	1 (2%)	1 (1%)	10 (16%)
	19-59 y	359 (39%)	26 (35%)	28 (35%)	160 (37%)	24 (50%)	**94 (65%)**	27 (43%)
	≥ 60 y	370 (40%)	5 (7%)	37 (46%)	**242 (56%)**	23 (48%)	46 (32%)	16 (25%)
Gender	Male	439 (48%)	**54 (73%)**	43 (53%)	223 (52%)	25 (52%)	67 (46%)	27 (43%)
	Female	388 (42%)	20 (27%)	37 (46%)	205 (48%)	23 (48%)	75 (52%)	27 (43%)
Ann Arbor stage	I or II	229 (25%)	22 (30%)	27 (33%)	118 (27%)	14 (29%)	32 (22%)	16 (25%)
	III or IV	283 (31%)	17 (23%)	20 (25%)	152 (35%)	14 (29%)	70 (48%)	9 (14%)
Molecular classification Hummel *et al.*	mBL	83 (9%)	**74 (100%)**	2 (2%)	3 (1%)	1 (2%)	0 (0%)	3 (5%)
	Intermediate	156 (17%)	0 (0%)	**79 (98%)**	29 (7%)	12 (25%)	25 (17%)	11 (17%)
	non-mBL	603 (66%)	0 (0%)	0 (0%)	**398 (93%)**	35 (73%)	**120 (83%)**	49 (78%)
GCB-ABC classification Wright *et al.*	Activated B-cells	166 (18%)	0 (0%)	11 (14%)	**138 (32%)**	7 (15%)	5 (3%)	5 (8%)
	Germinal center B-cells	507 (55%)	**69 (93%)**	59 (73%)	188 (44%)	32 (67%)	**120 (83%)**	38 (60%)
MYC translocation	IG-*MYC*	134 (15%)	**71 (96%)**	**26 (32%)**	32 (7%)	2 (4%)	3 (2%)	0 (0%)
	non-IG-*MYC*	35 (4%)	0 (0%)	4 (5%)	24 (6%)	1 (2%)	4 (3%)	2 (3%)
	Negative	639 (70%)	0 (0%)	50 (62%)	360 (84%)	40 (83%)	137 (94%)	51 (81%)
BCL6 BREAK	Positive	179 (20%)	0 (0%)	3 (4%)	**130 (30%)**	11 (23%)	26 (18%)	9 (14%)
	Negative	623 (68%)	**70 (95%)**	**76 (94%)**	283 (66%)	32 (67%)	117 (81%)	44 (70%)
IGH BREAK	Positive	446 (49%)	**66 (89%)**	40 (49%)	172 (40%)	20 (42%)	**121 (83%)**	27 (43%)
	Negative	337 (37%)	5 (7%)	37 (46%)	**229 (53%)**	21 (44%)	19 (13%)	25 (40%)
t(14;18)	Positive	202 (22%)	0 (0%)	10 (12%)	64 (15%)	13 (27%)	**114 (79%)**	1 (2%)
	Negative	605 (66%)	**71 (96%)**	70 (86%)	**352 (82%)**	30 (62%)	29 (20%)	**52 (83%)**
CD10 expression	Low [b]	373 (41%)	4 (5%)	24 (30%)	**284 (66%)**	12 (25%)	14 (10%)	**35 (56%)**
	High	416 (46%)	**67 (91%)**	54 (67%)	127 (30%)	31 (65%)	**122 (84%)**	14 (22%)
BCL2 expression	Low	188 (21%)	**57 (77%)**	25 (31%)	80 (19%)	6 (12%)	8 (6%)	12 (19%)
	high	607 (66%)	13 (18%)	53 (65%)	**331 (77%)**	41 (85%)	131 (90%)	37 (59%)
BCL6 expression	Low	104 (11%)	1 (1%)	12 (15%)	**72 (17%)**	5 (10%)	2 (1%)	12 (19%)
	high	573 (63%)	**67 (91%)**	59 (73%)	281 (65%)	32 (67%)	101 (70%)	32 (51%)
MUM1 expression	Low	284 (31%)	39 (53%)	35 (43%)	117 (27%)	21 (44%)	**60 (41%)**	11 (17%)
	high	359 (39%)	24 (32%)	33 (41%)	**237 (55%)**	15 (31%)	18 (12%)	32 (51%)
HLADR expression	Low	124 (14%)	19 (26%)	9 (11%)	74 (17%)	3 (6%)	6 (4%)	13 (21%)
	high	487 (53%)	43 (58%)	56 (69%)	251 (58%)	30 (62%)	**78 (54%)**	28 (44%)
KI67 expression	Low	557 (61%)	5 (7%)	44 (54%)	299 (70%)	42 (88%)	**127 (88%)**	39 (62%)
	high	242 (26%)	**66 (89%)**	36 (44%)	117 (27%)	5 (10%)	6 (4%)	12 (19%)

[a] Parameters are not available for all cases. Data are taken from ref [56].
[b] Thresholds for classification of IHC markers in high and low groups are described in ref. [56].

Meta gene sets characterizing hallmarks of cancer

Cancer hallmarks provide a reductionist view on processes involved in tumor genesis and progression [47]. We generate meta-genesets from different GO- and literature-sets according to functions and processes related to the respective cancer hallmark. These so-called hallmark sets are used in enrichment analyses analogous to the 'single' gene sets. Table S 3 summarizes the hallmark sets in terms of number of genes and the individual gene sets included. Please note that there are no meta-genesets defined for the hallmarks 'evading growth suppression' and 'evading immune detection' due to the lack of specific processes attributed to those capabilities.

Table S 3: Cancer hallmark gene sets are assembled from diverse predefined GO and literature gene sets. Number of genes and individual member gene sets are given for the eight hallmark sets.

Hallmark	Number of Genes [a]	Contained Gene Sets
Angiogenesis	141 / 258	GO: blood vessel development<br>GO: positive regulation of angiogenesis<br>GO: sprouting angiogenesis
Controlling Genomic Instability	358 / 575	GO: DNA repair<br>GO: response to DNA damage stimulus<br>GO: response to ionizing radiation<br>GO: response to UV
Glucose Energetics	49 / 78	GO: glycolytic process<br>GO: positive regulation of glycolytic process
Inflammation	279 / 416	GO: inflammatory response<br>GO: positive regulation of inflammatory response
Invasion and Metastasis	165 / 336	GO: cell motility<br>GO: negative regulation of cell adhesion<br>GO: negative regulation of cell-cell adhesion<br>GO : negative regulation of cell-matrix adhesion<br>Alonso et al.: metastasis UP [57]<br>Alonso et al.: metastasis EMT UP [57]<br>Bidus et al.: metastasis UP [58]<br>Cromer et al.: metastasis UP [59]<br>Jaeger et al.: metastasis UP [60]<br>O'Donnell et al.: metastasis UP [61]<br>Ramaswamy: metastasis UP [62]<br>Vicent et al.: metastasis UP [63]
Proliferation	685 / 1129	GO: cell cycle<br>GO: DNA replication<br>GO: positive regulation of cell growth<br>GO: positive regulation of cell proliferation
Replicative Immortality	102 / 154	GO: chromosome, telomeric region<br>GO: telomere maintenance<br>Reactome: extension of telomeres [64]<br>Dairkee et al.: TERT targets UP [65]<br>Kang et al.: immortalized by TERT UP [66]
Resisting Death	435 / 752	GO: negative regulation of apoptotic process<br>GO: negative regulation of autophagy

[a] Number of Ensembl gene ids / Affymetrix gene ids in the hallmark set

Survival curve comparisons

Table S 4: Hazard ratios and p-values derived from Cox models for all pairwise comparisons of the survival curves given in the main article. Models were additionally adjusted by inclusion of co-factors ‘chemotherapy’ (yes/no) and ‘Rituximab’ (yes/no). Cases without information about therapy were removed from the multivariate model. Pairs of strata which are significant with p-value < 0.01 after adjustment for therapy are marked with an asterisk and bold style.

Hallmark types

	Weakly carcinogenic	Proliferative	Balanced proliferative	Inflammatory	Balanced inflammatory
Weakly carcinogenic		0.9 (0.65)	0.5 (< 0.01)	0.96 (0.85)	1.47 (0.16)
Proliferative	1.11 (0.65)		0.58 (0.01)	1.1 (0.61)	1.6 (0.07)
Balanced proliferative	1.98 (< 0.01)	1.72 (0.01)		1.96 (< 0.01)	**2.82 (< 0.01) ***
Inflammatory	1.04 (0.85)	0.91 (0.61)	0.51 (< 0.01)		1.47 (0.10)
Balanced inflammatory	0.68 (0.16)	0.62 (0.07)	**0.36 (< 0.01) ***	0.68 (0.10)	

Subtypes

	BL	intermediate	DLBCL	FL/DLBCL	FL	other
BL		0.76 (0.30)	0.63 (0.02)	1.43 (0.30)	1.2 (0.46)	1.74 (0.11)
intermediate	1.31 (0.30)		0.85 (0.38)	2.11 (0.03)	1.74 (0.02)	2.28 (0.02)
DLBCL	1.6 (0.02)	1.18 (0.38)		2.38 (< 0.01)	**2.19 (< 0.01) ***	**2.87 (< 0.01) ***
FL/DLBCL	0.7 (0.30)	0.47 (0.03)	0.42 (< 0.01)		1.01 (0.97)	1.29 (0.54)
FL	0.83 (0.46)	0.57 (0.02)	**0.46 (< 0.01) ***	0.99 (0.97)		1.55 (0.19)
other	0.57 (0.11)	0.44 (0.02)	**0.35 (< 0.01) ***	0.77 (0.54)	0.65 (0.19)	

DLBCL ABC vs. GCB

	ABC	GCB
ABC		**2.2 (< 0.01) ***
GCB	**0.45 (< 0.01) ***	

BL in children vs. adults

	Child	Adult
Child		0.4 (0.02)
Adult	2.52 (0.02)	

PAT types

	AD	E	F	G	FIJ	I	L	H	IJ
AD		0.55<br>(0.03)	0.74<br>(0.22)	1.38<br>(0.23)	1.35<br>(0.31)	1.86<br>(0.03)	1.04<br>(0.91)	0.51<br>(0.15)	1.13<br>(0.71)
E	1.81<br>(0.03)		1.3<br>(0.23)	2.41<br>(<0.01)	2.39<br>(<0.01)	**3.42**<br>**(<0.01) ***	1.86<br>(0.04)	0.87<br>(0.75)	2.04<br>(0.02)
F	1.35<br>(0.22)	0.77<br>(0.23)		**1.89**<br>**(<0.01) ***	1.98<br>(<0.01)	**2.8**<br>**(<0.01) ***	1.48<br>(0.17)	0.74<br>(0.49)	1.56<br>(0.12)
G	0.73<br>(0.23)	0.41<br>(<0.01)	**0.53**<br>**(<0.01) ***		1<br>(0.99)	1.39<br>(0.20)	0.79<br>(0.45)	0.35<br>(0.02)	0.84<br>(0.57)
FIJ	0.74<br>(0.31)	0.42<br>(<0.01)	0.51<br>(<0.01)	1<br>(0.99)		1.57<br>(0.12)	0.81<br>(0.52)	0.35<br>(0.02)	0.85<br>(0.61)
I	0.54<br>(0.03)	**0.29**<br>**(<0.01) ***	**0.36**<br>**(<0.01) ***	0.72<br>(0.20)	0.64<br>(0.12)		0.56<br>(0.07)	0.18<br>(<0.01)	0.6<br>(0.11)
L	0.96<br>(0.91)	0.54<br>(0.04)	0.68<br>(0.17)	1.26<br>(0.45)	1.24<br>(0.52)	1.79<br>(0.07)		0.48<br>(0.13)	1.06<br>(0.88)
H	1.95<br>(0.15)	1.15<br>(0.75)	1.35<br>(0.49)	2.82<br>(0.02)	2.84<br>(0.02)	5.64<br>(<0.01)	2.07<br>(0.13)		2.78<br>(0.04)
IJ	0.89<br>(0.71)	0.49<br>(0.02)	0.64<br>(0.12)	1.19<br>(0.57)	1.18<br>(0.61)	1.65<br>(0.11)	0.95<br>(0.88)	0.36<br>(0.04)	

DLBCL related PATs

	DLBCL reference	DLBCL A	DLBCL E	DLBCL EF	DLBCL F	DLBCL FG	DLBCL G	DLBCL none
DLBCL reference		0.46<br>(0.06)	0.77<br>(0.22)	1.66<br>(0.12)	0.74<br>(0.08)	2.17<br>(0.02)	1.48<br>(0.25)	0.72<br>(0.05)
DLBCL A	2.18<br>(0.06)		1.58<br>(0.32)	3.65<br>(0.01)	1.66<br>(0.25)	4.39<br>(< 0.01)	3.38<br>(0.03)	1.65<br>(0.26)
DLBCL E	1.3<br>(0.22)	0.63<br>(0.32)		2.08<br>(0.05)	0.98<br>(0.94)	2.97<br>(< 0.01)	2<br>(0.08)	0.93<br>(0.76)
DLBCL EF	0.6<br>(0.12)	0.27<br>(0.01)	0.48<br>(0.05)		0.45<br>(0.02)	1.27<br>(0.60)	0.98<br>(0.96)	0.44<br>(0.02)
DLBCL F	1.35<br>(0.08)	0.6<br>(0.25)	1.02<br>(0.94)	2.24<br>(0.02)		**2.98**<br>**(< 0.01) ***	2.1<br>(0.05)	0.98<br>(0.92)
DLBCL FG	0.46<br>(0.02)	0.23<br>(< 0.01)	0.34<br>(< 0.01)	0.79<br>(0.60)	**0.34**<br>**(< 0.01) ***		0.65<br>(0.37)	0.3<br>(< 0.01)
DLBCL G	0.68<br>(0.25)	0.3<br>(0.03)	0.5<br>(0.08)	1.02<br>(0.96)	0.48<br>(0.05)	1.54<br>(0.37)		0.43<br>(0.03)
DLBCL none	1.4<br>(0.05)	0.6<br>(0.26)	1.08<br>(0.76)	2.29<br>(0.02)	1.02<br>(0.92)	3.3<br>(< 0.01)	2.31<br>(0.03)	

Table S 5: Hazard ratios and p-values derived from adjusted Cox models are given for co-factors ‘groups’ (rows labelled with ‘G:’), ‘chemotherapy’ (‘C:’), and ‘Rituximab therapy’ (‘R:’).

Hallmark types

	Weakly carcinogenic	Proliferative	Balanced proliferative	Inflammatory	Balanced inflammatory
Weakly carcinogenic		G: 1.20 (0.54)<br>R: 1.20 (0.67)<br>C: 1.28 (0.60)	G: 0.68 (0.17)<br>R: 0.46 (0.14)<br>C: 1.07 (0.85)	G: 1.08 (0.75)<br>R: 0.55 (0.04)<br>C: 1.38 (0.27)	G: 2.26 (0.01)<br>R: 0.51 (0.22)<br>C: 1.89 (0.16)
Proliferative	G: 0.84 (0.54)<br>R: 1.20 (0.67)<br>C: 1.28 (0.60)		G: 0.67 (0.14)<br>R: 0.80 (0.65)<br>**C: 0.33 (< 0.01)**	G: 0.9 (0.62)<br>R: 0.73 (0.27)<br>C: 0.75 (0.32)	G: 2.07 (0.03)<br>R: 1.23 (0.68)<br>C: 0.41 (0.06)
Balanced proliferative	G: 1.47 (0.17)<br>R: 0.46 (0.14)<br>C: 1.07 (0.85)	G: 1.48 (0.14)<br>R: 0.80 (0.65)<br>**C: 0.33 (< 0.01)**		G: 1.62 (0.02)<br>R: 0.50 (0.03)<br>C: 0.74 (0.24)	**G: 3.1 (< 0.01)**<br>R: 0.30 (0.10)<br>C: 0.46 (0.03)
Inflammatory	G: 0.93 (0.75)<br>R: 0.55 (0.04)<br>C: 1.38 (0.27)	G: 1.11 (0.62)<br>R: 0.73 (0.27)<br>C: 0.75 (0.32)	G: 0.62 (0.02)<br>R: 0.50 (0.03)<br>C: 0.74 (0.24)		G: 1.93 (0.02)<br>R: 0.55 (0.06)<br>C: 0.94 (0.82)
Balanced inflammatory	G: 0.44 (0.01)<br>R: 0.51 (0.22)<br>C: 1.89 (0.16)	G: 0.48 (0.03)<br>R: 1.23 (0.68)<br>C: 0.41 (0.06)	**G: 0.32 (< 0.01)**<br>R: 0.30 (0.10)<br>C: 0.46 (0.03)	G: 0.52 (0.02)<br>R: 0.55 (0.06)<br>C: 0.94 (0.82)	

Subtypes

	BL	intermediate	DLBCL	FL/DLBCL	FL	other
BL		G: 0.77 (0.39)<br>R: --- [1]<br>C: 0.67 (0.43)	G: 0.63 (0.05)<br>R: --- [1]<br>**C: 0.37 (<0.01)**	G: 1.26 (0.59)<br>R: --- [1]<br>C: 0.84 (0.82)	G: 1.09 (0.77)<br>R: --- [1]<br>C: 0.77 (0.44)	G: 2.15 (0.10)<br>R: --- [1]<br>C: 0.34 (0.18)
inter-mediate	G: 1.31 (0.39)<br>R: --- [1]<br>C: 0.67 (0.43)		G: 0.65 (0.06)<br>R: 0.63 (0.05)<br>**C: 0.48 (<0.01)**	G: 1.67 (0.26)<br>R: 1.19 (0.74)<br>C: 1.02 (0.97)	G: 1.51 (0.16)<br>R: 0.54 (0.26)<br>C: 0.79 (0.44)	G: 2.21 (0.07)<br>R: 1.52 (0.52)<br>C: 0.70 (0.48)
DLBCL	G: 1.59 (0.05)<br>R: --- [1]<br>**C: 0.37 (<0.01)**	G: 1.54 (0.06)<br>R: 0.63 (0.05)<br>**C: 0.48 (<0.01)**		G: 2.10 (0.04)<br>R: 0.61 (0.04)<br>C: 0.49 (0.01)	**G: 2.79 (<0.01)**<br>R: 0.56 (0.02)<br>**C: 0.53 (<0.01)**	**G: 3.87 (<0.01)**<br>R: 0.59 (0.03)<br>**C: 0.45 (<0.01)**
FL/DLBCL	G: 0.8 (0.59)<br>R: --- [1]<br>C: 0.84 (0.82)	G: 0.6 (0.26)<br>R: 1.19 (0.74)<br>C: 1.02 (0.97)	G: 0.48 (0.04)<br>R: 0.61 (0.04)<br>C: 0.49 (0.01)		G: 1.28 (0.54)<br>R: 0.52 (0.24)<br>C: 1.11 (0.76)	G: 1.92 (0.27)<br>R: 1.13 (0.88)<br>C: 2.47 (0.38)
FL	G: 0.91 (0.77)<br>R: --- [1]<br>C: 0.77 (0.44)	G: 0.66 (0.16)<br>R: 0.54 (0.26)<br>C: 0.79 (0.44)	**G: 0.36 (<0.01)**<br>R: 0.56 (0.02)<br>**C: 0.53 (<0.01)**	G: 0.78 (0.54)<br>R: 0.52 (0.24)<br>C: 1.11 (0.76)		G: 1.98 (0.11)<br>R: 0.18 (0.10)<br>C: 0.91 (0.79)
other	G: 0.46 (0.1)<br>R: --- [1]<br>C: 0.34 (0.18)	G: 0.45 (0.07)<br>R: 1.52 (0.52)<br>C: 0.70 (0.48)	**G: 0.26 (<0.01)**<br>R: 0.59 (0.03)<br>**C: 0.45 (<0.01)**	G: 0.52 (0.27)<br>R: 1.13 (0.88)<br>C: 2.47 (0.38)	G: 0.5 (0.11)<br>R: 0.18 (0.10)<br>C: 0.91 (0.79)	

[1]: no data on BL cases with Rituximab treatment available

DLBCL ABC vs. GCB

	ABC	GCB
ABC		**G: 2.38 (< 0.01)**<br>R: 0.48 (0.02)<br>C: 0.70 (0.40)
GCB	**G: 0.42 (< 0.01)**<br>R: 0.48 (0.02)<br>C: 0.70 (0.40)	

BL in children vs. adults

	Child	Adult
Child		G: 0.40 (0.02)<br>R: --- [1]<br>C: --- [2]
Adult	G: 2.52 (0.02)<br>R: --- [1]<br>C: --- [2]	

[1]: no data on BL cases with Rituximab treatment
[2]: no data on BL cases without chemotherapy

PAT types

	AD	E	F	G	FIJ	I	L	H	IJ
AD		G: 0.56 (0.07)<br>R: 0.96 (0.94)<br>C: 0.37 (0.03)	G: 0.64 (0.11)<br>R: 0.83 (0.60)<br>C: 0.39 (0.03)	G: 1.25 (0.47)<br>R: 1.06 (0.91)<br>C: 0.30 (0.02)	G: 1.19 (0.61)<br>R: 1.81 (0.17)<br>C: 0.81 (0.73)	G: 2.03 (0.06)<br>R: 1.05 (0.93)<br>C: 0.58 (0.18)	G: 0.99 (0.98)<br>R: 1.26 (0.62)<br>C: 0.53 (0.31)	G: 0.42 (0.14)<br>R: --- [1]<br>C: 0.84 (0.80)	G: 0.88 (0.74)<br>R: 3.36 (0.04)<br>C: 1.65 (0.44)
E	G: 1.78 (0.07)<br>R: 0.96 (0.94)<br>C: 0.37 (0.03)		G: 1.06 (0.82)<br>R: 0.46 (0.06)<br>C: 0.44 (0.03)	G: 2.11 (0.01)<br>R: 0.31 (0.10)<br>C: 0.37 (0.02)	G: 2 .00 (0.03)<br>R: 0.57 (0.30)<br>C: 0.72 (0.50)	**G: 3.22 (<0.01)**<br>R: --- [1]<br>C: 0.68 (0.29)	G: 1.34 (0.44)<br>R: 0.36 (0.11)<br>C: 0.65 (0.38)	G: 0.78 (0.64)<br>R: --- [1]<br>C: 0.67 (0.45)	G: 2.01 (0.06)<br>R: --- [1]<br>C: 0.91 (0.85)
F	G: 1.57 (0.11)<br>R: 0.83 (0.60)<br>C: 0.39 (0.03)	G: 0.94 (0.82)<br>R: 0.46 (0.06)<br>C: 0.44 (0.03)		**G: 2.05 (<0.01)**<br>R: 0.58 (0.13)<br>C: 0.39 (0.02)	G: 1.91 (0.02)<br>R: 0.72 (0.33)<br>C: 0.66 (0.38)	**G: 3.5 (<0.01)**<br>R: 0.49 (0.09)<br>C: 0.61 (0.17)	G: 1.38 (0.35)<br>R: 0.55 (0.10)<br>C: 0.59 (0.28)	G: 0.77 (0.62)<br>R: --- [1]<br>C: 0.65 (0.38)	G: 1.7 (0.14)<br>R: 0.51 (0.11)<br>C: 0.86 (0.77)
G	G: 0.80 (0.47)<br>R: 1.06 (0.91)<br>C: 0.30 (0.02)	G: 0.47 (0.01)<br>R: 0.31 (0.10)<br>C: 0.37 (0.02)	**G: 0.49 (<0.01)**<br>R: 0.58 (0.13)<br>C: 0.39 (0.02)		G: 0.92 (0.79)<br>R: 0.90 (0.81)<br>C: 0.67 (0.46)	G: 1.75 (0.12)<br>R: 0.34 (0.15)<br>C: 0.56 (0.15)	G: 0.71 (0.36)<br>R: 0.58 (0.29)<br>C: 0.55 (0.28)	G: 0.32 (0.06)<br>R: --- [1]<br>C: 0.82 (0.76)	G: 0.87 (0.72)<br>R: 0.47 (0.31)<br>C: 0.94 (0.91)
FIJ	G: 0.84 (0.61)<br>R: 1.81 (0.17)<br>C: 0.81 (0.73)	G: 0.50 (0.03)<br>R: 0.57 (0.30)<br>C: 0.72 (0.50)	G: 0.52 (0.02)<br>R: 0.72 (0.33)<br>C: 0.66 (0.38)	G: 1.09 (0.79)<br>R: 0.90 (0.81)<br>C: 0.67 (0.46)		G: 1.77 (0.10)<br>R: 0.67 (0.46)<br>C: 0.93 (0.84)	G: 0.77 (0.50)<br>R: 0.78 (0.60)<br>C: 1.48 (0.60)	G: 0.25 (0.01)<br>R: --- [1]<br>C: 2.60 (0.23)	G: 0.79 (0.56)<br>R: 0.97 (0.96)<br>C: 2.56 (0.21)

I	G: 0.49 (0.06)<br>R: 1.05 (0.93)<br>C: 0.58 (0.18)	**G: 0.31 (<0.01)**<br>R: --- [1]<br>C: 0.68 (0.29)	**G: 0.29 (<0.01)**<br>R: 0.49 (0.09)<br>C: 0.61 (0.17)	G: 0.57 (0.12)<br>R: 0.34 (0.15)<br>C: 0.56 (0.15)	G: 0.57 (0.10)<br>R: 0.67 (0.46)<br>C: 0.93 (0.84)		G: 0.45 (0.04)<br>R: 0.40 (0.15)<br>C: 0.88 (0.75)	G: 0.17 (0.01)<br>R: --- [1]<br>C: 1.04 (0.92)	G: 0.74 (0.43)<br>R: --- [1]<br>C: 1.2 (0.64)
L	G: 1.01 (0.98)<br>R: 1.26 (0.62)<br>C: 0.53 (0.31)	G: 0.74 (0.44)<br>R: 0.36 (0.11)<br>C: 0.65 (0.38)	G: 0.72 (0.35)<br>R: 0.55 (0.10)<br>C: 0.59 (0.28)	G: 1.42 (0.36)<br>R: 0.58 (0.29)<br>C: 0.55 (0.28)	G: 1.30 (0.50)<br>R: 0.78 (0.60)<br>C: 1.48 (0.60)	G: 2.24 (0.04)<br>R: 0.40 (0.15)<br>C: 0.88 (0.75)		G: 0.38 (0.09)<br>R: --- [1]<br>C: 1.87 (0.43)	G: 1.28 (0.59)<br>R: 0.41 (0.19)<br>C: 2.13 (0.32)
H	G: 2.38 (0.14)<br>R: --- [1]<br>C: 0.84 (0.8)	G: 1.29 (0.64)<br>R: --- [1]<br>C: 0.67 (0.45)	G: 1.30 (0.62)<br>R: --- [1]<br>C: 0.65 (0.38)	G: 3.10 (0.06)<br>R: --- [1]<br>C: 0.82 (0.76)	G: 4.04 (0.01)<br>R: --- [1]<br>C: 2.60 (0.23)	G: 6.02 (0.01)<br>R--- [1]<br>C: 1.04 (0.92)	G: 2.62 (0.09)<br>R: --- [1]<br>C: 1.87 (0.43)		G: 4.23 (0.01)<br>R: --- [1]<br>C: 3.34 (0.12)
IJ	G: 1.14 (0.74)<br>R: 3.36 (0.04)<br>C: 1.65 (0.44)	G: 0.50 (0.06)<br>R: --- [1])<br>C: 0.91 (0.85)	G: 0.59 (0.14)<br>R: 0.51 (0.11)<br>C: 0.86 (0.77)	G: 1.15 (0.72)<br>R: 0.47 (0.31)<br>C: 0.94 (0.91)	G: 1.26 (0.56)<br>R: 0.97 (0.96)<br>C: 2.56 (0.21)	G: 1.35 (0.43)<br>R: --- [1]<br>C: 1.20 (0.64)	G: 0.78 (0.59)<br>R: 0.41 (0.19)<br>C: 2.13 (0.32)	G: 0.24 (0.01)<br>R: --- [1]<br>C: 3.34 (0.12)	

[1]: no sufficient data on cases with Rituximab treatment available

DLBCL related PATs

	DLBCL reference	DLBCL A	DLBCL E	DLBCL EF	DLBCL F	DLBCL FG	DLBCL G	DLBCL none
DLBCL reference		G: 0.51 (0.14)<br>R: 0.62 (0.05)<br>**C: 0.4 (<0.01)**	G: 0.86 (0.56)<br>R: 0.55 (0.02)<br>**C: 0.44 (<0.01)**	G: 1.73 (0.16)<br>R: 0.57 (0.02)<br>C: --- [1]	G: 0.65 (0.03)<br>R: 0.57 (0.01)<br>**C: 0.37 (<0.01)**	G: 2.39 (0.04)<br>R: 0.56 (0.02)<br>**C: 0.45 (<0.01)**	G: 1.48 (0.25)<br>R: --- [2]<br>C: --- [1]	G: 0.78 (0.23)<br>R: 0.56 (0.01)<br>**C: 0.47 (<0.01)**
DLBCL A	G: 1.96 (0.14)<br>R: 0.62 (0.05)<br>**C: 0.4 (<0.01)**		G: 1.66 (0.33)<br>R: 0.54 (0.54)<br>C: 0.41 (0.12)	G: 3.75 (0.04)<br>R: 0.93 (0.92)<br>C: --- [1]	G: 1.27 (0.63)<br>R: 0.69 (0.42)<br>**C: 0.13 (<0.01)**	G: 4.24 (0.02)<br>R: 0.50 (0.52)<br>C: 0.47 (0.49)	G: 3.38 (0.03)<br>R: --- [2]<br>C: --- [1]	G: 1.55 (0.37)<br>R: 0.56 (0.28)<br>C: 0.54 (0.21)
DLBCL E	G: 1.16 (0.56)<br>R: 0.55 (0.02)<br>**C: 0.44 (<0.01)**	G: 0.6 (0.33)<br>R: 0.54 (0.54)<br>C: 0.41 (0.12)		G: 2.02 (0.13)<br>R: 0.38 (0.21)<br>C: --- [1]	G: 0.69 (0.24)<br>R: 0.43 (0.08)<br>**C: 0.32 (<0.01)**	G: 2.46 (0.06)<br>R: --- [2]<br>C: 0.79 (0.71)	G: 2.00 (0.08)<br>R: --- [2]<br>C: --- [1])	G: 0.9 (0.72)<br>R: 0.33 (0.07)<br>C: 0.65 (0.30)

DLBCL EF	G: 0.58<br>(0.16)<br>R: 0.57<br>(0.02)<br>C: ---[1]	G: 0.27<br>(0.04)<br>R: 0.93<br>(0.92)<br>C: ---[1]	G: 0.50<br>(0.13)<br>R: 0.38<br>(0.21)<br>C: ---[1]		G: 0.40<br>(0.03)<br>R: 0.51<br>(0.11)<br>C: ---[1]	G: 1.40<br>(0.55)<br>R: 0.35<br>(0.18)<br>C: ---[1]	G: 0.98<br>(0.96)<br>R: ---[2]<br>C: ---[1]	G: 0.45<br>(0.06)<br>R: 0.50<br>(0.15)<br>C: ---[1]
DLBCL F	G: 1.55<br>(0.03)<br>R: 0.57<br>(0.01)<br>**C: 0.37**<br>**(<0.01)**	G: 0.79<br>(0.63)<br>R: 0.69<br>(0.42)<br>**C: 0.13**<br>**(<0.01)**	G: 1.45<br>(0.24)<br>R: 0.43<br>(0.08)<br>**C: 0.32**<br>**(<0.01)**	G: 2.53<br>(0.03)<br>R: 0.51<br>(0.11)<br>C: ---[1]		**G: 4.16**<br>**(<0.01)**<br>R: 0.45<br>(0.10)<br>C: 0.24<br>(0.01)	G: 2.1<br>(0.05)<br>R: ---[2]<br>C: ---[1]	G: 1.25<br>(0.40)<br>R: 0.48<br>(0.06)<br>C: 0.41<br>(0.02)
DLBCL FG	G: 0.42<br>(0.04)<br>R: 0.56<br>(0.02)<br>**C: 0.45**<br>**(<0.01)**	G: 0.24<br>(0.02)<br>R: 0.50<br>(0.52)<br>C: 0.47<br>(0.49)	G: 0.41<br>(0.06)<br>R: ---[2]<br>C: 0.79<br>(0.71)	G: 0.71<br>(0.55)<br>R: 0.35<br>(0.18)<br>C: ---[1]	**G: 0.24**<br>**(<0.01)**<br>R: 0.45<br>(0.10)<br>C: 0.24<br>(0.01)		G: 0.65<br>(0.37)<br>R: ---[2]<br>C: ---[1]	G: 0.32<br>(0.01)<br>R: 0.35<br>(0.08)<br>C: 0.87<br>(0.79)
DLBCL G	G: 0.68<br>(0.25)<br>R: ---[2]<br>C: ---[1]	G: 0.30<br>(0.03)<br>R: ---[2]<br>C: ---[1]	G: 0.5<br>(0.08)<br>R: ---[2]<br>C: ---[1]	G: 1.02<br>(0.96)<br>R: ---[2]<br>C: ---[1]	G: 0.48<br>(0.05)<br>R: ---[2]<br>C: ---[1]	G: 1.54<br>(0.37)<br>R: ---[2]<br>C: ---[1]		G: 0.43<br>(0.03)<br>R: ---[2]<br>C: ---[1]
DLBCL none	G: 1.28<br>(0.23)<br>R: 0.56<br>(0.01)<br>**C: 0.47**<br>**(<0.01)**	G: 0.64<br>(0.37)<br>R: 0.56<br>(0.28)<br>C: 0.54<br>(0.21)	G: 1.12<br>(0.72)<br>R: 0.33<br>(0.07)<br>C: 0.65<br>(0.30)	G: 2.22<br>(0.06)<br>R: 0.50<br>(0.15)<br>C: ---[1]	G: 0.80<br>(0.40)<br>R: 0.48<br>(0.06)<br>C: 0.41<br>(0.02)	G: 3.14<br>(0.01)<br>R: 0.35<br>(0.08)<br>C: 0.87<br>(0.79)	G: 2.31<br>(0.03)<br>R: ---[2]<br>C: ---[1]	

[1]: no sufficient data on cases without chemotherapy available

[2]: no sufficient data on cases with Rituximab treatment available

5 References

1. Binder H, Preibisch S: **“Hook”-calibration of GeneChip-microarrays: theory and algorithm.** *Algorithms Mol Biol* 2008, **3**:12.

2. Binder H, Krohn K, Preibisch S: **“Hook”-calibration of GeneChip-microarrays: chip characteristics and expression measures.** *Algorithms Mol Biol* 2008, **3**:11.

3. Bolstad BM, Irizarry RA, Astrand M, Speed TP: **A comparison of normalization methods for high density oligonucleotide array data based on variance and bias.** *Bioinformatics* 2003, **19**:185–93.

4. Hopp L, Wirth H, Fasold M, Binder H: **Portraying the expression landscapes of cancer subtypes: A glioblastoma multiforme and prostate cancer case study**. *Syst Biomed* 2013, **1**:1–23.

5. Hopp L, Lembcke K, Binder H, Wirth H: **Portraying the Expression Landscapes of B-Cell Lymphoma - Intuitive Detection of Outlier Samples and of Molecular Subtypes.** *Biology (Basel)* 2013, **2**:1411–1437.

6. Kohonen T: **Self Organizing Maps**. *Springer, Berlin, Heidelberg, New York* 1995.

7. Wirth H: **Analysis of large-scale molecular biological data using self-organizing maps**. Leipzig University; 2012.

8. Saitou N, Nei M: **The neighbor-joining method: a new method for reconstructing phylogenetic trees.** *Mol Biol Evol* 1987, **4**:406–25.

9. Paradis E, Claude J, Strimmer K: **APE: Analyses of Phylogenetics and Evolution in R language.** *Bioinformatics* 2004, **20**:289–90.

10. Wirth H, Löffler M, von Bergen M, Binder H: **Expression cartography of human tissues using self organizing maps**. *BMC Bioinformatics* 2011, **12**:306–352.

11. Wirth H, von Bergen M, Murugaiyan J, Rösler U, Stokowy T, Binder H: **MALDI-typing of infectious algae of the genus Prototheca using SOM portraits.** *J Microbiol Methods* 2012, **88**:83–97.

12. Guo Y, Eichler GS, Feng Y, Ingber DE, Huang S: **Towards a holistic, yet gene-centered analysis of gene expression profiles: a case study of human lung cancers.** *J Biomed Biotechnol* 2006, **2006**:69141.

13. Wirth H, von Bergen M, Binder H: **Mining SOM expression portraits: feature selection and integrating concepts of molecular function.** *BioData Min* 2012, **5**:18–63.

14. Cakir MV, Wirth H, Hopp L, Binder H: **MicroRNA Expression Landscapes in Stem Cells, Tissues, and Cancer.** *Methods Mol Biol* 2014, **1107**:279–302.

15. Quackenbush J: **Genomics. Microarrays--guilt by association.** *Science* 2003, **302**:240–1.

16. Ashburner M, Ball CA, Blake JA, Botstein D, Butler H, Cherry JM, Davis AP, Dolinski K, Dwight SS, Eppig JT, Harris MA, Hill DP, Issel-Tarver L, Kasarskis A, Lewis S, Matese JC, Richardson JE, Ringwald M, Rubin GM, Sherlock G: **Gene ontology: tool for the unification of biology. The Gene Ontology Consortium.** *Nat Genet* 2000, **25**:25–9.

17. Haider S, Ballester B, Smedley D, Zhang J, Rice P, Kasprzyk A: **BioMart Central Portal--unified access to biological data.** *Nucleic Acids Res* 2009, **37**(Web Server issue):W23-7.

18. Zhang B, Kirov S, Snoddy J: **WebGestalt: an integrated system for exploring gene sets in various biological contexts.** *Nucleic Acids Res* 2005, **33**(Web Server issue):W741-8.

19. Vêncio RZN, Shmulevich I: **ProbCD: enrichment analysis accounting for categorization uncertainty.** *BMC Bioinformatics* 2007, **8**:383.

20. Törönen P, Ojala PJ, Marttinen P, Holm L: **Robust extraction of functional signals from gene set analysis using a generalized threshold free scoring function.** *BMC Bioinformatics* 2009, **10**:307.

21. Drexler HG: **Establishment and Culture of Leukemia–Lymphoma Cell Lines**. In *Methods Mol Biol*. *Volume 731*; 2011:181–200.

22. Hummel M, Bentink S, Berger H, Klapper W, Wessendorf S, Barth TFE, Bernd H-W, Cogliatti SB, Dierlamm J, Feller AC, Hansmann M-L, Haralambieva E, Harder L, Hasenclever D, Kühn M, Lenze D, Lichter P, Martin-Subero JI, Möller P, Müller-Hermelink H-K, Ott G, Parwaresch RM, Pott C, Rosenwald A, Rosolowski M, Schwaenen C, Stürzenhofecker B, Szczepanowski M, Trautmann H, Wacker H-H, et al.: **A biologic definition of Burkitt's lymphoma from transcriptional and genomic profiling.** *N Engl J Med* 2006, **354**:2419–30.

23. Ott G, Ziepert M, Klapper W, Horn H, Szczepanowski M, Bernd H-W, Thorns C, Feller AC, Lenze D, Hummel M, Stein H, Muller-Hermelink H-K, Frank M, Hansmann M-L, Barth TFE, Moller P, Cogliatti S, Pfreundschuh M, Schmitz N, Trumper L, Loeffler M, Rosenwald A: **Immunoblastic morphology but not the immunohistochemical GCB/nonGCB classifier predicts outcome in diffuse large B-cell lymphoma in the RICOVER-60 trial of the DSHNHL**. *Blood* 2010, **116**:4916–4925.

24. Feller A, Diebold J: *Histopathology of Nodal and Extranodal Non-Hodgkin's Lymphomas (Based on the WHO Classification)*. New York: Springer; 2004.

25. Lennert K, Feller A: *Histopathology of Non-Hodgkin's Lymphomas*. New York: Springer; 1992.

26. Wilkinson ST, Vanpatten KA, Fernandez DR, Brunhoeber P, Garsha KE, Glinsmann-Gibson BJ, Grogan TM, Teruya-Feldstein J, Rimsza LM: **Partial plasma cell differentiation as a mechanism of lost major histocompatibility complex class II expression in diffuse large B-cell lymphoma.** *Blood* 2012, **119**:1459–67.

27. Aukema SM, Kreuz M, Kohler CW, Rosolowski M, Hasenclever D, Hummel M, Küppers R, Lenze D, Ott G, Pott C, Richter J, Rosenwald A, Szczepanowski M, Schwaenen C, Stein H, Trautmann H, Wessendorf S, Trümper L, Loeffler M, Spang R, Kluin PM, Klapper W, Siebert R: **Biological characterization of adult MYC-translocation-positive mature B-cell lymphomas other than molecular Burkitt lymphoma.** *Haematologica* 2014, **99**:726–35.

28. Ennishi D, Jiang A, Boyle M, Collinge B, Grande BM, Ben-Neriah S, Rushton C, Tang J, Thomas N, Slack GW, Farinha P, Takata K, Miyata-Takata T, Craig J, Mottok A, Meissner B, Saberi S, Bashashati A, Villa D, Savage KJ, Sehn LH, Kridel R, Mungall AJ, Marra MA, Shah SP, Steidl C, Connors JM, Gascoyne RD, Morin RD, Scott DW: **Double-Hit Gene Expression Signature Defines a Distinct Subgroup of Germinal Center B-Cell-Like Diffuse Large B-Cell Lymphoma**. *J Clin Oncol* 2019, **37**:190–201.

29. Lenz G, Wright G, Dave SS, Xiao W, Powell J, Zhao H, Xu W, Tan B, Goldschmidt N, Iqbal J, Vose J, Bast M, Fu K, Weisenburger DD, Greiner TC, Armitage JO, Kyle A, May L, Gascoyne RD, Connors JM, Troen G, Holte H, Kvaloy S, Dierickx D, Verhoef G, Delabie J, Smeland EB, Jares P, Martinez A, Lopez-Guillermo A, et al.: **Stromal gene signatures in large-B-cell lymphomas.** *N Engl J Med* 2008, **359**:2313–23.

30. Wright G, Tan B, Rosenwald A, Hurt EH, Wiestner A, Staudt LM: **A gene expression-based method to diagnose clinically distinct subgroups of diffuse large B cell lymphoma.** *Proc Natl Acad Sci U S A* 2003, **100**:9991–6.

31. Dave SS, Wright G, Tan B, Rosenwald A, Gascoyne RD, Chan WC, Fisher RI, Braziel RM, Rimsza LM, Grogan TM, Miller TP, LeBlanc M, Greiner TC, Weisenburger DD, Lynch JC, Vose J, Armitage JO, Smeland EB, Kvaloy S, Holte H, Delabie J, Connors JM, Lansdorp PM,

Ouyang Q, Lister TA, Davies AJ, Norton AJ, Muller-Hermelink HK, Ott G, Campo E, et al.: **Prediction of survival in follicular lymphoma based on molecular features of tumor-infiltrating immune cells.** *N Engl J Med* 2004, **351**:2159–69.

32. Hebenstreit D, Fang M, Gu M, Charoensawan V, van Oudenaarden A, Teichmann SA: **RNA sequencing reveals two major classes of gene expression levels in metazoan cells.** *Mol Syst Biol* 2011, **7**:497.

33. Tang X, Milyavsky M, Goldfinger N, Rotter V: **Amyloid-beta precursor-like protein APLP1 is a novel p53 transcriptional target gene that augments neuroblastoma cell death upon genotoxic stress.** *Oncogene* 2007, **26**:7302–12.

34. Gustafson AM, Soldi R, Anderlind C, Scholand MB, Qian J, Zhang X, Cooper K, Walker D, McWilliams A, Liu G, Szabo E, Brody J, Massion PP, Lenburg ME, Lam S, Bild AH, Spira A: **Airway PI3K pathway activation is an early and reversible event in lung cancer development.** *Sci Transl Med* 2010, **2**:26ra25.

35. Schrader A, Bentink S, Spang R, Lenze D, Hummel M, Kuo M, Arrand JR, Murray PG, Trümper L, Kube D, Vockerodt M: **High Myc activity is an independent negative prognostic factor for diffuse large B cell lymphomas.** *Int J Cancer* 2012, **131**:E348-61.

36. Gilmore TD: **Introduction to NF-kappaB: players, pathways, perspectives.** *Oncogene* 2006, **25**:6680–4.

37. Tarte K, Zhan F, De Vos J, Klein B, Shaughnessy J: **Gene expression profiling of plasma cells and plasmablasts: toward a better understanding of the late stages of B-cell differentiation.** *Blood* 2003, **102**:592–600.

38. Victora GD, Dominguez-Sola D, Holmes AB, Deroubaix S, Dalla-Favera R, Nussenzweig MC: **Identification of human germinal center light and dark zone cells and their relationship to human B-cell lymphomas.** *Blood* 2012, **120**:2240–8.

39. Basso K, Klein U, Niu H, Stolovitzky GA, Tu Y, Califano A, Cattoretti G, Dalla-Favera R: **Tracking CD40 signaling during germinal center development.** *Blood* 2004, **104**:4088–96.

40. Angelova M, Charoentong P, Hackl H, Fischer ML, Snajder R, Krogsdam AM, Waldner MJ, Bindea G, Mlecnik B, Galon J, Trajanoski Z: **Characterization of the immunophenotypes and antigenomes of colorectal cancers reveals distinct tumor escape mechanisms and novel targets for immunotherapy**. *Genome Biol* 2015, **16**:64.

41. Schmitz R, Wright GW, Huang DW, Johnson CA, Phelan JD, Wang JQ, Roulland S, Kasbekar M, Young RM, Shaffer AL, Hodson DJ, Xiao W, Yu X, Yang Y, Zhao H, Xu W, Liu X, Zhou B, Du W, Chan WC, Jaffe ES, Gascoyne RD, Connors JM, Campo E, Lopez-Guillermo A, Rosenwald A, Ott G, Delabie J, Rimsza LM, Tay Kuang Wei K, et al.: **Genetics and Pathogenesis of Diffuse Large B-Cell Lymphoma**. *N Engl J Med* 2018, **378**:1396–1407.

42. Chapuy B, Stewart C, Dunford AJ, Kim J, Kamburov A, Redd RA, Lawrence MS, Roemer MGM, Li AJ, Ziepert M, Staiger AM, Wala JA, Ducar MD, Leshchiner I, Rheinbay E, Taylor-Weiner A, Coughlin CA, Hess JM, Pedamallu CS, Livitz D, Rosebrock D, Rosenberg M, Tracy AA, Horn H, van Hummelen P, Feldman AL, Link BK, Novak AJ, Cerhan JR, Habermann TM, et al.: **Molecular subtypes of diffuse large B cell lymphoma are associated with distinct pathogenic mechanisms and outcomes**. *Nat Med* 2018, **24**:679–690.

43. Nersisyan L, Löffler-Wirth H, Arakelyan A, Binder H: **Gene Set-and Pathway-Centered Knowledge Discovery Assigns Transcriptional Activation Patterns in Brain, Blood, and Colon Cancer: A Bioinformatics Perspective**. *Int J Knowl Discov Bioinforma* 2016, **4**.

44. Staudt LM, Dave S: **The biology of human lymphoid malignancies revealed by gene expression profiling.** *Adv Immunol* 2005, **87**:163–208.

45. Cerhan JR, Berndt SI, Vijai J, Ghesquières H, McKay J, Wang SS, Wang Z, Yeager M, Conde L, de Bakker PIW, Nieters A, Cox D, Burdett L, Monnereau A, Flowers CR, De Roos AJ, Brooks-Wilson AR, Lan Q, Severi G, Melbye M, Gu J, Jackson RD, Kane E, Teras LR, Purdue MP, Vajdic CM, Spinelli JJ, Giles GG, Albanes D, Kelly RS, et al.: **Genome-wide association study identifies multiple susceptibility loci for diffuse large B cell lymphoma.** *Nat Genet* 2014, **46**:1233–8.

46. Skibola CF, Berndt SI, Vijai J, Conde L, Wang Z, Yeager M, de Bakker PIW, Birmann BM, Vajdic CM, Foo J-N, Bracci PM, Vermeulen RCH, Slager SL, de Sanjose S, Wang SS, Linet MS, Salles G, Lan Q, Severi G, Hjalgrim H, Lightfoot T, Melbye M, Gu J, Ghesquières H, Link BK, Morton LM, Holly EA, Smith A, Tinker LF, Teras LR, et al.: **Genome-wide association study identifies five susceptibility loci for follicular lymphoma outside the HLA region.** *Am J Hum Genet* 2014, **95**:462–71.

47. Hanahan D, Weinberg RA: **Hallmarks of cancer: the next generation.** *Cell* 2011, **144**:646–74.

48. Ritz O, Rommel K, Dorsch K, Kelsch E, Melzner J, Buck M, Leroy K, Papadopoulou V, Wagner S, Marienfeld R, Brüderlein S, Lennerz JK, Möller P: **STAT6-mediated BCL6 repression in primary mediastinal B-cell lymphoma (PMBL).** *Oncotarget* 2013, **4**:1093–102.

49. Quan TE, Roman RM, Rudenga BJ, Holers VM, Craft JE: **Epstein-Barr virus promotes interferon-alpha production by plasmacytoid dendritic cells.** *Arthritis Rheum* 2010, **62**:1693–701.

50. Piccaluga PP, De Falco G, Kustagi M, Gazzola A, Agostinelli C, Tripodo C, Leucci E, Onnis A, Astolfi A, Sapienza MR, Bellan C, Lazzi S, Tumwine L, Mawanda M, Ogwang M, Calbi V, Formica S, Califano A, Pileri SA, Leoncini L: **Gene expression analysis uncovers similarity and differences among Burkitt lymphoma subtypes.** *Blood* 2011, **117**:3596–608.

51. Drexler H: *The Leukemia-Lymphoma Cell Line Factsbook*. Academic Press; 2001.

52. Salaverria I, Philipp C, Oschlies I, Kohler CW, Kreuz M, Szczepanowski M, Burkhardt B, Trautmann H, Gesk S, Andrusiewicz M, Berger H, Fey M, Harder L, Hasenclever D, Hummel M, Loeffler M, Mahn F, Martin-Guerrero I, Pellissery S, Pott C, Pfreundschuh M, Reiter A, Richter J, Rosolowski M, Schwaenen C, Stein H, Trümper L, Wessendorf S, Spang R, Küppers R, et al.: **Translocations activating IRF4 identify a subtype of germinal center-derived B-cell lymphoma affecting predominantly children and young adults.** *Blood* 2011, **118**:139–47.

53. Salaverria I, Martin-Guerrero I, Wagener R, Kreuz M, Kohler CW, Richter J, Pienkowska-Grela B, Adam P, Burkhardt B, Claviez A, Damm-Welk C, Drexler HG, Hummel M, Jaffe ES, Küppers R, Lefebvre C, Lisfeld J, Löffler M, Macleod RAF, Nagel I, Oschlies I, Rosolowski M, Russell RB, Rymkiewicz G, Schindler D, Schlesner M, Scholtysik R, Schwaenen C, Spang R, Szczepanowski M, et al.: **A recurrent 11q aberration pattern characterizes a subset of MYC-negative high-grade B-cell lymphomas resembling Burkitt lymphoma.** *Blood* 2014, **123**:1187–98.

54. Rosolowski M, Läuter J, Abramov D, Drexler H, Hummel M, Klapper W, MacLeod R, Pellissery S, Horn F, Siebert R, Löffler M: **Diffuse large B-cell lymphomas exhibit different functional and metabolic activation patterns independent of the cell of origin signature**. *PLoS One* 2013, **8**:1–12.

55. Bentink S, Wessendorf S, Schwaenen C, Rosolowski M, Klapper W, Rosenwald A, Ott G, Banham AH, Berger H, Feller AC, Hansmann M-L, Hasenclever D, Hummel M, Lenze D, Möller P, Stuerzenhofecker B, Loeffler M, Truemper L, Stein H, Siebert R, Spang R: **Pathway**

activation patterns in diffuse large B-cell lymphomas. *Leukemia* 2008, **22**:1746–54.

56. Klapper W, Kreuz M, Kohler CW, Burkhardt B, Szczepanowski M, Salaverria I, Hummel M, Loeffler M, Pellissery S, Woessmann W, Schwänen C, Trümper L, Wessendorf S, Spang R, Hasenclever D, Siebert R: **Patient age at diagnosis is associated with the molecular characteristics of diffuse large B-cell lymphoma.** *Blood* 2012, **119**:1882–7.

57. Alonso SR, Tracey L, Ortiz P, Pérez-Gómez B, Palacios J, Pollán M, Linares J, Serrano S, Sáez-Castillo AI, Sánchez L, Pajares R, Sánchez-Aguilera A, Artiga MJ, Piris MA, Rodríguez-Peralto JL: **A high-throughput study in melanoma identifies epithelial-mesenchymal transition as a major determinant of metastasis.** *Cancer Res* 2007, **67**:3450–60.

58. Bidus MA, Risinger JI, Chandramouli GVR, Dainty LA, Litzi TJ, Berchuck A, Barrett JC, Maxwell GL: **Prediction of lymph node metastasis in patients with endometrioid endometrial cancer using expression microarray.** *Clin Cancer Res* 2006, **12**:83–8.

59. Cromer A, Carles A, Millon R, Ganguli G, Chalmel F, Lemaire F, Young J, Dembélé D, Thibault C, Muller D, Poch O, Abecassis J, Wasylyk B: **Identification of genes associated with tumorigenesis and metastatic potential of hypopharyngeal cancer by microarray analysis.** *Oncogene* 2004, **23**:2484–98.

60. Jaeger J, Koczan D, Thiesen H-J, Ibrahim SM, Gross G, Spang R, Kunz M: **Gene expression signatures for tumor progression, tumor subtype, and tumor thickness in laser-microdissected melanoma tissues.** *Clin Cancer Res* 2007, **13**:806–15.

61. O’Donnell RK, Kupferman M, Wei SJ, Singhal S, Weber R, O’Malley B, Cheng Y, Putt M, Feldman M, Ziober B, Muschel RJ: **Gene expression signature predicts lymphatic metastasis in squamous cell carcinoma of the oral cavity.** *Oncogene* 2005, **24**:1244–51.

62. Ramaswamy S, Ross KN, Lander ES, Golub TR: **A molecular signature of metastasis in primary solid tumors.** *Nat Genet* 2003, **33**:49–54.

63. Vicent S, Luis-Ravelo D, Antón I, García-Tuñón I, Borrás-Cuesta F, Dotor J, De Las Rivas J, Lecanda F: **A novel lung cancer signature mediates metastatic bone colonization by a dual mechanism.** *Cancer Res* 2008, **68**:2275–85.

64. Croft D, Mundo AF, Haw R, Milacic M, Weiser J, Wu G, Caudy M, Garapati P, Gillespie M, Kamdar MR, Jassal B, Jupe S, Matthews L, May B, Palatnik S, Rothfels K, Shamovsky V, Song H, Williams M, Birney E, Hermjakob H, Stein L, D’Eustachio P: **The Reactome pathway knowledgebase.** *Nucleic Acids Res* 2014, **42**:D472-7.

65. Dairkee SH, Nicolau M, Sayeed A, Champion S, Ji Y, Moore DH, Yong B, Meng Z, Jeffrey SS: **Oxidative stress pathways highlighted in tumor cell immortalization: association with breast cancer outcome.** *Oncogene* 2007, **26**:6269–79.

66. Kang SK, Putnam L, Dufour J, Ylostalo J, Jung JS, Bunnell BA: **Expression of telomerase extends the lifespan and enhances osteogenic differentiation of adipose tissue-derived stromal cells.** *Stem Cells* 2004, **22**:1356–72.

50. Lenz G, Wright G, Dave SS, Xiao W, Powell J, Zhao H, Xu W, Tan B, Goldschmidt N, Iqbal J, Vose J, Bast M, Fu K, Weisenburger DD, Greiner TC, Armitage JO, Kyle A, May L, Gascoyne RD, Connors JM, Troen G, Holte H, Kvaloy S, Dierickx D, Verhoef G, Delabie J, Smeland EB, Jares P, Martinez A, Lopez-Guillermo A, et al.: **Stromal gene signatures in large-B-cell lymphomas.** *N Engl J Med* 2008, **359**:2313–23.

51. Schrader A, Meyer K, von Bonin F, Vockerodt M, Walther N, Hand E, Ulrich A, Matulewicz K, Lenze D, Hummel M, Kieser A, Engelke M, Trümper L, Kube D: **Global gene expression changes of in vitro stimulated human transformed germinal centre B cells as**

surrogate for oncogenic pathway activation in individual aggressive B cell lymphomas. *Cell Commun Signal* 2012, 10:43.
