# Supplementary figures and images for: A modular transcriptome map of mature B cell lymphomas
Source: Genome Med. 2019 Apr 30;11:27. doi: 10.1186/s13073-019-0637-7 (PMC6492344; doi:10.1186/s13073-019-0637-7)

intermediate

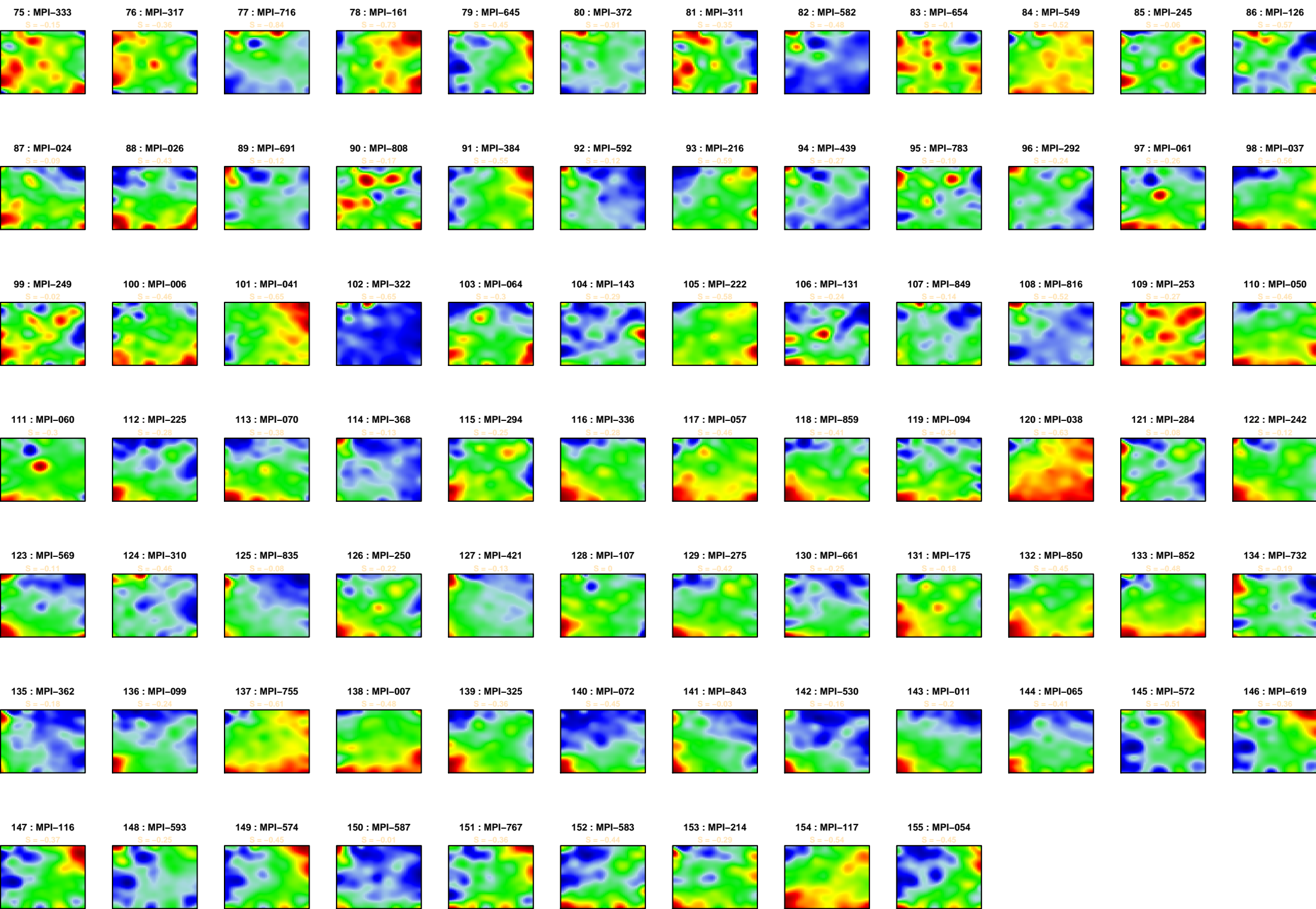

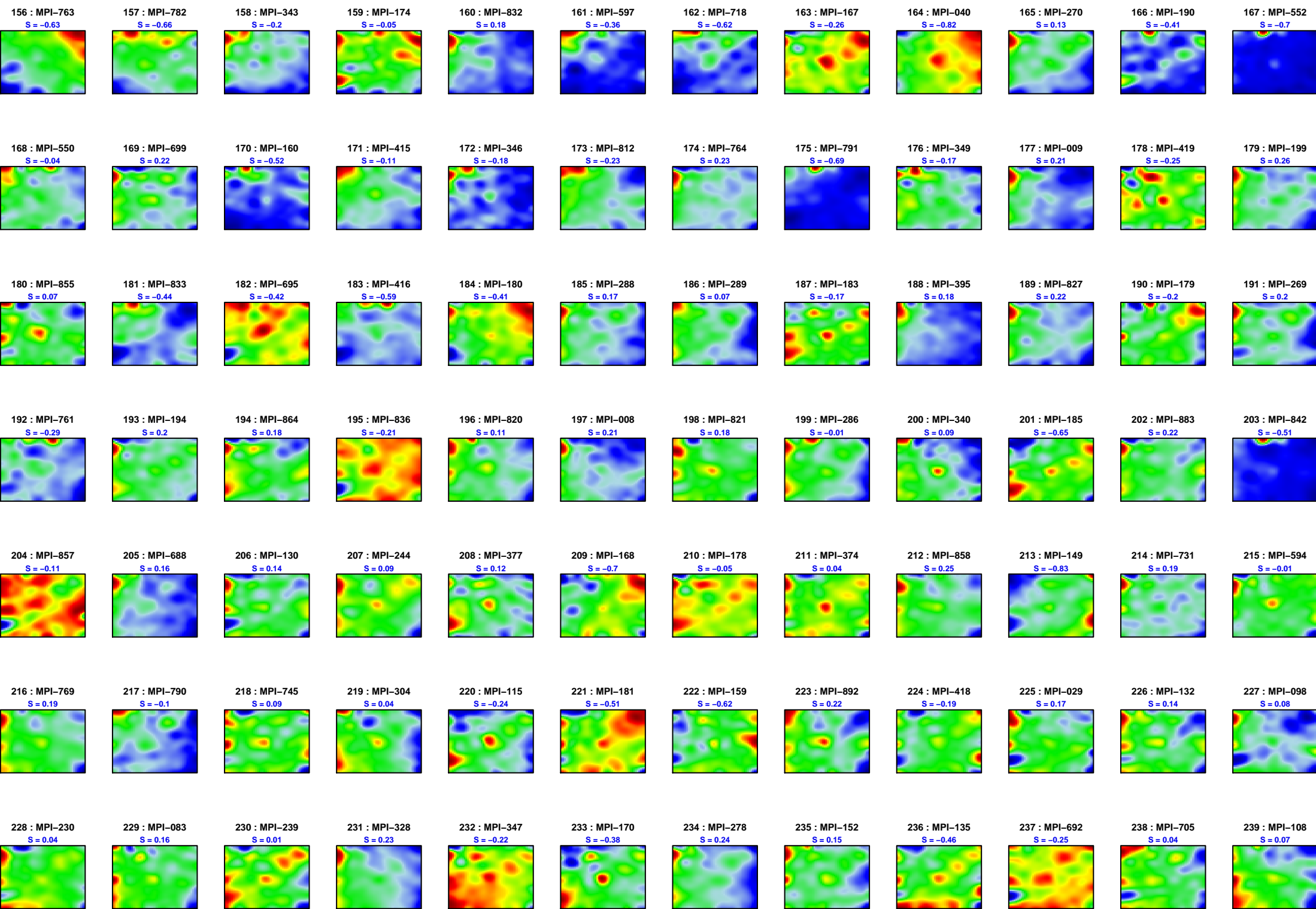

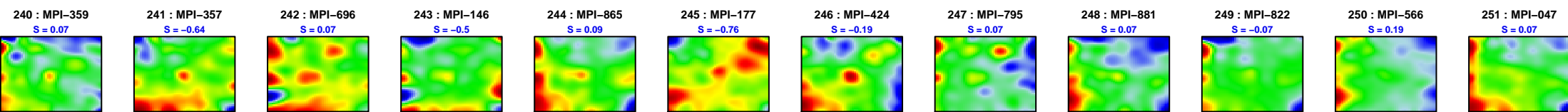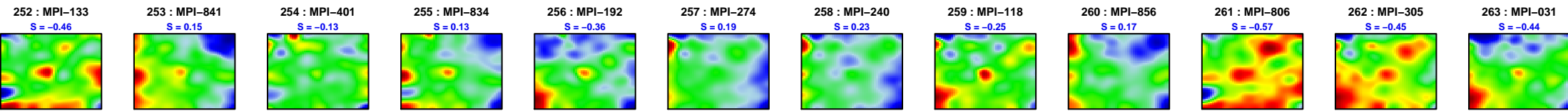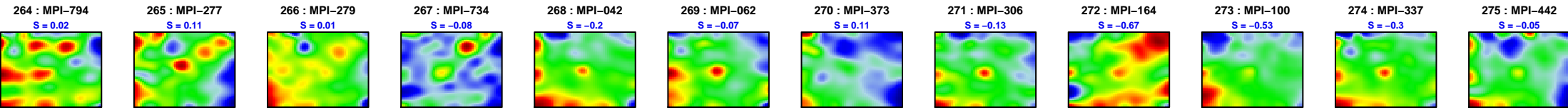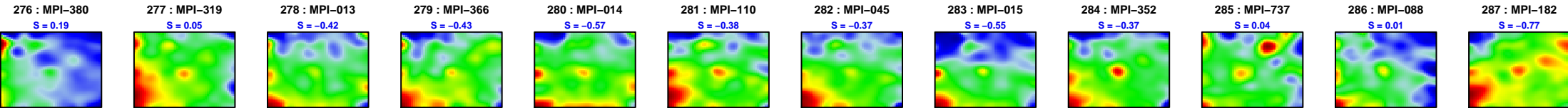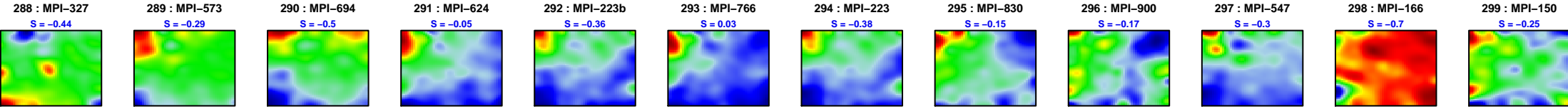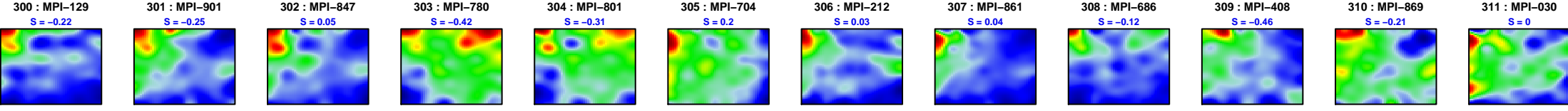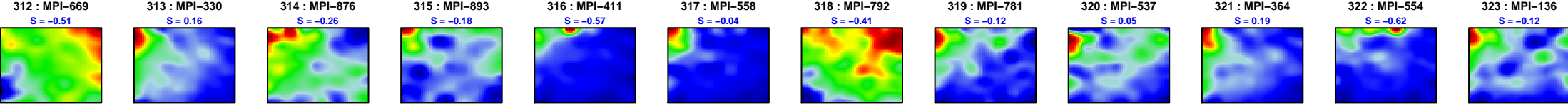

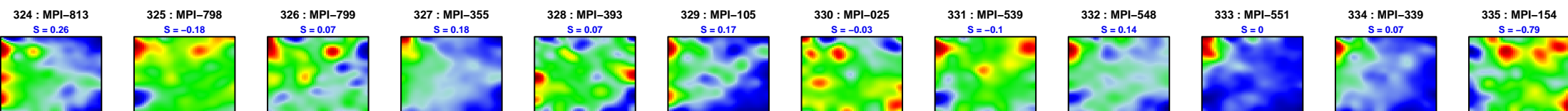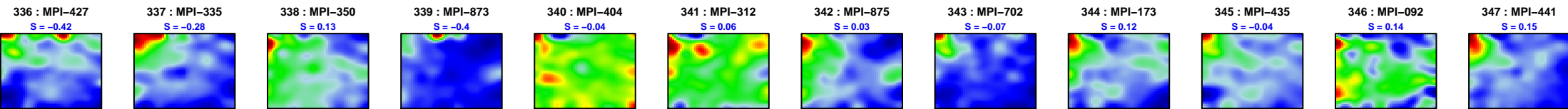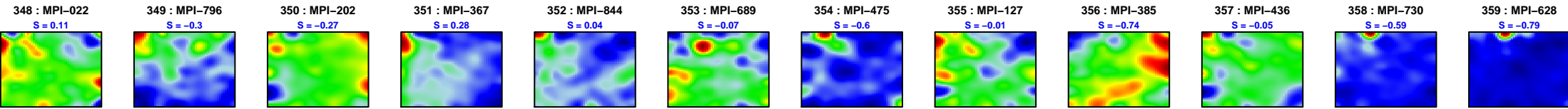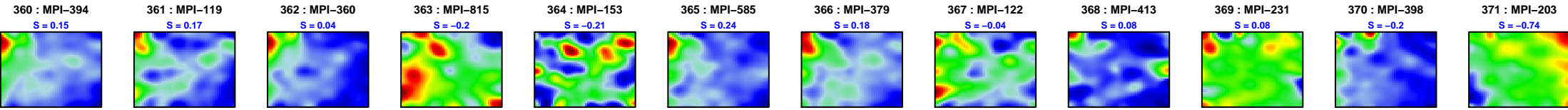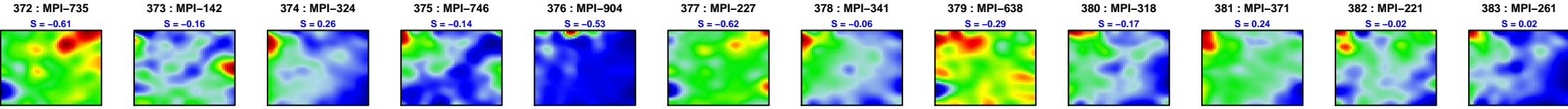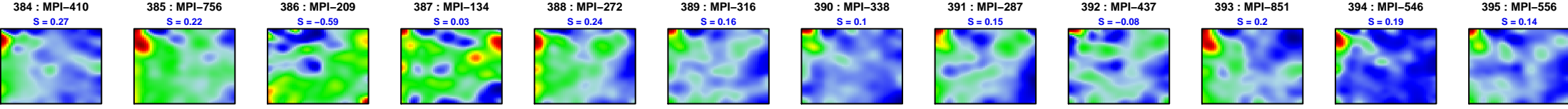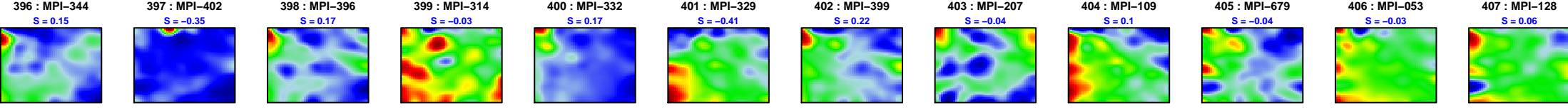

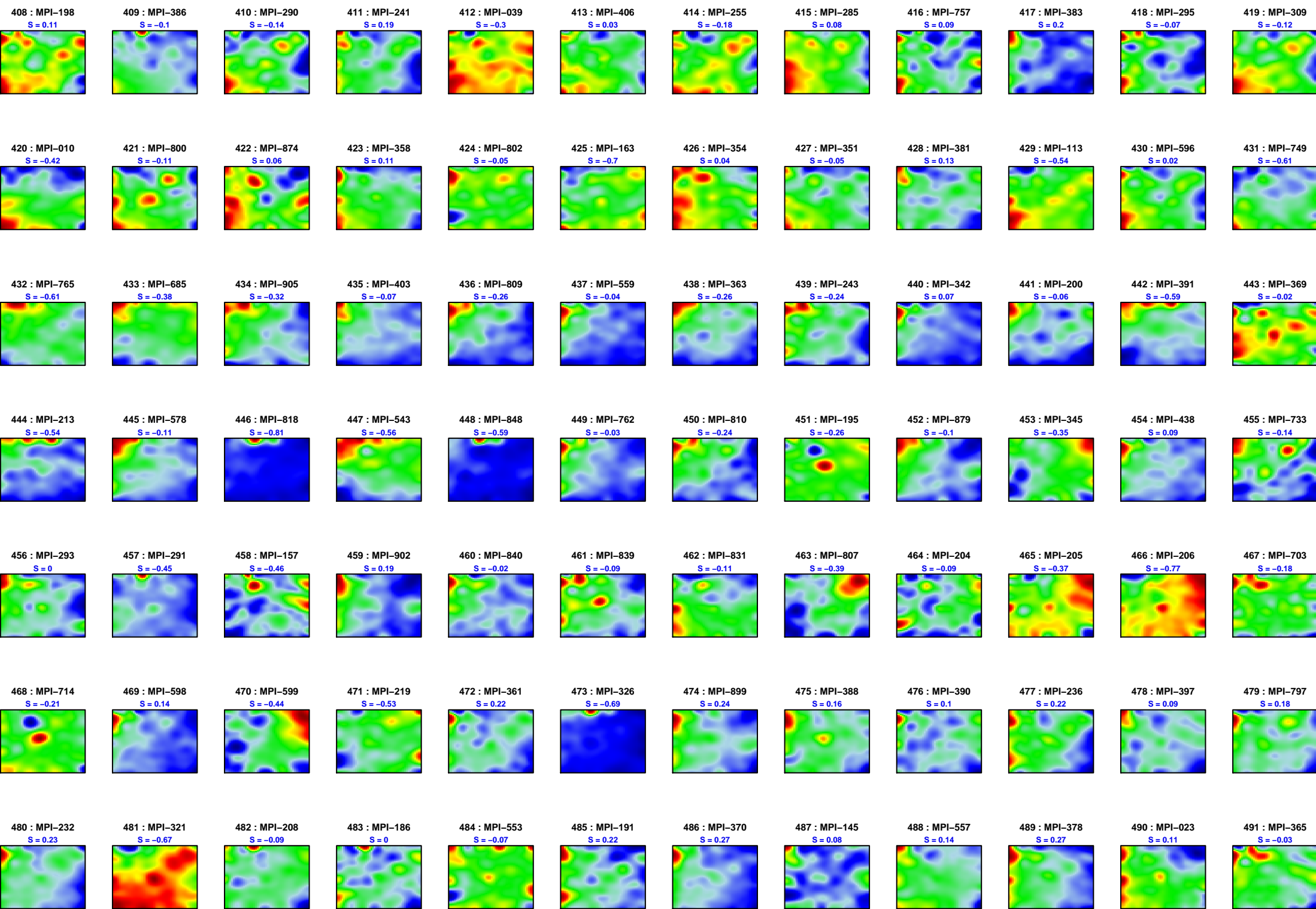

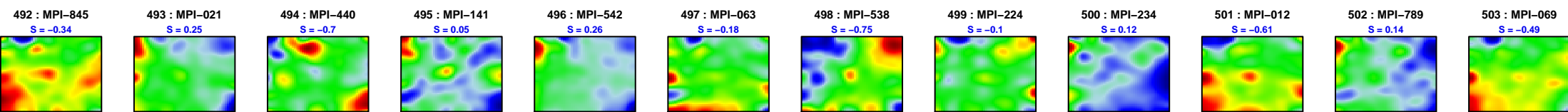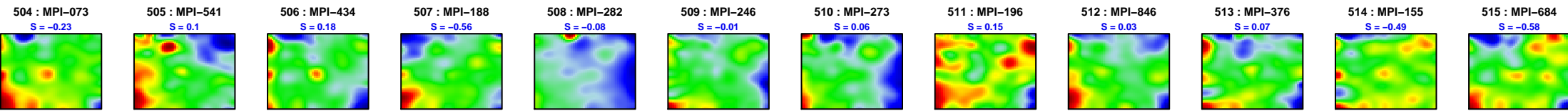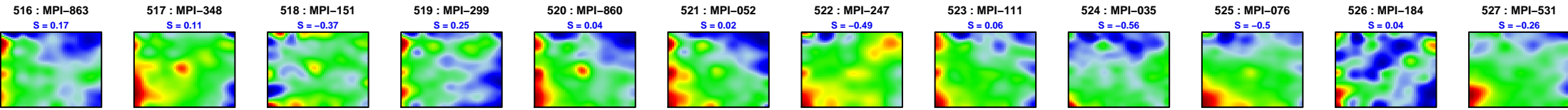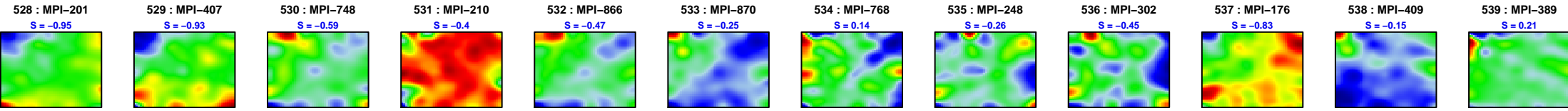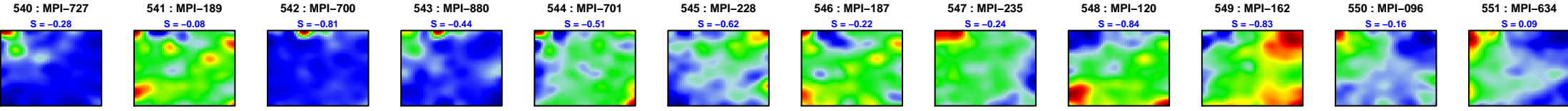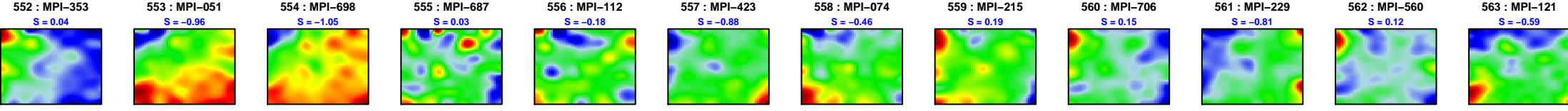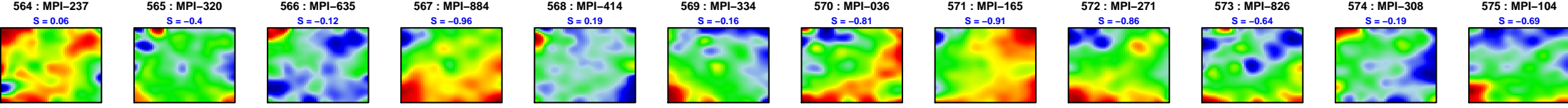

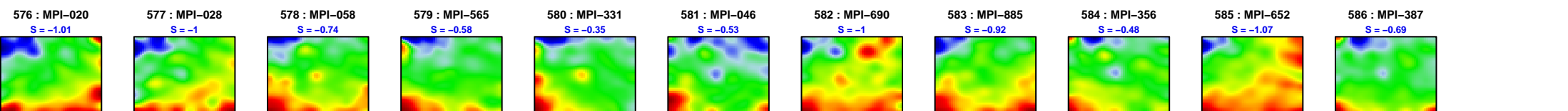

*FL/DLBCL*

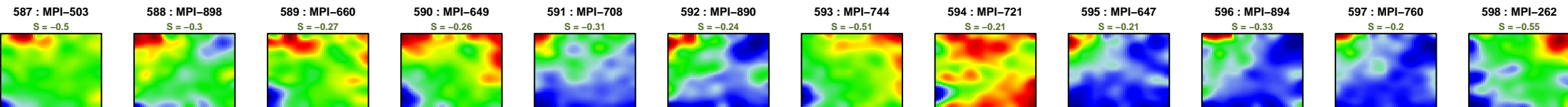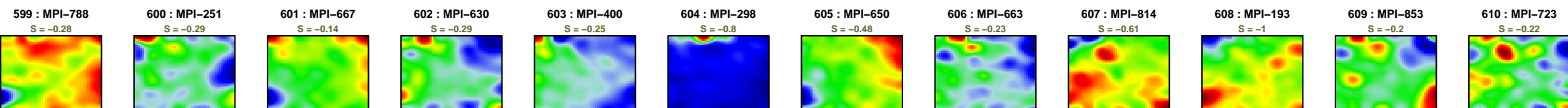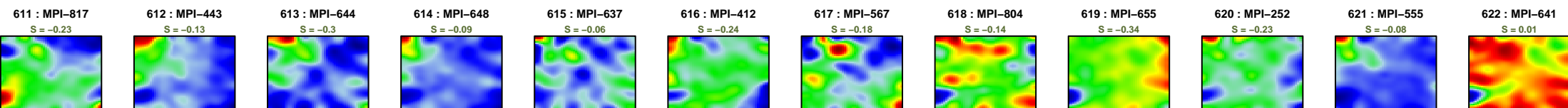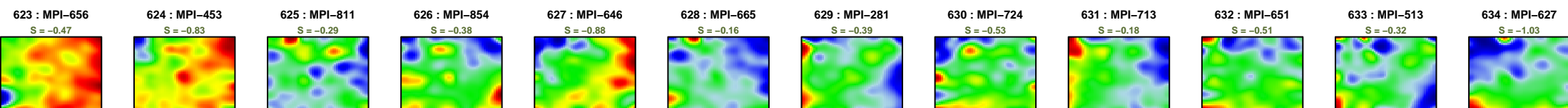

*FL*

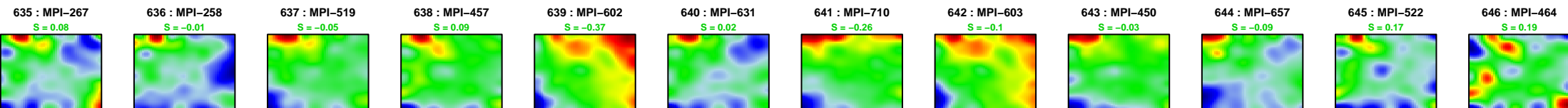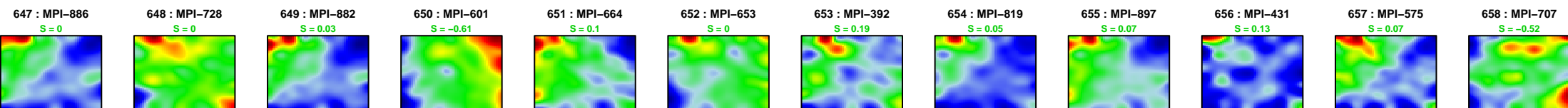

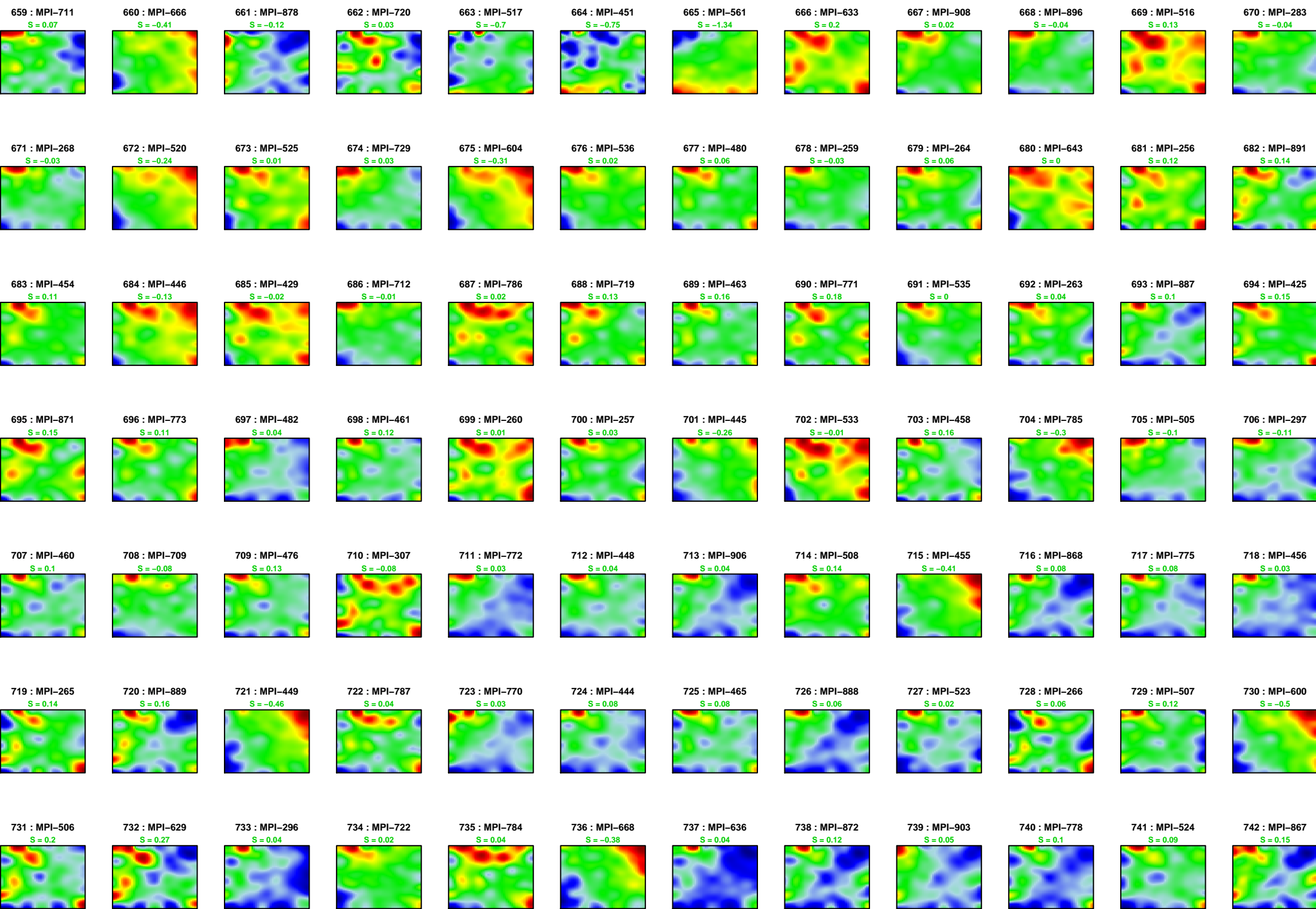

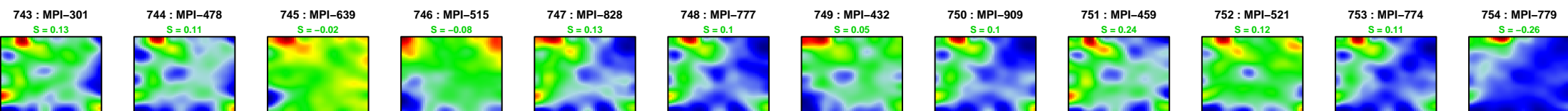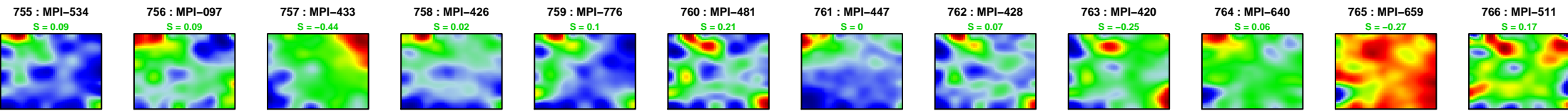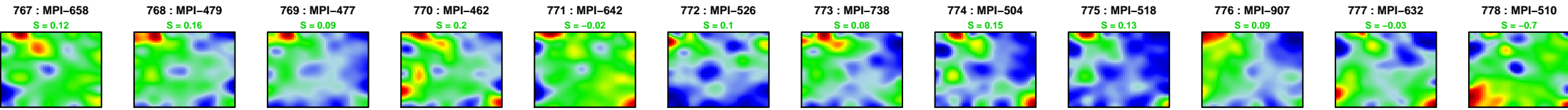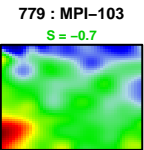

*other*

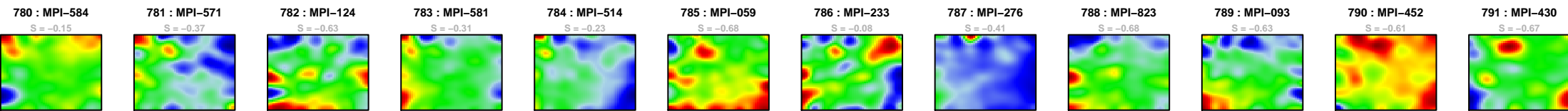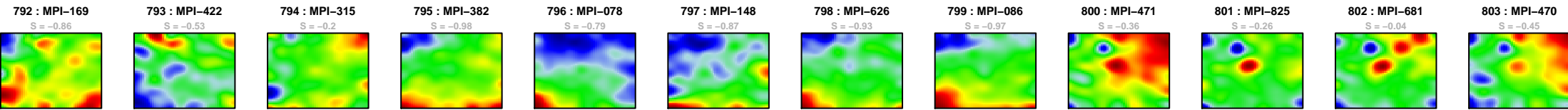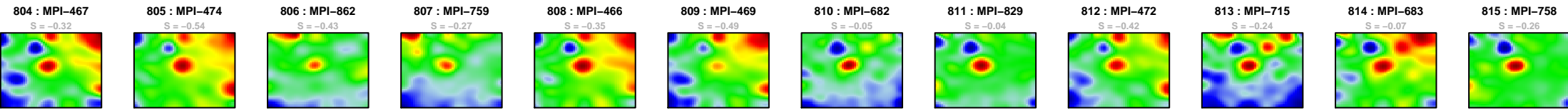

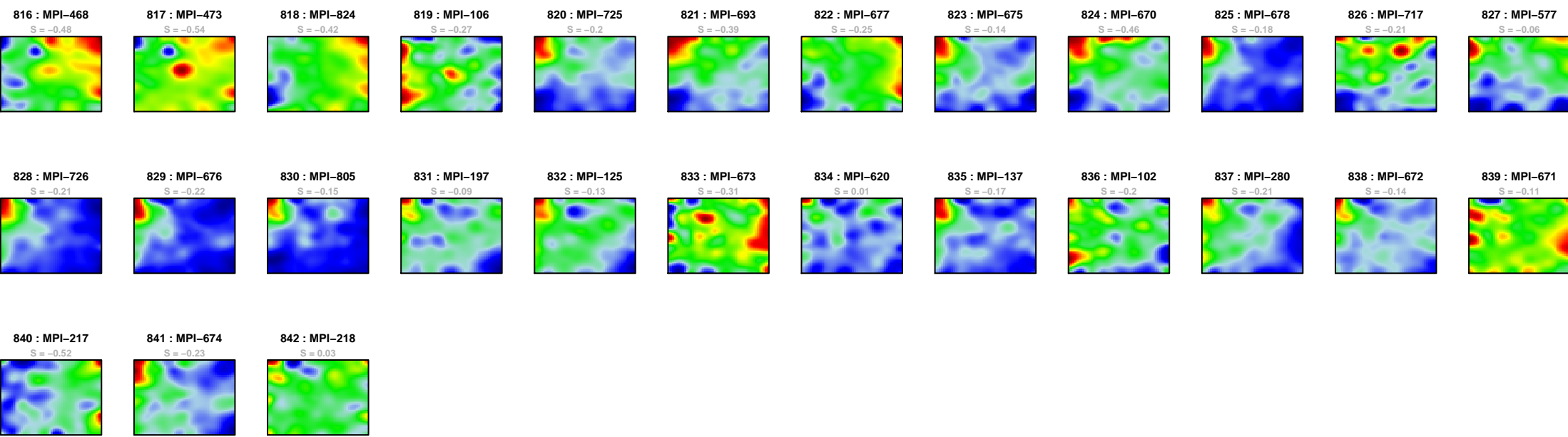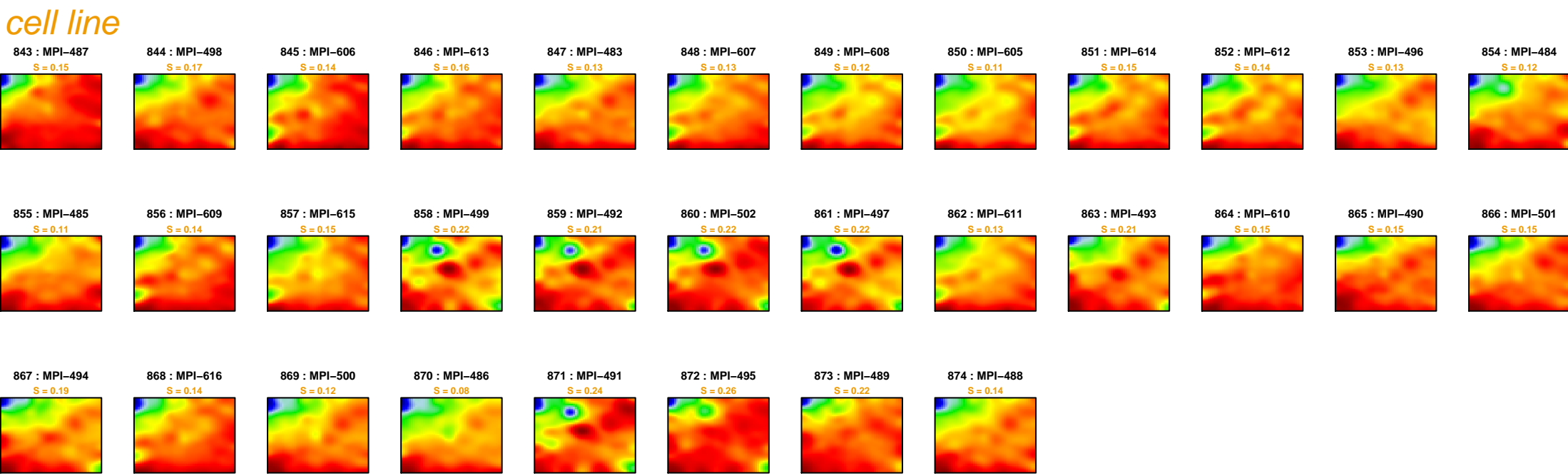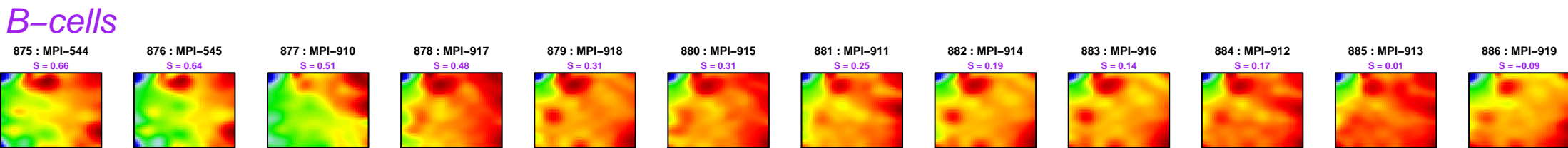

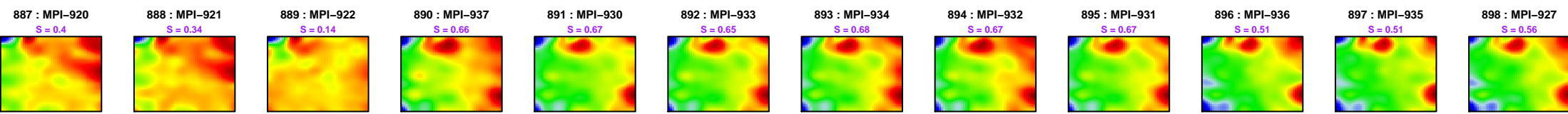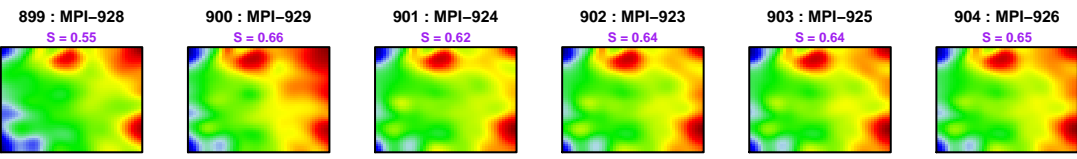

*tonsil*

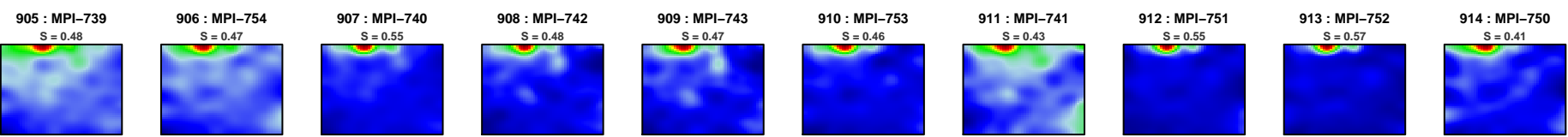

Supplement: Supplementary file 3 — Complete gallery of all 936 sample expression portraits. (PDF 14216 kb) [file 13073_2019_637_MOESM3_ESM.pdf]

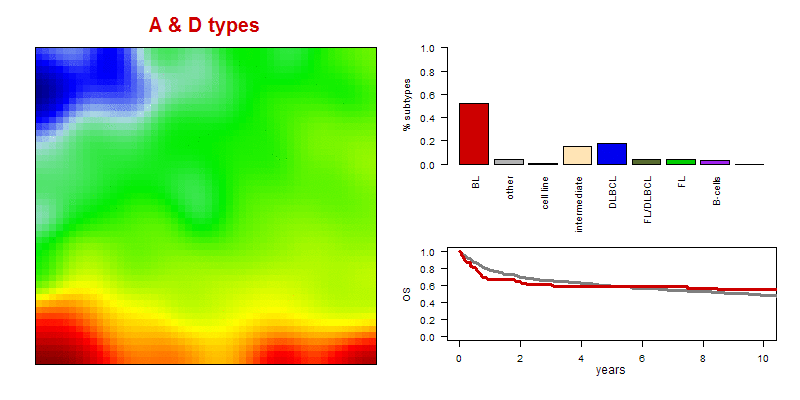

Supplement: Supplementary file 5 — Animated expression portraits of the PATs together with survival curves. (GIF 556 kb) [file 13073_2019_637_MOESM5_ESM.gif]

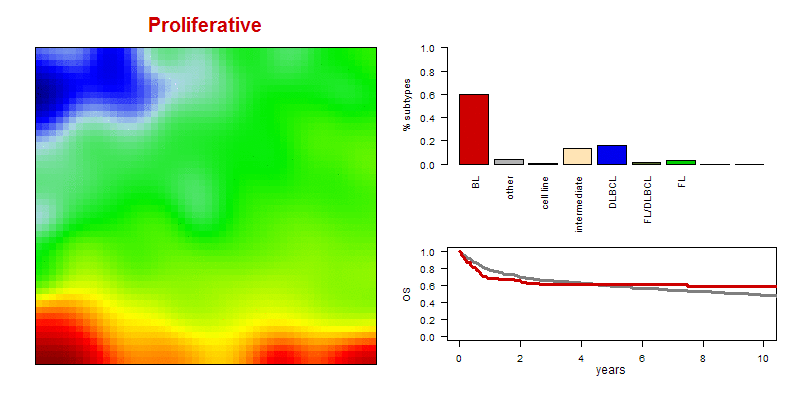

Supplement: Supplementary file 6 — Animated expression portraits of the HTs together with survival curves. (GIF 249 kb) [file 13073_2019_637_MOESM6_ESM.gif]
